# Anti-Inflammatory Activity of Cyclic Imide Derivatives

**DOI:** 10.3390/ph19030457

**Published:** 2026-03-11

**Authors:** Aleksandra Redzicka, Beata Tylińska, Anna Wójcicka

**Affiliations:** 1 Department of Medicinal Chemistry, Faculty of Pharmacy, Wroclaw Medical University, Borowska 211, 50-556 Wrocław, Poland; aleksandra.redzicka@umw.edu.pl; 2 Department of Organic Chemistry and Pharmaceutical Technology, Faculty of Pharmacy, Wroclaw Medical University, Borowska 211A, 50-556 Wrocław, Poland

**Keywords:** cyclic imides, phthalimides, maleimides, succinimides, glutarimides, hydantoins, naphthalimides, anti-inflammatory activity, cyclooxygenase (COX), lipoxygenase (LOX), tumor necrosis factor-α (TNF-α), interleukin-6 (IL-6)

## Abstract

Imide derivatives constitute an interesting group of compounds exhibiting broad biological activity. Structures containing the imide moiety [–CO–N(R)–CO–] occur in both natural and synthetic compounds. Several drugs containing an imide moiety are in therapeutic use. In this review, we present the structures and describe the effects of cyclic imide derivatives, which primarily exhibit anti-inflammatory activity. Some of the presented derivatives have been studied in detail, and their additional analgesic, anticancer, and antibacterial effects have been described. The relative neuroprotective properties of imide derivatives are also described, as are reports of their effect on lowering cholesterol and triglyceride levels. In this review, we discuss monocyclic imide derivatives (succinimide, glutarimide, maleimide, and hydantoin), bicyclic derivatives (e.g., phthalimide), and polycyclic imides.

## 1. Introduction

Cyclic imides constitute an important class of heterocyclic compounds that have emerged as versatile structural scaffolds in the design of bioactive molecules [1]. The properties of imide derivatives largely depend on the nature of the substituents present on the imide ring. Both the size and electrophilic character of these substituents significantly influence the steric properties of the molecule [2]. The presence of specific nitrogen- and oxygen-containing functional groups contributes to their pharmacological activity [3,4]. In addition, imide-based compounds are often electrically neutral and hydrophobic, which facilitates their permeation across biological membranes [2,5].

Structures containing the imide fragment [–CO–N(R)–CO–] occur in both natural and synthetic compounds [6]. This motif has been identified in several natural metabolites, including cladoniamide A, lamprolobine, and migrastatin [7], and it is also present in the alkaloid phyllanthimide, isolated from *Phyllanthus sellowianus*, which serves as a precursor for the synthesis of more complex derivatives [8]. Synthetic cyclic imides exhibit a broad spectrum of biological activities, including antimicrobial effects [9,10,11,12], anticancer activity [13,14,15,16], and carbonic anhydrase inhibition [17], as well as antidiabetic and hypolipidemic properties [18,19,20]. Moreover, they are capable of modulating central nervous system functions [21,22,23] and demonstrate anti-inflammatory activity [24].

**Figure 1:**
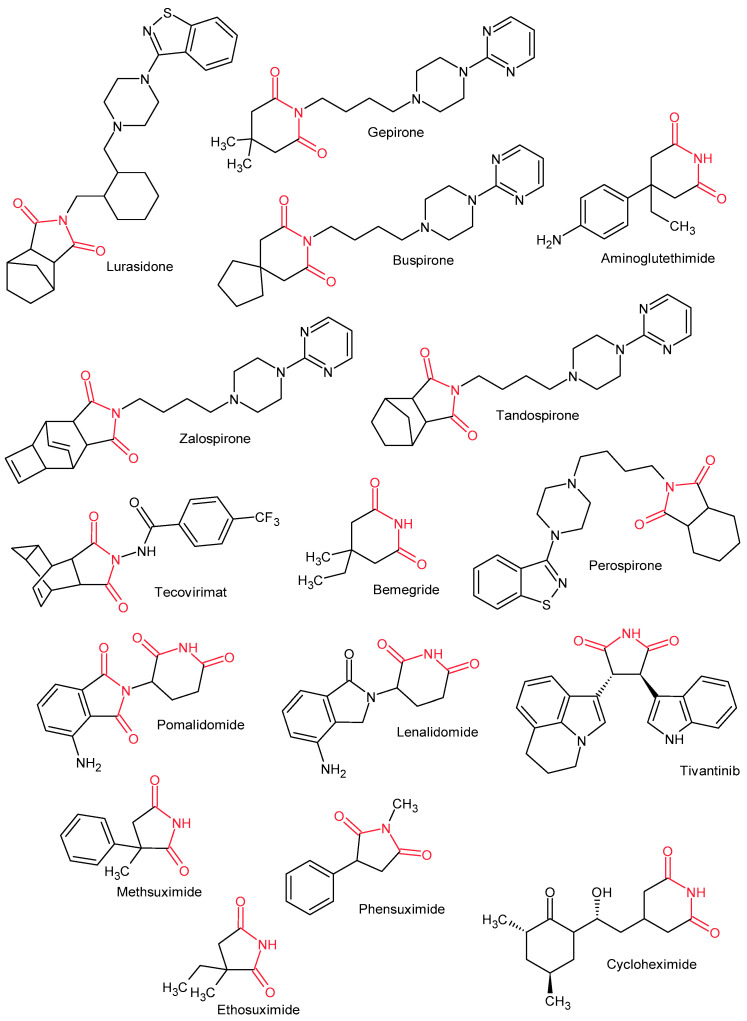
Cyclic imide derivatives used in medicine as drugs.

The clinical relevance of cyclic imides is underscored by their presence in several drugs approved for therapeutic use. These include ethosuximide, phensuximide, and methsuximide, used in the treatment of epilepsy, as well as antipsychotic agents such as lurasidone, perospirone, tandospirone, gepirone, and zalospirone [6] (Figure 1). The imide ring motif is also present in anticancer agents, including aminoglutethimide [6], pomalidomide [6], lenalidomide, and tivantinib [25] (Figure 1). The glutarimide scaffold occurs in the structures of the antifungal antibiotic cycloheximide and the respiratory stimulant bemegride [26]. Furthermore, the imide fragment is found in tecovirimat [27], the first antiviral drug approved for the treatment of smallpox.

**Figure 2:**
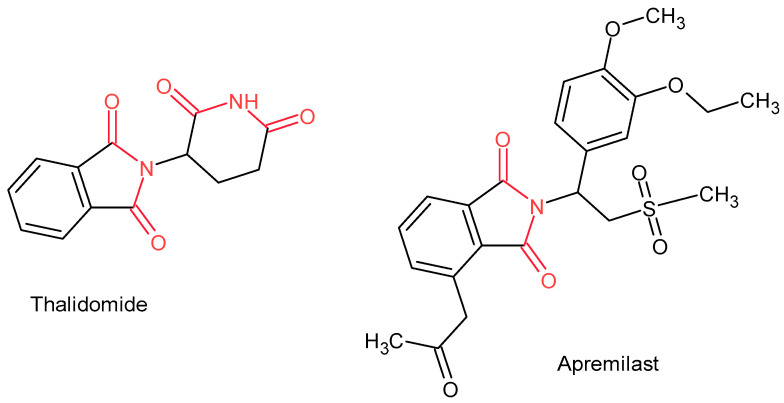
Cyclic imides are used as anti-inflammatory agents.

Among imide-containing drugs, anti-inflammatory properties are exhibited by thalidomide—a potent inhibitor of tumor necrosis factor-α (TNF-α) production [28]—and apremilast, a selective phosphodiesterase 4 (PDE4) inhibitor approved for the treatment of psoriasis and psoriatic arthritis [29] (Figure 2).

Inflammation is a tightly regulated physiological response to tissue injury and pathogenic challenges, aimed at eliminating the initiating stimulus and restoring homeostasis. The inflammatory process involves coordinated vascular and cellular events, including increased blood flow, enhanced vascular permeability, and recruitment of leukocytes to the site of injury [1,30]. During the inflammatory response, immune cells release mediators such as cytokines (IL-1β, IL-6, and TNF-α), eicosanoids, and reactive oxygen and nitrogen species [31]. Key molecular pathways regulating the expression of pro-inflammatory mediators include NF-κB activation via Toll-like receptor 4 (TLR4) and the arachidonic acid cascade mediated by cyclooxygenase isoenzymes COX-1 and COX-2, leading to the production of pro-inflammatory prostaglandins, particularly PGE_2_ [31,32]. Targeting pro-inflammatory cytokines such as IL-1β, IL-6, and TNF-α, as well as COX-2 and PDE4, remains a central strategy in contemporary anti-inflammatory drug discovery [1,31,32].

Although inflammation is essential for host defense, its unresolved or excessive form contributes to the pathogenesis of numerous chronic diseases, including neurodegenerative, autoimmune, and metabolic disorders [33]. These observations highlight the urgent need for the development of novel anti-inflammatory agents capable of selectively modulating key signaling pathways.

In this context, cyclic imides represent a promising class of compounds whose structural diversity enables the design of molecules with targeted anti-inflammatory activity. The principal subclasses include monocyclic imides (succinimides, glutarimides, and maleimides), bicyclic imides (e.g., phthalimides), and polycyclic imides (e.g., naphthalimides) [34].

This review aims to present the current state of knowledge regarding cyclic imides with anti-inflammatory activity, with particular emphasis on their chemical structures, subclass classification, and potential therapeutic applications.

## 2. Cyclic Imides with Anti-Inflammatory Activities

Cyclic imides exhibiting anti-inflammatory activity encompass structurally diverse subclasses, including monocyclic compounds, bicyclic derivatives, and more complex polycyclic systems.

### 2.1. Monocyclic Imide Derivatives

#### 2.1.1. Succinimide Derivatives

Succinimides are heterocyclic compounds containing a pyrrolidine-2,5-dione scaffold. Succinimides are used as antiepileptic drugs (phensuximide, methsuximide, and ethosuximide), antipsychotic drugs (tandospirone, lurasidone, and perospirone), and as drugs for treating smallpox (tecovirimat) and cancer (tivantinib) (Figure 1) [35]. Succinimide derivatives have not yet been introduced in the treatment of anti-inflammatory diseases, but many studies are being conducted on the synthesis of new derivatives of these systems with anti-inflammatory activity.

**Figure 3:**
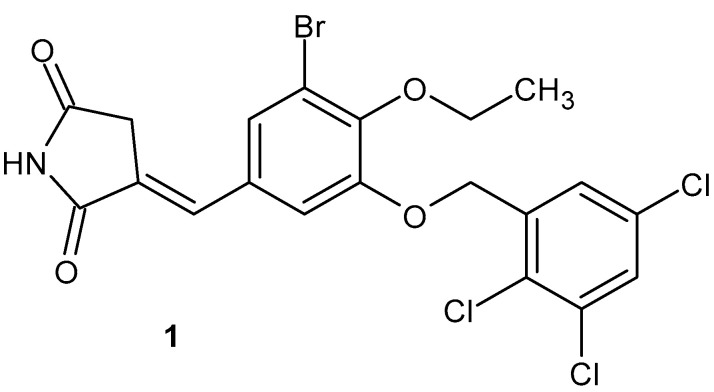
(*E*)-3-(3-bromo-4-ethoxy-5-((2,3,5-trichlorobenzyl)oxy)-benzylidene)pyrrolidine-2,5-dione **1** reported by Zhang et al. [36].

3-Benzylidene succinimide analogues were evaluated by Zhang et al. [36] as potent spleen tyrosine kinase (Syk) inhibitors. The most active was (*E*)-3-(3-bromo-4-ethoxy-5-((2,3,5-trichlorobenzyl)oxy)-benzylidene)pyrrolidine-2,5-dione **1** (Figure 3) with a Syk-inhibitory IC_50_ = 1.3 µM. Derivative **1** demonstrated the potential to inhibit IL-6 and MMP-3 secretion, as well as oral efficacy in a mouse model of collagen-induced arthritis (CIA). Compound **1** was tested for its effect in a mouse CIA model, which mirrored the clinical progression of human rheumatoid arthritis. Administration of derivative **1** alleviated typical symptoms of acute inflammation, such as edema and swelling [36].

**Figure 4:**
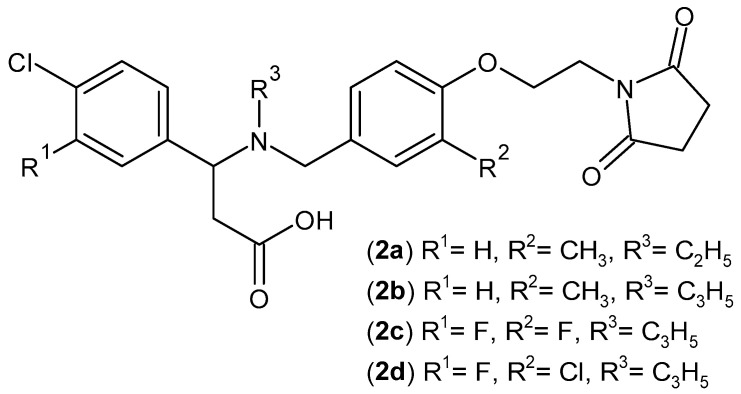
CXCR3 antagonists **2a**–**d** reported by Bata et al. [37].

CXCR3 is a chemokine receptor that participates in the recruitment of inflammatory cells. *N,N*-disubstituted benzylamine derivatives with succinimide scaffolds **2a**–**d** (Figure 4) were evaluated as CXCR3 antagonists. Compound **2a** (IC_50_ = 21 nM) proved to be the most effective in the initial assay. However, considering the Caco-2 permeability profile and hERG safety, compounds **2b** (IC_50_ = 36 nM), **2c** (IC_50_ = 58 nM), and **2d** (IC_50_ = 40 nM), showing an hERG IC50 > 10 μM and no micronucleus acid test (MNT) and mutagenicity assay (AMES II) alerts, were considered the best candidates for further studies [37].

**Figure 5:**
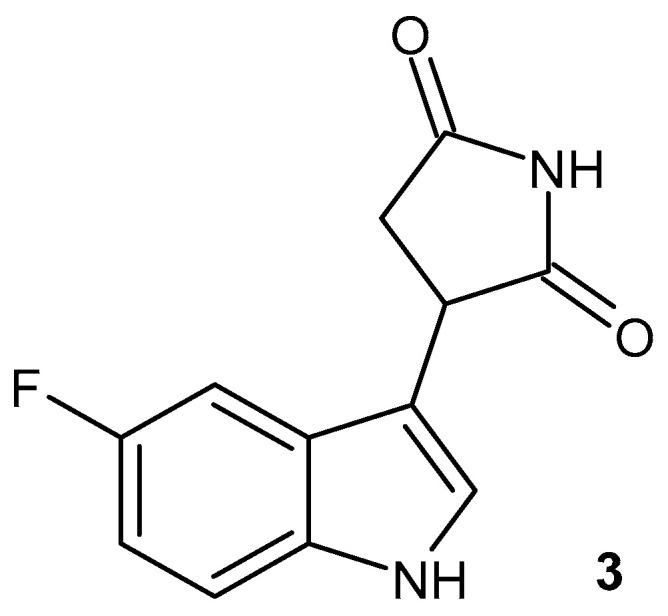
3-(5-Fluoro-1*H*-indol-3-yl)pyrrolidine-2,5-dione **3** as hIDO-1 inhibitor reported by Crosignani et al. [38].

Indoleamine-pyrrole-2,3-dioxygenase (IDO) is an immunomodulatory enzyme involved in tryptophan catabolism. It inhibits T and NK lymphocytes and generates Treg lymphocytes. IDO expression is induced by interferon gamma in response to inflammatory stimuli. COX-2 inhibitors reduce the activity of indoleamine 2,3-dioxygenase, which leads to a reduction in the activity of pro-inflammatory cytokines. 3-(3-Indolyl)-succinimide and its derivatives were obtained as hIDO-1 inhibitors. Replacements of the indole core with alternative heterocycles (naphthalene, indazole, benzimidazole, azaindole, isoquinoline, quinoline, indole, substituted benzofurans, and benzothiophenes) were found to be significantly less active. The most active, 3-(5-fluoro-1*H*-indol-3-yl)pyrrolidine-2,5-dione **3** (Figure 5), was selected to advance to clinical trials as a novel hIDO-1 inhibitor (a First in Patient Study in Malignant Gliomas—NCT02764151). After analyzing all available data from enrolled patients, the sponsor decided to terminate the study and not apply for marketing authorization for the drug in the indication of malignant glioma [38].

**Figure 6:**
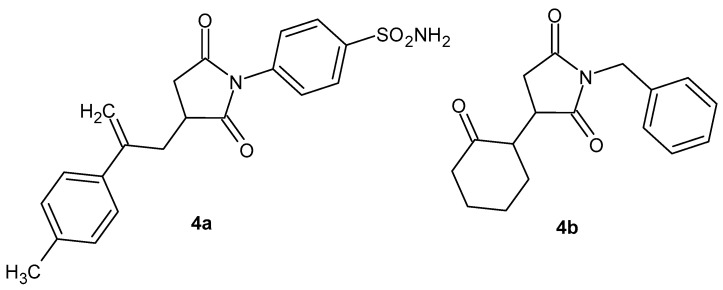
*N*-substituted pyrrolidine-2,5-dione derivatives **4a**–**b** reported by Jan et al. [39].

Anti-inflammatory activity of *N*-substituted pyrrolidine-2,5-dione derivatives was determined using different in vitro assays like cyclooxygenase-1 (COX-1), cyclooxygenase-2 (COX-2), 5-lipoxygenase (5-LOX), anti-protease, and albumin denaturation assays [39], and the compounds exhibited good to excellent inhibition of 5-LOX and selectivity against COX-2 isozyme. The anti-inflammatory activity of the most potent inhibitors of COX-2 (Figure 6), **4a** (IC_50_ = 0.98 µM) and **4b** (IC_50_ = 19.98 µM), was assessed using a carrageenan-induced paw edema model in mice with various mediators like histamine, bradykinin, prostaglandin (PGE_2_), and leukotriene. The tested compounds, **4a**–**b**, showed less activity in bradykinin and histamine-induced inflammation as compared to the positive control. Compounds **4a**–**b** significantly reduced the PGE2-induced paw edema (by 64.60% and 81.80% until the fifth hour of the procedure). The effect of derivative **4a** on paw edema induced by leukotriene was comparable to that of the standard drug, montelukast. In vivo acute toxicity testing demonstrated the safety of compounds **4a**–**b** at doses up to 1000 mg/kg [39].

**Figure 7:**
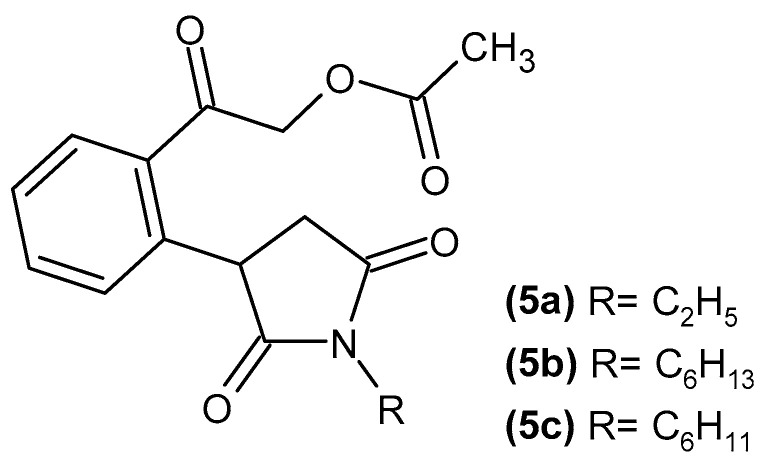
*N*-alkil-3-phenylpyrrolidine-2,5-dione derivatives **5a**–**c** reported by Sivakumar et al. [40].

Protein denaturation is a key indicator of inflammatory processes, and compounds that can effectively prevent this denaturation are promising candidates for anti-inflammatory therapies. Sivakumar et al. [40] obtained 2-(2-(1-alkyl-2,5-dioxopyrrolidin-3-yl)phenyl)-2-oxoethyl acetate derivatives, **5a**–**c** (Figure 7), which exhibited greater inhibition of protein denaturation (using the egg albumin denaturation method) than the anti-inflammatory drug diclofenac.

#### 2.1.2. Glutarimide Derivatives

Glutarimide derivatives are heterocyclic compounds with a piperidine-2,6-dione ring. Glutarimides exhibit a broad spectrum of pharmacological activity, and the piperidine-2,6-dione scaffold is present in some drugs, e.g., cykloheksimide, gepirone, buspirone, bemegride, and aminoglutethimide (Figure 1) [26].

**Figure 8:**
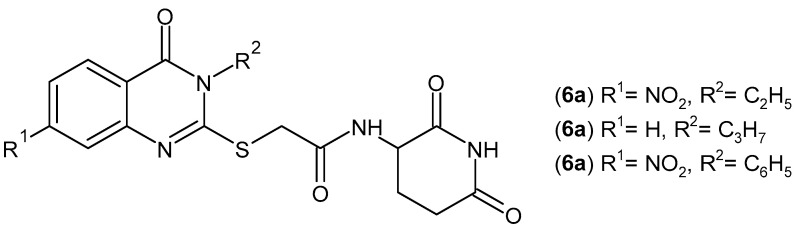
Quinazolinone derivatives with a glutarimide moiety **6a**–**c** reported by Abdallah et al. [41].

Glutarimide derivatives with a quinazolinone moiety have been synthesized by Abdallah et al. [41] as potent inhibitors of the pro-inflammatory factors TNF-α and IL-6. The most potent compounds, **6a**–**c** (Figure 8), also showed inhibitory activity on both COX-1 and COX-2 and were more effective than thalidomide. The most active compound, **6a**, showed a significant reduction in TNF-α and IL-6 levels, comparable to dexamethasone and more effective than thalidomide.

**Figure 9:**
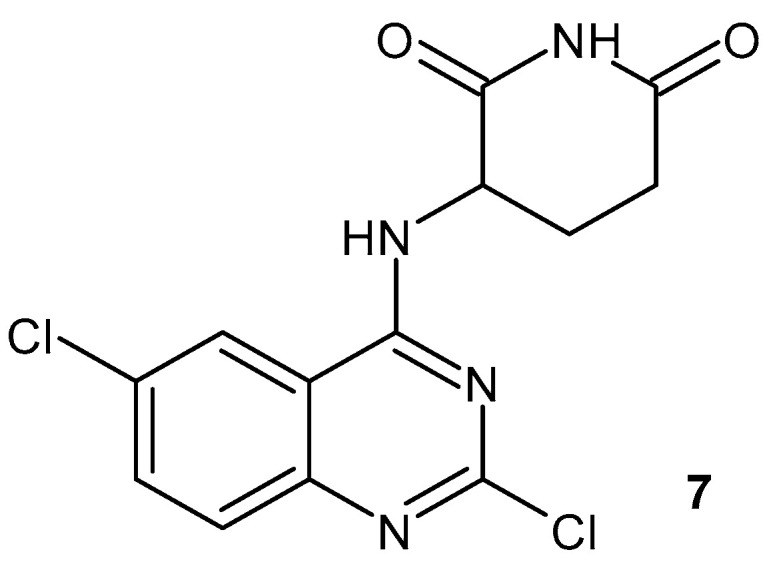
3-((2,6-Dichloroquinazolin-4-yl)amino)piperidine-2,6-dione **7** reported by Abdallah et al. [42].

Other glutarimide derivatives with a quinazoline scaffold were synthesized and evaluated for their activities on the pro-inflammatory factors TNF-α and IL-6 using thalidomide and dexamethasone as reference drugs [42]. 3-((2,6-Dichloroquinazolin-4-yl)amino)piperidine-2,6-dione **7** (Figure 9) exhibited significant reductions in TNF-α (76.14%) and IL-6 (80.65%), stronger than thalidomide and dexamethasone. This compound, **7**, also inhibited COX-1 (IC_50_ values of 0.85 μM) and COX-2 (IC_50_ values of 0.06 μM) and is more potent than thalidomide (IC_50_ values of 4.57 μM and 1.38 μM, respectively).

**Figure 10:**
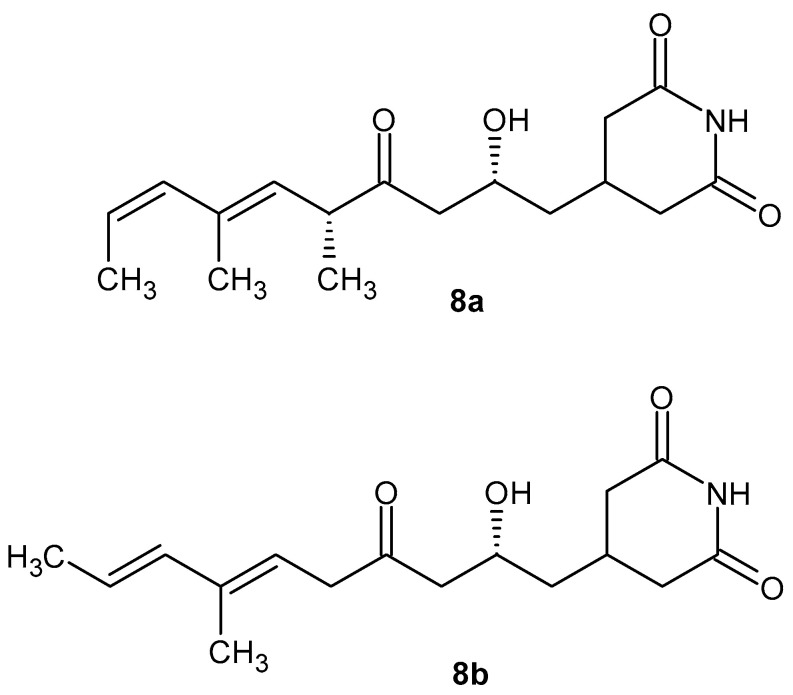
9-Methylstreptimidone **8a** and S632A3 **8b** reported by Ishikawa et al. [44].

Glutarimide-containing polyketides (GPs) are a large group of natural products isolated from various microorganisms, primarily from *Streptomycetes.* GPs exhibit antifungal, antibacterial, anticancer, and anti-inflammatory properties [43]. 9-Methylstreptimidone **8a** (Figure 10) was isolated from *Streptomyces species* as a novel inhibitor of NF-κB [44]. The synthetic thioester analogs of compound **8a** also inhibit LPS-induced NO production comparable to that of 9-methylstreptimidone **8a** [44]. Glutarimide antibiotic S632A3 **8b** (Figure 10) isolated from *Streptomyces hygroscopicus S632* exhibits good anti-inflammatory activity. S632A3 **8b** inhibits NF-κB transcription activity induced by LPS and the expression levels of LPS-induced pre-inflammatory factors such as iNOS, COX-2, TNF-α, and IL-6 by inhibiting GSK-3β and the related ASK1-p38 signaling pathway in Raw264.7 cells [45].

**Figure 11:**
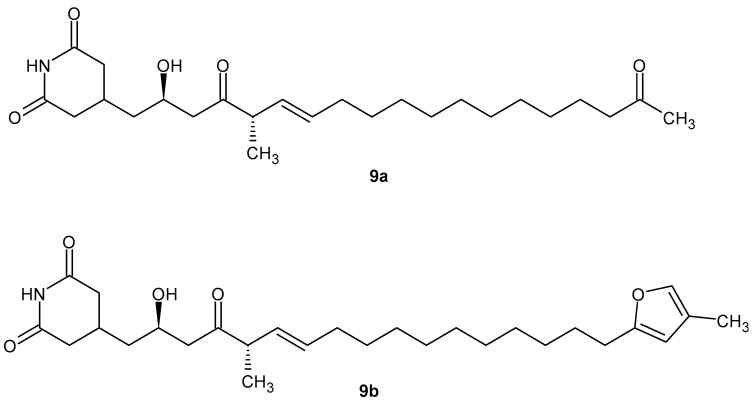
Gladiofungins C **9a** and E **9b** reported by Chen et al. [46].

Gladiofungins C **9a** and E **9b**, isolated by Chen et al. [46] from *Burkholderia gladioli* (Figure 11), showed moderate anti-inflammatory activities for their inhibition of NO production in LPS-induced RAW 264.7 macrophages.

**Figure 12:**
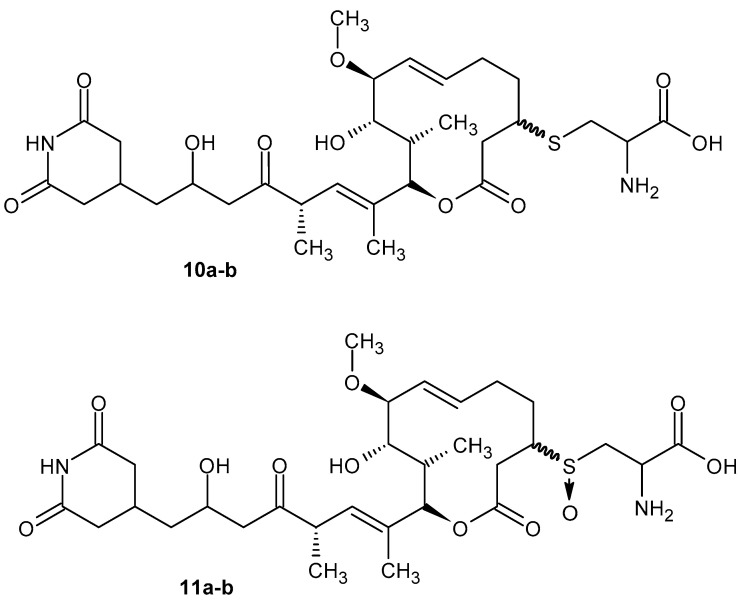
NK30424A/B **10a**–**b** and their sulfoxide derivatives, **11a**–**b**, reported by Takayasuet al. [47].

Glutarimides with macrolide in side chains NK30424A and NK30424B **10a**–**b** (Figure 12) as isomers isolated from *Streptomycetes* exhibit anti-inflammatory activity by inhibiting lipopolysaccharide-induced TNF-α production [47]. Next, new sulfoxide derivatives of NK30424A/B, as well as stereoisomers **11a**–**b**, were synthesized (Figure 12). These diastereoisomers, **11a**–**b**, have more powerful anti-inflammatory activity [48].

**Figure 13:**
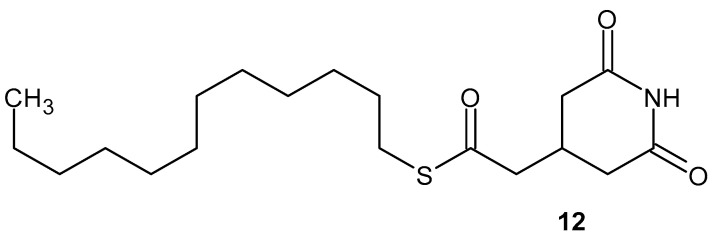
Structure of DTCM-G **12** reported by Ishikawa et al. [44].

3-[(dodecylthiocarbonyl)methyl] glutarimide DTCM-G **12** (Figure 13), synthesized by Ishikawa et al. [44], exhibits anti-inflammatory activity by inhibiting LPS-induced NO production, AP-1 activation, and the expression of iNOS and COX-2 in a mouse macrophage cell line, RAW264.7. DTCM-G **12** does not inhibit LPS-induced NF-κB activation or LPS-induced IL-6 secretion [49]. DTCM-G **12** is highly effective in inhibiting sodium dextran sulfate-induced colon inflammation in rats [50].

#### 2.1.3. Hydantoin Derivatives

**Figure 14:**
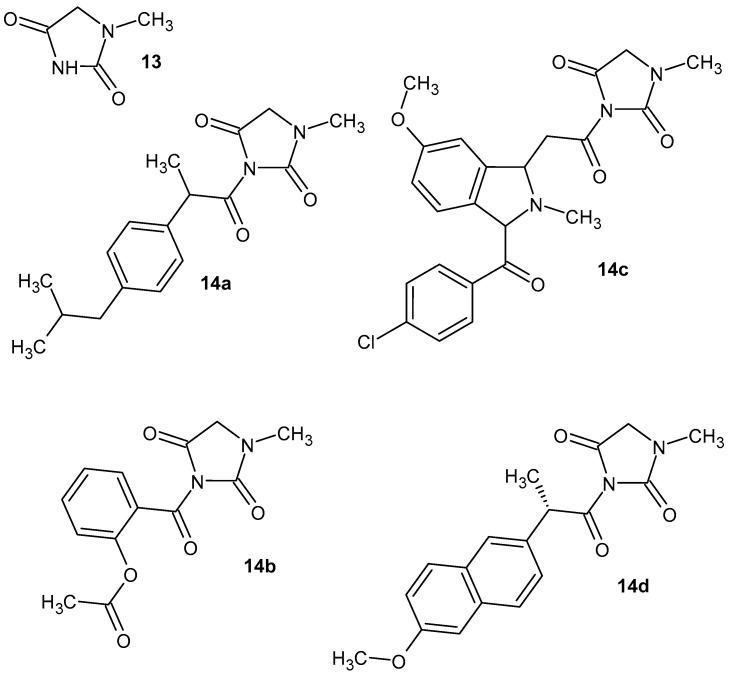
1-Methylhydantoin **13** and its derivatives, **14a**–**d**, reported by Xu et al. [51].

Hydantoin derivatives contain an imidazolidine-2,4-dione ring and exhibit a variety of biological activities, including anti-inflammatory activity. 1-Methylhydantoin **13** (Figure 14) was isolated from *Oviductus ranae* [51] and exhibited a good anti-inflammatory effect by reducing ear edema in mice caused by xylene [52]. Next, 1-methylhydantoin conjugates with various molecules with anti-inflammatory activity, ibuprofen, aspirin, indomethacin, and naproxen, were synthesized (**14a**–**d**) (Figure 14). The anti-inflammatory assay showed that compound **14b** exhibited an enhanced anti-inflammatory effect in acute pneumonia induced by particulate matter (PM2.5) compared to the reference compounds: 1-methylhydantoin and aspirin [51].

**Figure 15:**
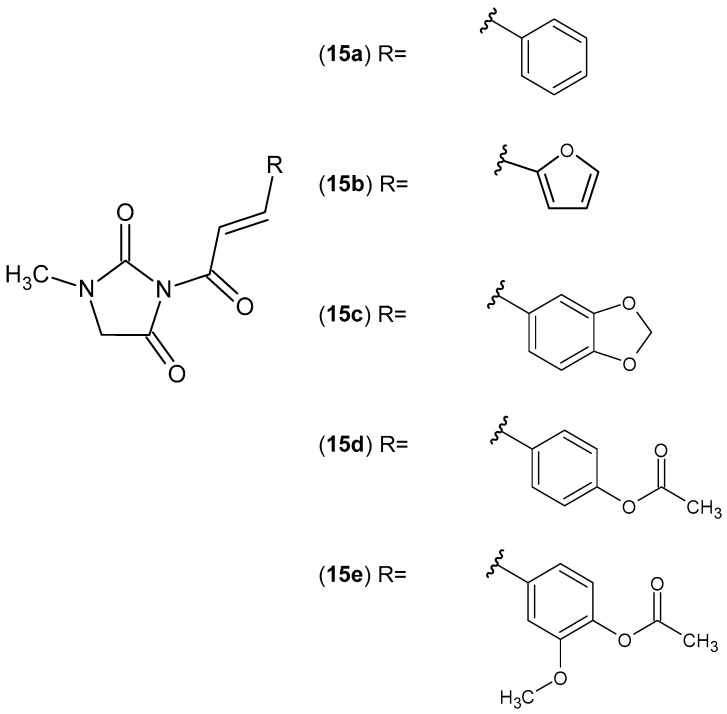
1-Methylhydantoin cinnamic imides **15a**–**e** reported by Wang et al. [52].

The anti-inflammatory activity of 1-methylhydantoin cinnamic imides **15** (Figure 15) was investigated. All derivatives, **15a**–**e**, showed obvious anti-inflammatory activity by reducing the degree of ear swelling in mice. Compounds **15b** and **15d** best inhibited the secretion of inflammatory factors IL-1β and TNF-α, as well as NO release [52].

**Figure 16:**
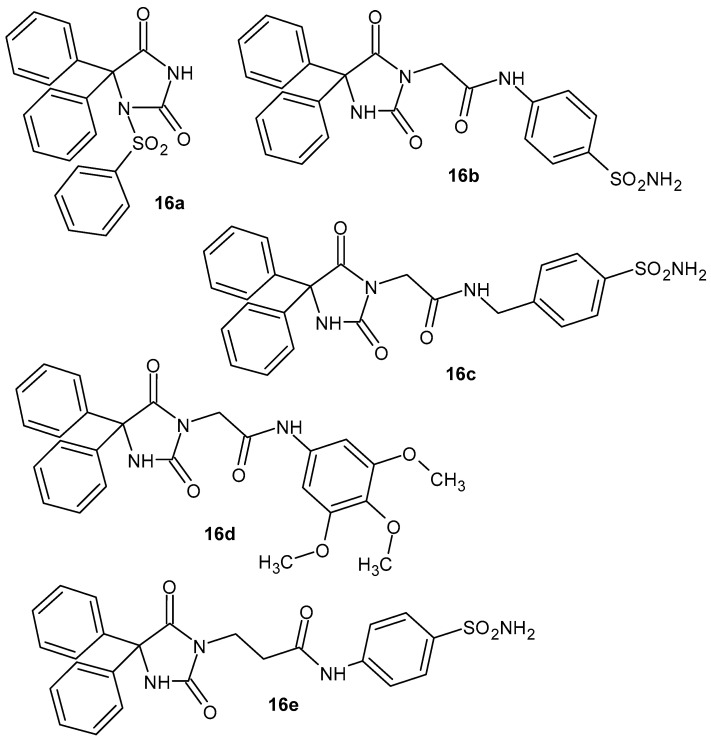
5,5-Diphenylimidazolidine-2,4-dione derivatives **16a**–**e** reported by Abdel-Aziz et al. [54].

1-Benzenesulfonyl-5,5-diphenylhydantoine **16a** (Figure 16) exhibits anti-inflammatory activity as a prostaglandin synthetase inhibitor [53]. Next, other 5,5-diphenylimidazolidine-2,4-dione derivatives with potent anti-inflammatory activity were synthesized by Abdel-Aziz et al. [54]. Among them, compounds **16b**–**e** (Figure 16) exhibited a high COX-2 inhibitory effect (IC_50_ = 0.70, 0.44, 0.61, and 0.41 mM, respectively) and a better selectivity index (range of 142–243) compared to celecoxib [54].

**Figure 17:**
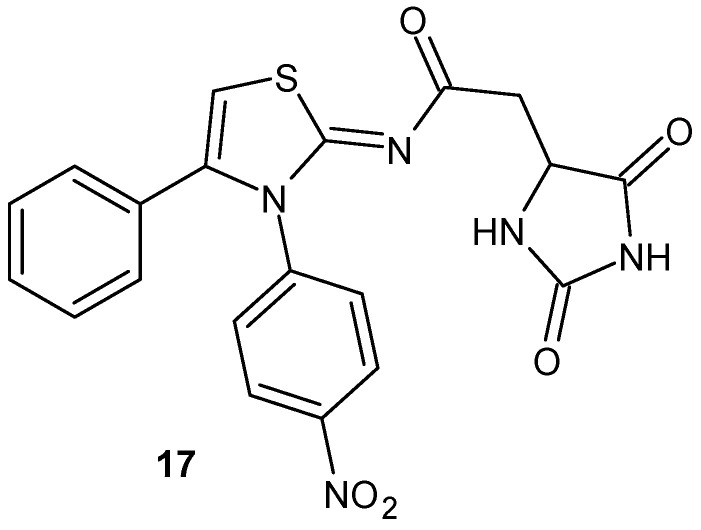
2-(2,5-Dioxoimidazolidin-4-yl)-*N*-(3-(4-nitrophenyl)-4-phenylthiazol-2(3*H*)-ylidene)acetamide **17** reported by Sondhi et al. [55].

2-(2,5-Dioxoimidazolidin-4-yl)-*N*-(3-(4-nitrophenyl)-4-phenylthiazol-2(3*H*)-ylidene) acetamide **17** (Figure 17), synthesized by Sondhi et al. [55], showed good anti-inflammatory activity in the carrageenan-induced paw edema test, similar to ibuprofen (34% at 50 mg/kg p.o.).

**Figure 18:**
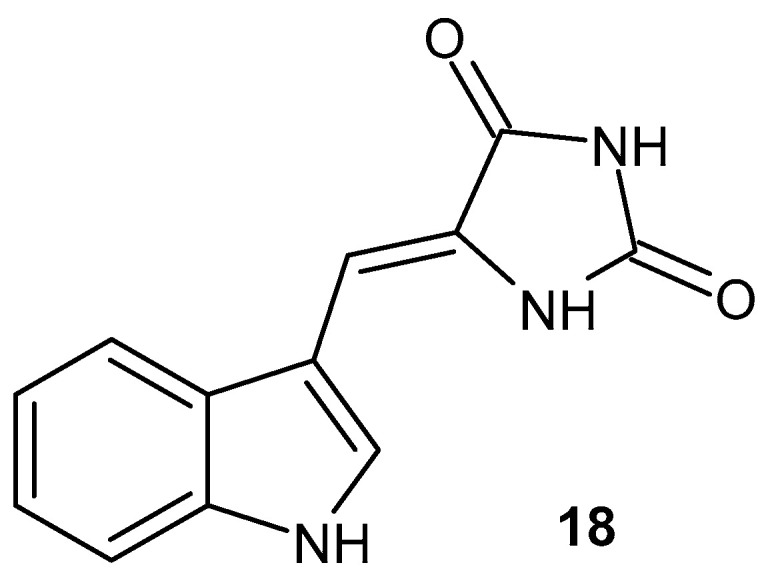
(Z)-5-(1*H*-indole-3-ylmethylene) imidazolidine-2,4-dione **18** reported by Lin et al. [56].

Lin et al. [56] synthesized indole-hydantoin derivative **18** (Figure 18) with anti-inflammatory activity. (Z)-5-(1*H*-indole-3-ylmethylene) imidazolidine-2,4-dione **18** significantly inhibits the LPS-induced production of NO, secretion of chemokines (CCL2 and CXCL1), and activation of NF-κB [56].

**Figure 19:**
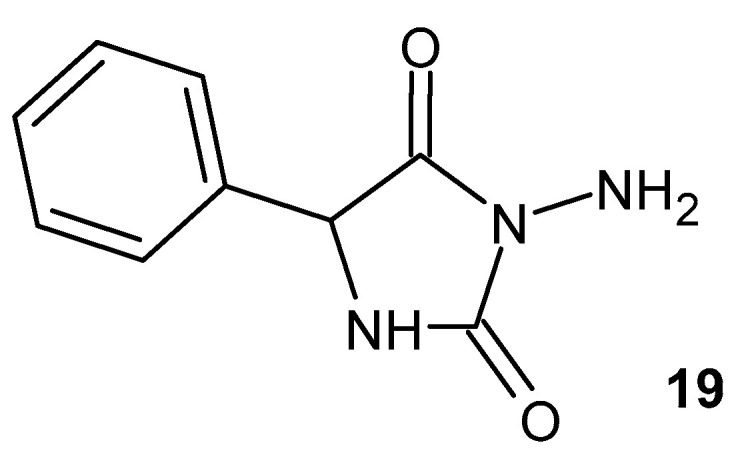
3-Amino-5-benzylimidazolidine-2,4-dione **19** reported by Mani et al. [57].

3-Amino-5-benzylimidazolidine-2,4-dione **19** (Figure 19) exhibits anti-inflammatory effects by reducing the increase in the expression of pro-inflammatory markers (iNOS and IL-1β) induced by LPS [57].

**Figure 20:**
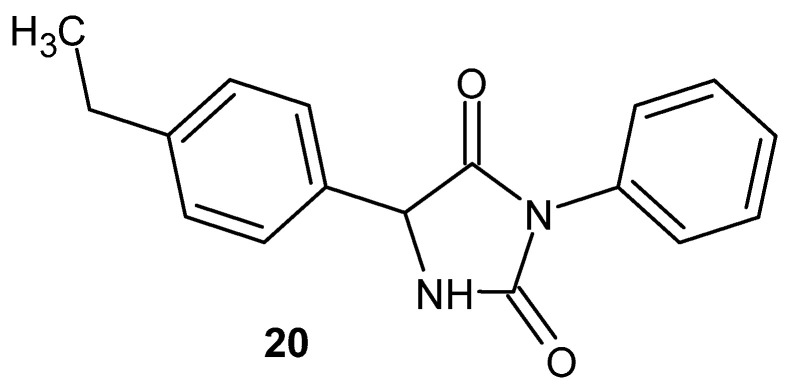
3-Phenyl-5-(4-ethylphenyl)imidazolidine-2,4-dione **20** reported by De Queirozet al. [58].

3-Phenyl-5-(4-ethylphenyl)imidazolidine-2,4-dione **20** (Figure 20) shows antinociceptive effects in the acetic acid-induced writhing test in mice, mediated by anti-inflammatory mechanisms (probably by reducing the level of pro-inflammatory cytokines: TNF-α and IL-1) [58].

**Figure 21:**
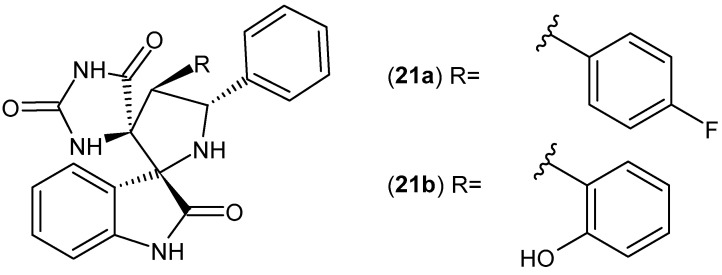
Spirooxindolopyrrolidine-hydantoins **21a**–**b** reported by Toumi et al. [59].

A series of spirooxindolopyrrolidine-hydantoins **21** was obtained and evaluated for their anti-inflammatory activity by Toumi et al. [59]. Derivatives **21a**–**b** (Figure 21) significantly inhibited the lipoxygenase-5 (LOX-5) enzyme, more potently than diclofenac sodium (IC_50_ = 1.09 and 1.01 mg/mL, respectively). Compounds **21a**–**b** also showed better anti-inflammatory effects than diclofenac in the carrageenan-induced rat paw edema test [59].

**Figure 22:**
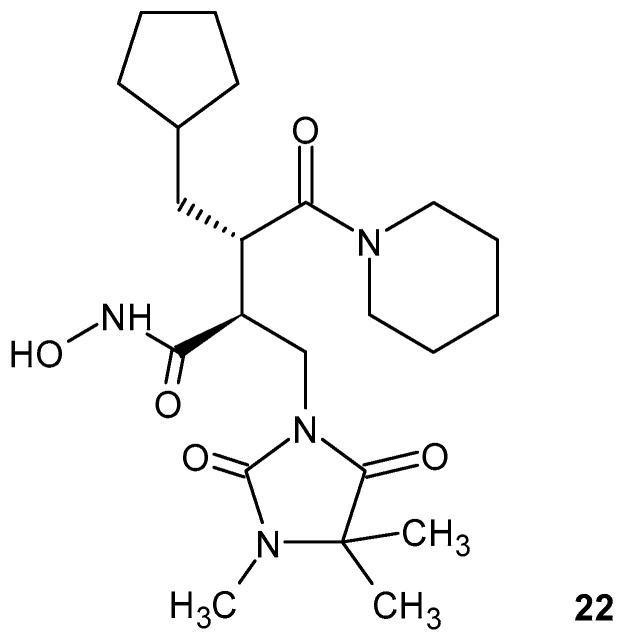
Ro-32-3555 **22** reported by Lewis et al. [63].

Cipemastat (Ro-32-3555) **22** (Figure 22), developed by Roche, is a selective inhibitor of matrix metalloproteinases (MMPs) and is used for rheumatoid arthritis treatment [60,61,62]. (2R,3R)-3-(cyclopentylmethyl)-N-hydroxy-4-oxo-4-piperidin-1-yl-2-[(3,4,4-trimethyl-2,5-dioxoimidazolidin-1-yl)methyl]butanamide **22** inhibits interleukin-1-induced cartilage collagen degradation [63].

**Figure 23:**
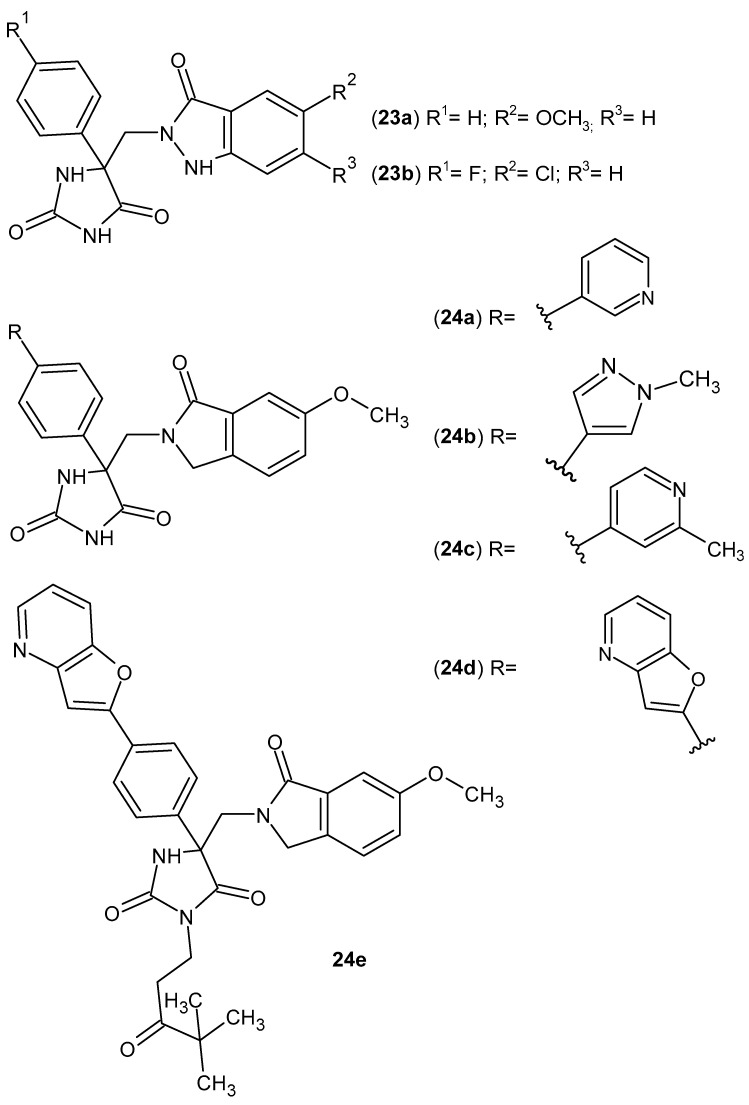
TACE inhibitors **23**–**24** reported by Yu et al. [65].

TNF-α converting enzyme (TACE) is a metalloproteinase that converts the membrane-bound precursor of TNFα into a soluble component. TACE inhibitors modulate TNF-α levels and may therefore have potential in the treatment of related inflammatory diseases. Studies have shown that the imide nitrogen of the hydantoin moiety binds to the active site of the enzyme [64]. A series of hydantoin derivatives were synthesized as potent TACE inhibitors. Among them, most active were derivatives containing indazol-3-one **23a**–**b** and isoindol-1-one **24a**–**d** scaffolds (Figure 23) [64,65]. Subsequently, to improve the pharmacokinetic properties of compound **24d**, a series of *N*-substituted derivatives were prepared, of which pivalate derivative **24e** (Figure 23) demonstrated the best bioavailability [66].

#### 2.1.4. Maleimide Derivatives

Maleimide is an example of a cyclic dicarboximide in which two carbonyl groups bound to the nitrogen atom form a 1*H*-pyrrole-2,5-dione structure. These structures constitute an important pharmacophore in the design of compounds with potential anti-inflammatory activity. The anti-inflammatory activity of maleimide derivatives is associated both with the presence of the maleimide ring itself and with substituents at the nitrogen atom or at the 3- and 4-positions of the ring [67].

**Figure 24:**
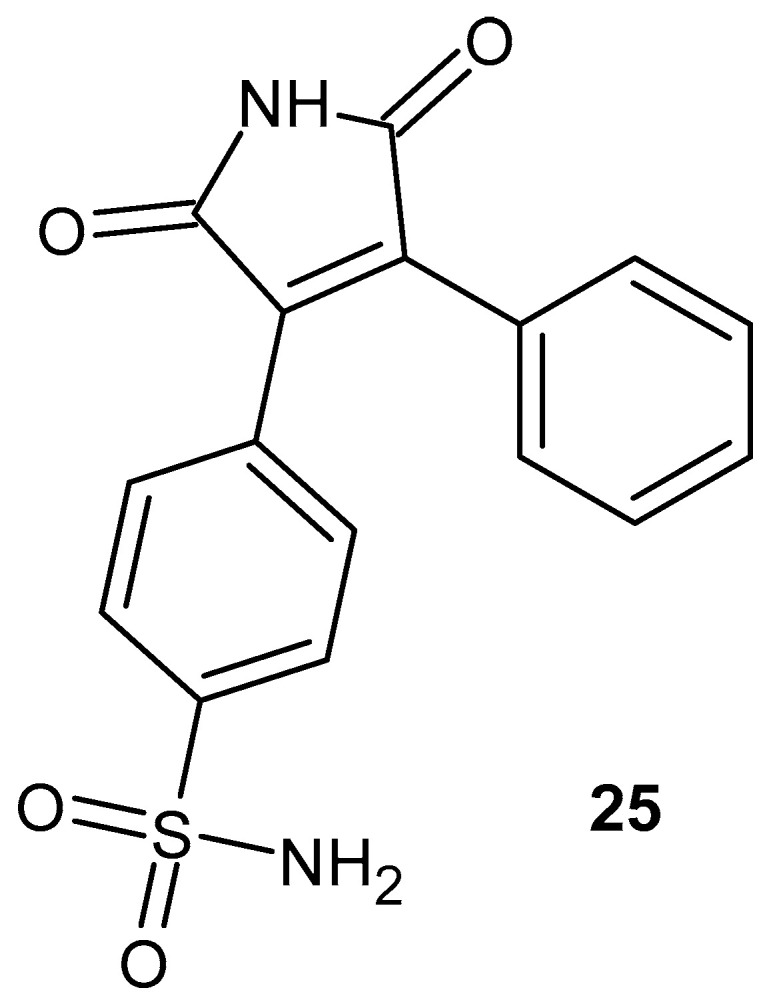
1*H*-3-(4-sulfamoylphenyl)-4-phenyl-pyrrole-2,5-dione **25** reported by Moon et al. [68].

Moon et al. [68] synthesized a series of 1*H*-pyrrole-2,5-dione derivatives bearing a benzenesulfonamide substituent. The anti-inflammatory activity of these compounds was evaluated in RAW 264.7 macrophages, assessing both cytotoxicity and the ability to inhibit lipopolysaccharide (LPS)-induced prostaglandin E_2_ (PGE_2_) production. NS-398 was used as the reference compound in the PGE_2_ inhibition assay, while Dup-697 served as the control inhibitor in the COX-2 activity assay. All tested derivatives exhibited no cytotoxicity at concentrations up to 100 µM. The most active compound in the series was 1*H*-3-(4-sulfamoylphenyl)-4-phenyl-pyrrole-2,5-dione **25** (Figure 24), which inhibited PGE_2_ production with an IC_50_ value of 0.61 µM, demonstrating markedly higher potency than the reference inhibitor NS-398.

Compound **25** was further evaluated for its inhibitory activity on PGE_2_ production induced by both peptidoglycan and poly(I:C) in RAW 264.7 macrophages, using NS-398 as a positive control [69]. The compound effectively suppressed PGE_2_ synthesis, exhibiting activity comparable to or exceeding that of classical nonsteroidal anti-inflammatory drugs (NSAIDs). Under comparative conditions, its IC_50_ was 0.61 µM, versus 0.86 µM for ibuprofen and 0.09 µM for meloxicam [69]. COX-1/COX-2 enzymatic assays revealed moderate but distinct selectivity toward the inducible isoform, with IC_50_ values of 126.78 µM for COX-1 and 9.10 µM for COX-2, corresponding to a selectivity index (SI) of 13.93. By contrast, ibuprofen exhibited markedly lower selectivity (SI= 1.15). Notably, compound **25** did not significantly affect other inflammatory markers, including nitric oxide (NO) production, inducible nitric oxide synthase (iNOS) expression, TNF-α secretion, or the mRNA levels of IL-1β and IL-6 [69].

**Figure 25:**
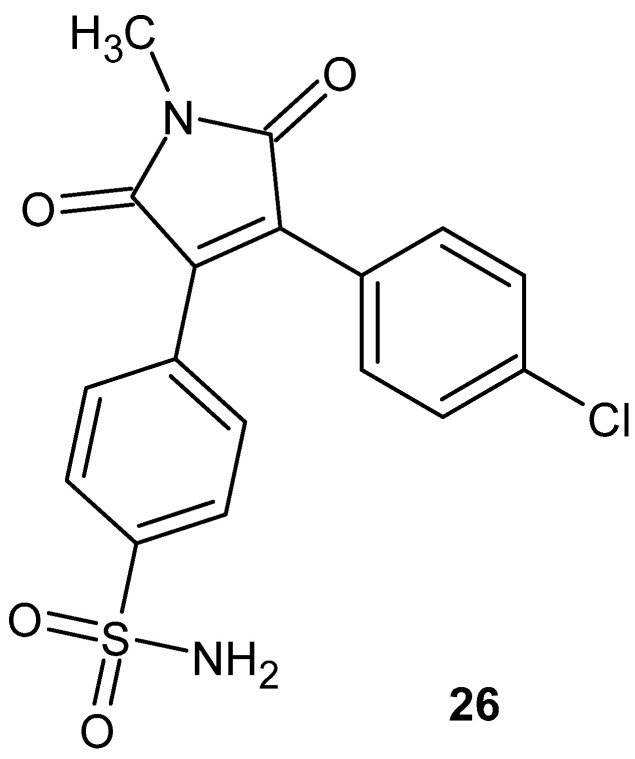
Structure of 1-methyl-1*H*-pyrrole-2,5-dione derivative MPO-0029 **26** reported by Kim et al. [70].

Kim et al. [70] synthesized a series of 1-methyl-1*H*-pyrrole-2,5-dione derivatives, designing them as potentially potent and selective cyclooxygenase-2 (COX-2) inhibitors. Their biological activity was evaluated in vitro using LPS-stimulated RAW 264.7 macrophages to induce prostaglandin E_2_ (PGE_2_) production, along with enzymatic assays assessing COX-1 and COX-2 inhibition. Within the synthesized series, the most active compound was **26**, also referred to as MPO-0029 (Figure 25).

Compound **26** exhibited strong COX-2 inhibition, with an IC_50_ value of 6.0 nM, and effectively reduced PGE_2_ production in cells (IC_50_ = 8.7 nM). The selectivity index for COX-2 exceeded SI > 168, confirming its pronounced preference for the inflammation-induced isoform while exerting minimal effects on COX-1. Compound **26** showed higher potency and selectivity toward COX-2 compared with the reference drug, celecoxib [70].

**Figure 26:**
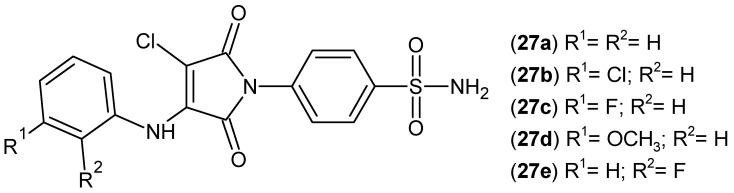
Maleimide derivatives containing a benzenesulfonamide moiety **27** were reported by Firke and Bari [71].

The synthesis of maleimide derivatives **27** incorporating a benzenesulfonamide moiety (Figure 26) has also been described by Firke and Bari [71].

The anti-inflammatory activity of these compounds was evaluated in vitro via cyclooxygenase inhibition assays (COX-1 and COX-2) and in vivo using the carrageenan-induced rat paw edema model. Most derivatives exhibited potent and selective COX-2 inhibition, with minimal effect on COX-1. Compounds **27a**–**e** demonstrated the highest biological activity, among which compound **27c** displayed the most pronounced anti-inflammatory effect, reaching maximal in vivo efficacy at 5 h, comparable to the reference drug, celecoxib (at 5 h) [71].

**Figure 27:**
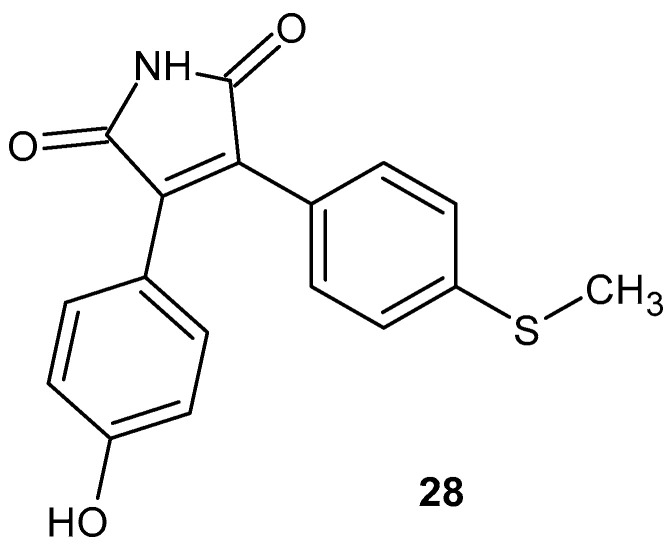
3-(4-Hydroxyphenyl)-4-(4-thiomethoxyphenyl)-1*H*-pyrrole-2,5-dione **28** reported by Shin et al. [72].

In the search for compounds with potential therapeutic applications in rheumatoid arthritis, 3-(4-hydroxyphenyl)-4-(4-thiomethoxyphenyl)-1*H*-pyrrole-2,5-dione **28** (HMP, Figure 27) was investigated [72].

In vitro studies in LPS-stimulated RAW 264.7 macrophages demonstrated that HMP **28** inhibited prostaglandin E_2_ (PGE_2_) production with an IC_50_ of 0.61 µM and selectively blocked COX-2 activity (IC_50_ = 21.01 µM) without affecting COX-1, showing no cytotoxicity at concentrations up to 100 µM. The compound also suppressed the expression of pro-inflammatory cytokines IL-1β and IL-6, as well as nitric oxide (NO) production, by downregulating the protein and mRNA levels of inducible nitric oxide synthase (iNOS) [72]. In animal models, HMP **28** reduced paw swelling in carrageenan-induced acute inflammation at oral doses of 25–50 mg/kg, concomitantly decreasing tissue PGE_2_ levels and myeloperoxidase (MPO) activity. In the adjuvant-induced arthritis (AIA) model in rats, daily oral administration of HMP (50 mg/kg) reduced paw volume by 53% and plasma PGE_2_ concentration by 42%. It significantly decreased the arthritis index, including paw edema, erythema, and joint dysfunction [72].

**Figure 28:**
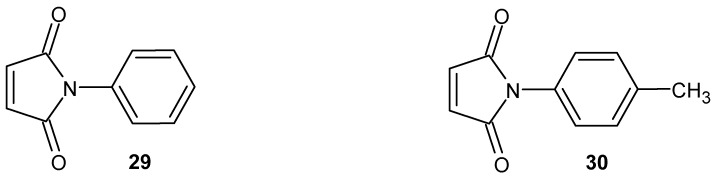
*N*-phenylmaleimides **29** and **30** reported by Noldin et al. [73].

The anti-inflammatory activity of *N*-phenylmaleimide derivatives **29** and **30** (Figure 28) was evaluated both in vitro and in vivo using myeloperoxidase (MPO) activity assays and acute inflammation models [73].

In in vitro studies, 4-methyl-*N*-phenylmaleimide (Me-NFM, **30**) (Figure 28) significantly increased MPO activity in lung homogenate supernatants as well as in purified enzyme preparations, with K_0.5_ values of 84 ± 0.3 µM and 62.85 ± 3.52 µM, respectively. In primary neutrophil cultures, a one-hour incubation with Me-NFM **30** resulted in a comparable induction of MPO activity (K = 54.47 µM), while NFM **29** (Figure 28) exhibited similar potency (K = 58.9 µM) [73]. In in vivo studies using a carrageenan-induced acute inflammation model, Me-NFM **30** enhanced leukocyte migration and MPO activity. Total leukocyte counts increased by 44–48%, neutrophils by 27–33%, and mononuclear cells by 130–148% compared with the control group. In contrast, NFM **29** did not produce significant changes in leukocyte migration or MPO activity [73].

Kalgutkar et al. [74] developed a series of *N*-substituted maleimide derivatives as potential inhibitors of prostaglandin H synthase-1 and -2 (PGHS-1 and PGHS-2). Within this series, *N*-(carboxyalkyl)maleimides displayed the most pronounced biological activity, producing rapid and concentration-dependent inactivation of cyclooxygenase function.

**Figure 29:**
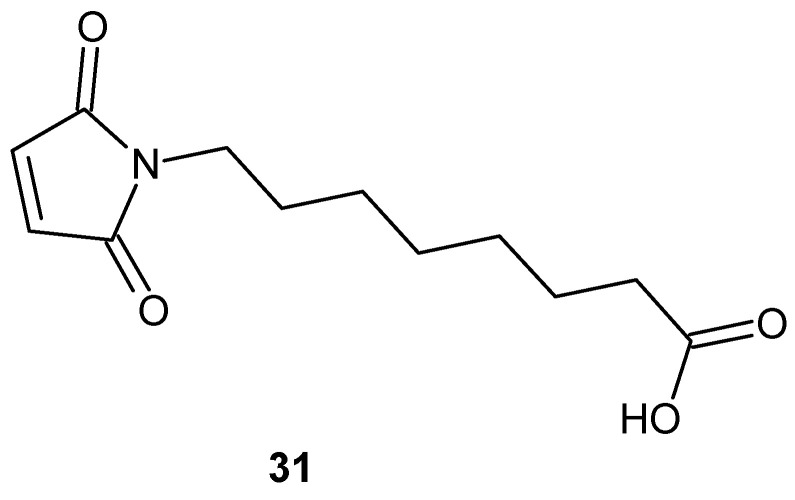
Structure of *N*-(carboxyheptyl)maleimide **31** reported by Kalgutkar et al. [74].

The most potent compound, *N*-(carboxyheptyl)maleimide **31** (Figure 29), induced rapid, stoichiometric inactivation of COX-1 activity, consistent with a non-equilibrium, covalent mechanism rather than classical reversible inhibition.

The carboxylate group was identified as a critical structural element, ensuring proper positioning of the inhibitor within the fatty acid substrate access channel and thereby enabling efficient enzyme inactivation [74].

**Figure 30:**
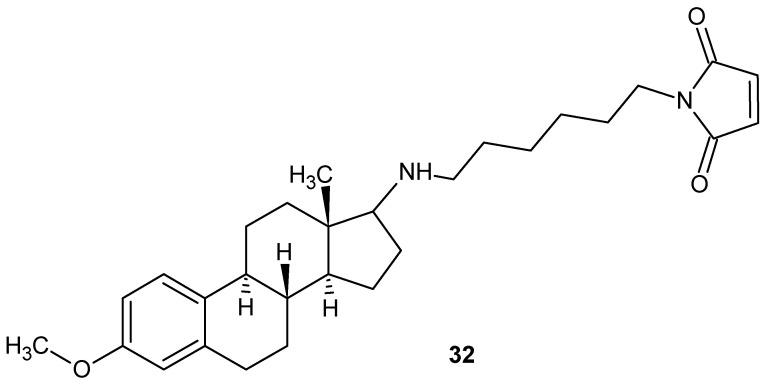
Chemical structure of U-73122 **32** reported by Hou et al. [75].

Hou et al. [75] synthesized and evaluated the activity of U73122—1-(6-((17-3-methoxyestra-1,3,5(10)-trien-17-yl)amino)hexyl)-1*H*-pyrrole-2,5-dione **32** (Figure 30), a selective phospholipase C (PLC) inhibitor.

Compound **32** demonstrated pronounced anti-inflammatory activity in vivo*,* particularly in acute inflammation models, as evidenced by reduced edema and inflammatory cell infiltration. Subsequent studies by Feißt et al. [76] identified the pyrrole-2,5-dione moiety as a key structural element mediating anti-inflammatory signaling; U73122 **32** inhibited GPCR-dependent intracellular Ca^2+^ mobilization in human polymorphonuclear leukocytes with IC_50_ values of 2–4 µM, without affecting receptor-independent calcium pathways. Moreover, the compound potently inhibited 5-lipoxygenase activity in a Ca^2+^-independent manner, with IC_50_ values of 2.4 µM in PMNL homogenates and 30 nM against recombinant human 5-LOX, indicating high affinity of the maleimide scaffold for this enzyme [76].

**Figure 31:**
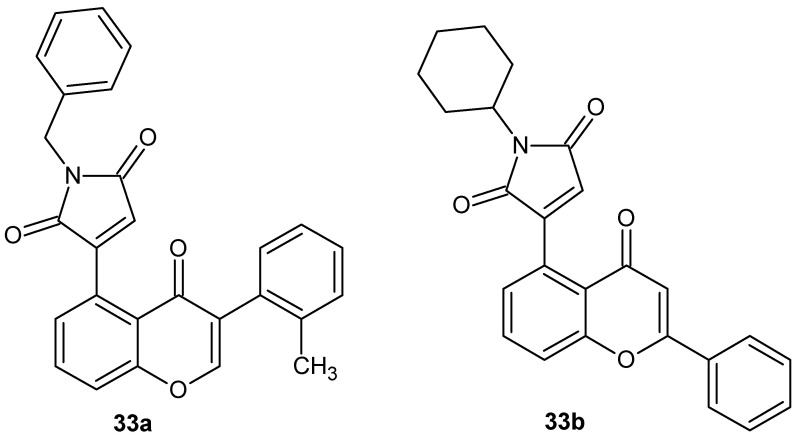
Maleimide derivatives **33a** and **33b** reported by Zhang et al. [77].

To counteract acute lung injury (ALI) associated with excessive inflammatory responses, in which increased release of pro-inflammatory cytokines such as TNF-α and IL-6 plays a central role, forty novel chromone–maleimide hybrids were designed and synthesized [77]. The majority of these hybrids exhibited pronounced anti-inflammatory activity, with the most pharmacologically active structures, **33a** and **33b**, shown in Figure 31.

Compounds **33a** and **33b** demonstrated dose-dependent inhibition of TNF-α and IL-6 release with the following IC_50_ values: **33a**: (IL-6) IC_50_ = 0.35 µM, (TNF-α) IC_50_ = 0.69 µM; **33b**: (IL-6) IC_50_ = 0.34 µM, (TNF-α) IC_50_ = 0.88 µM. In in vivo studies, both compounds significantly attenuated LPS-induced ALI, reducing inflammation and the severity of lung tissue damage. Preliminary SAR analysis indicated that the inhibitory activity of the chromone–maleimide hybrids was dependent on substitution of the chromone ring with a phenyl group at the C2 or C3 positions and the presence of a cyclohexyl or benzyl group at the nitrogen of the maleimide [77].

Paprocka et al. [78] investigated a series of 3,4-dimethyl-1*H*-pyrrole-2,5-dione derivatives, synthesized via the reaction of N^3^-substituted amidrazones with 2,3-dimethylmaleic anhydride, for their anti-inflammatory potential.

**Figure 32:**
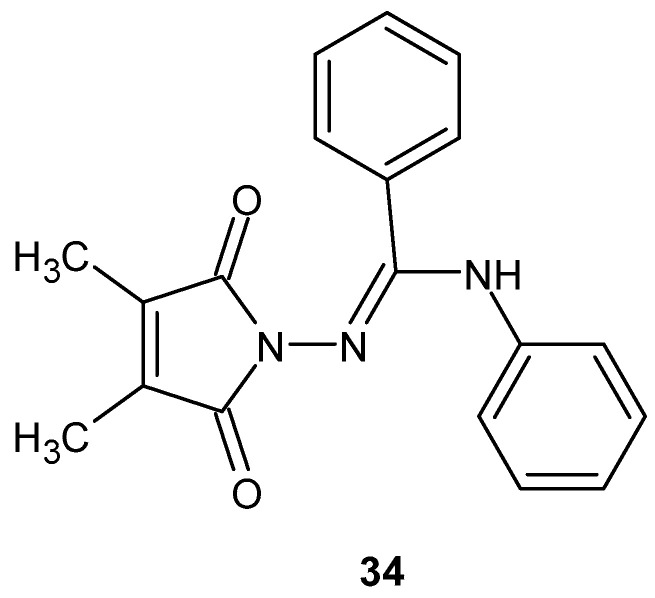
3,4-dimethyl-1*H*-pyrrole-2,5-dione derivatives **34** reported by Paprocka et al. [78].

In lipopolysaccharide (LPS)-stimulated peripheral blood mononuclear cell (PBMC) cultures, derivative **34** (Figure 32) demonstrated the most pronounced inhibitory effect, reducing IL-6 and TNF-α production only at the highest tested concentration (100 µg/mL) by 64% and 65%, respectively, substantially exceeding the effects observed for the reference drug, ibuprofen (11% and 6%, respectively) [78].

**Figure 33:**
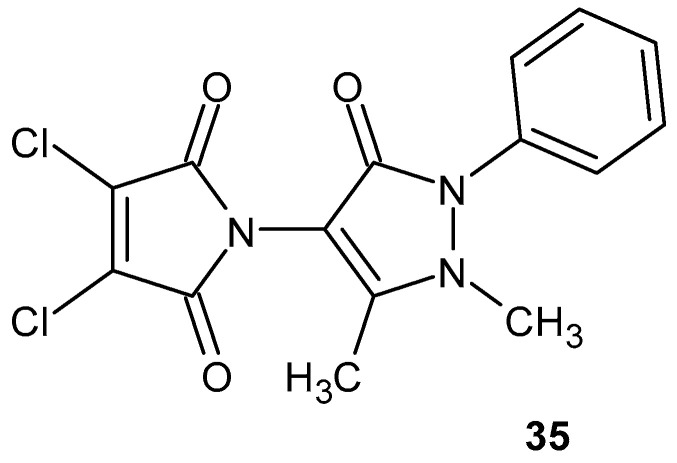
NA-3,4-DCM **35** reported by De Campos et al. [79].

De Campos et al. [79] synthesized *N*-antipyrine-3,4-dichloromaleimide (NA-3,4-DCM) **35** (Figure 33) and demonstrated its significant antinociceptive activity in models of acute pain, including the acetic acid-induced writhing test and the formalin test.

In the latter, the compound selectively inhibited the inflammatory phase, indicating a mechanism of action independent of the opioid system [79]. In subsequent studies, Quintão and co-workers [80] extended the pharmacological evaluation of NA-3,4-DCM **35** to models of chronic inflammatory and neuropathic pain. The compound significantly reduced carrageenan-induced mechanical hypernociception by 61% ± 8%. In the formalin test, systemic administration of NA-3,4-DCM **35** selectively inhibited the inflammatory phase (ID_50_ = 16.2 µmol/kg), whereas local, intrathecal, and supraspinal administration resulted in inhibition of both phases of nociceptive behavior. Mechanistic investigations suggested the involvement of glutamatergic pathways, as the compound inhibited nociceptive responses induced by glutamate, while not affecting responses mediated by AMPA or substance P [80].

**Figure 34:**
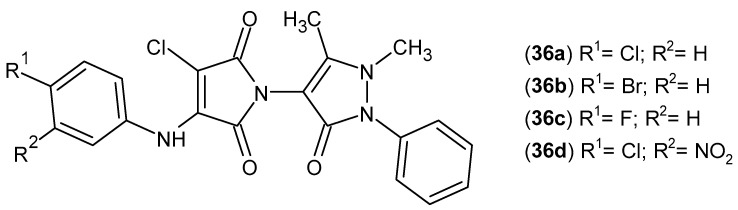
*N*-antipyrine-3,4-dichloromaleimides **36a**–**d** reported by Mahle et al. [81].

Mahle et al. [81] synthesized new cyclic imide derivatives via the reaction of *N*-antipyrine-3,4-dichloromaleimides **36** (Figure 34) with various aromatic amines and evaluated their analgesic and anti-inflammatory activities. All compounds were initially tested in the acetic acid-induced writhing model in mice. Most derivatives, particularly aniline and halogenated compounds with para-substitution, **36a**–**d**, exhibited strong analgesic effects, being 33–284 times more potent than the reference drugs, ASA (acetylsalicylic acid) and MET (metamizole).

The most active derivatives were subsequently evaluated in the formalin-induced pain model. In this assay, compounds **36a**, **36c**, and **36d** exhibited significant inhibition of the second (inflammatory) phase of nociception, with compound **36d** demonstrating approximately six-fold greater anti-inflammatory activity compared with acetylsalicylic acid (ASA). These findings indicate that para-positioned electron-withdrawing substituents markedly enhance both analgesic and anti-inflammatory activities [81].

**Figure 35:**
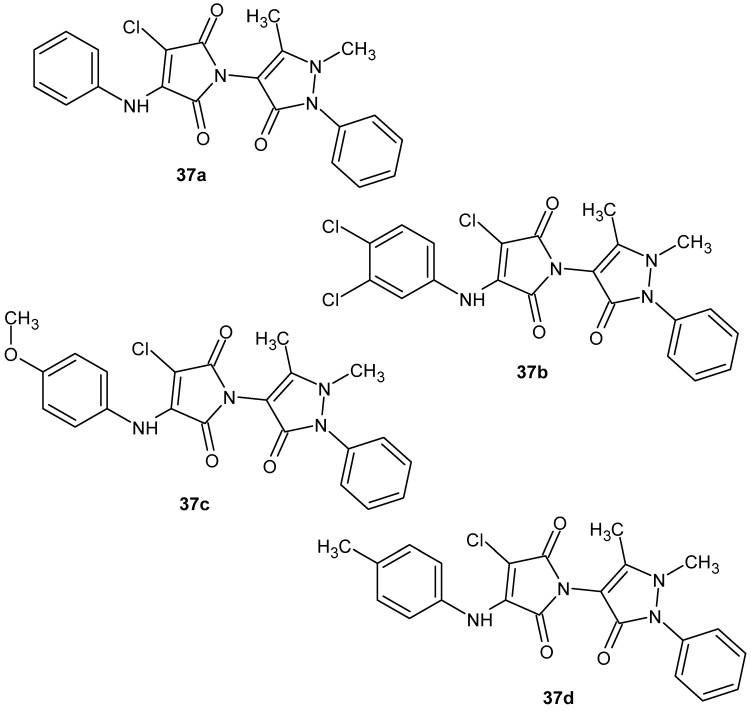
*N*-antipyrine–3,4-dichloromaleimide derivatives **37a**–**d** reported by Fratoni et al. [82].

Based on previous reports on the pharmacological activity of *N*-antipyrine-3,4-dichloromaleimide derivatives [81], Fratoni et al. [82] conducted an in-depth evaluation of their anti-inflammatory potential in the RAW 264.7 murine macrophage cell line. In the first stage, six selected derivatives (**35**, **36a**, and **37a**–**d**; Figure 35) were assessed for their ability to inhibit lipopolysaccharide (LPS)-induced nitric oxide (NO) production as an early marker of anti-inflammatory activity.

Among the compounds tested, the most pronounced NO inhibition was observed for **35**, **36a,** and **37c**. Therefore, further studies focused on these three derivatives. Compounds **35**, **36a**, and **37c** were then evaluated for their effects on cytokine profiles in LPS-stimulated macrophages. All three compounds reduced the levels of the pro-inflammatory cytokines IL-1β and TNF-α. Additionally, compounds **35** and **36a** inhibited the production of IL-6 and MCP-1, while none of the tested compounds affected IFN-γ levels. Moreover, compounds **35** and **36a** significantly inhibited phosphorylation of the p65 subunit of NF-κB. Importantly, these derivatives not only suppressed the pro-inflammatory response but also promoted an anti-inflammatory profile, stimulating the secretion of IL-4 (**35**) and IL-13 (**36a**) [82].

**Figure 36:**
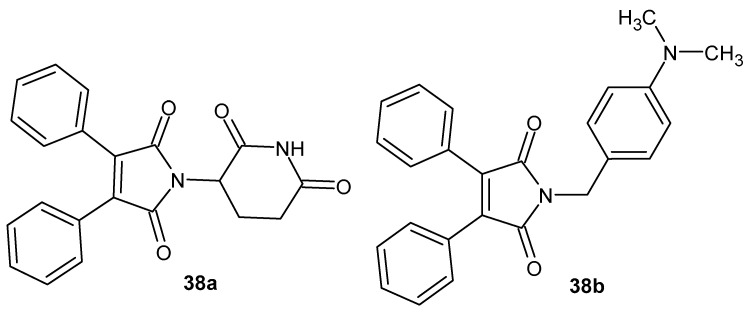
Structure of maleimide derivatives **38a** and **38b** reported by Jung et al. [83].

Jung et al. [83] synthesized a series of twenty thalidomide analogues in which the benzene rings of the phthalimide moiety were replaced with two separate diphenyl rings within the maleimide ring, and the *N*-glutarimide moiety was substituted with various phenyl groups (Figure 36). These structural modifications were aimed at enhancing the anti-inflammatory activity of thalidomide, specifically in the context of inhibiting nitric oxide (NO) production in lipopolysaccharide (LPS)-stimulated BV2 microglial cells.

Among the synthesized compounds, the dimethylaminophenyl-substituted analogue **38b** exhibited the highest pharmacological activity, with an IC_50_ of 7.1 μM, representing a significant improvement compared to the glutarimide reference analogue **38a** (IC_50_ > 50 μM) [83].

Compound **38b** inhibited NO production in a dose-dependent manner without inducing cytotoxicity. Furthermore, it suppressed the release of pro-inflammatory cytokines as well as the expression of key inflammatory enzymes, including inducible nitric oxide synthase (iNOS) and cyclooxygenase-2 (COX-2). The authors suggested that the mechanism of action of compound **38b** involves the inhibition of the NF-κB and p38 MAPK signaling pathways, which play critical roles in the initiation of inflammatory responses [83].

Derivatives of 1*H*-pyrrole-2,5-dione were investigated as potential inhibitors of cholesterol absorption. In vitro studies were performed using the human embryonic kidney cell line HEK293 and the murine macrophage cell line RAW 264.7 [84]. The biological activity of the synthesized compounds was evaluated using biochemical assays measuring cholesterol uptake, together with cytotoxicity assays to assess cellular safety.

**Figure 37:**
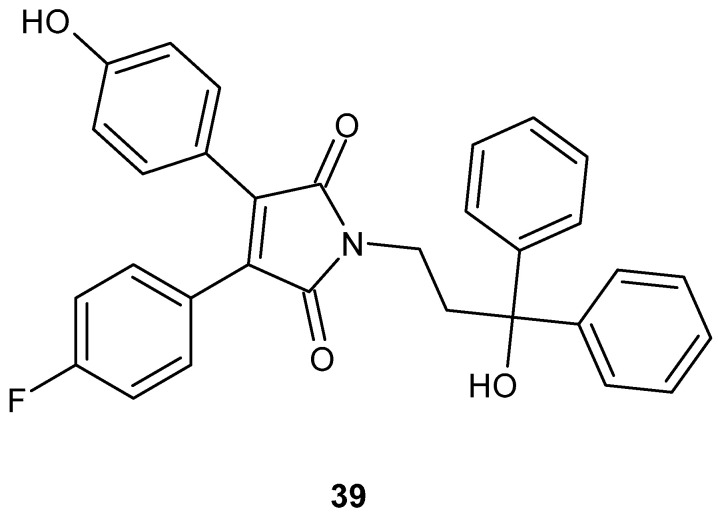
Structure of compound **39** reported by Yuan et al. [84].

Among all the synthesized derivatives, compound **39** (Figure 37) exhibited the highest inhibitory activity against cholesterol absorption in vitro, surpassing the reference drug, ezetimibe.

Compound **39** showed no significant cytotoxicity toward HEK293 and RAW 264.7 cells (LC_50_ > 100 μM for both cell lines). Further studies demonstrated that compound **39** effectively suppressed lipid accumulation in macrophages. It induced a dose-dependent reduction in the release of lactate dehydrogenase (LDH) and tumor necrosis factor-α (TNF-α), as well as a decrease in the generation of reactive oxygen species (ROS). Collectively, these results indicate that compound **39** may effectively inhibit foam cell formation and the associated inflammatory response, highlighting its potential relevance in the context of atherosclerosis and related metabolic disorders [84].

**Figure 38:**
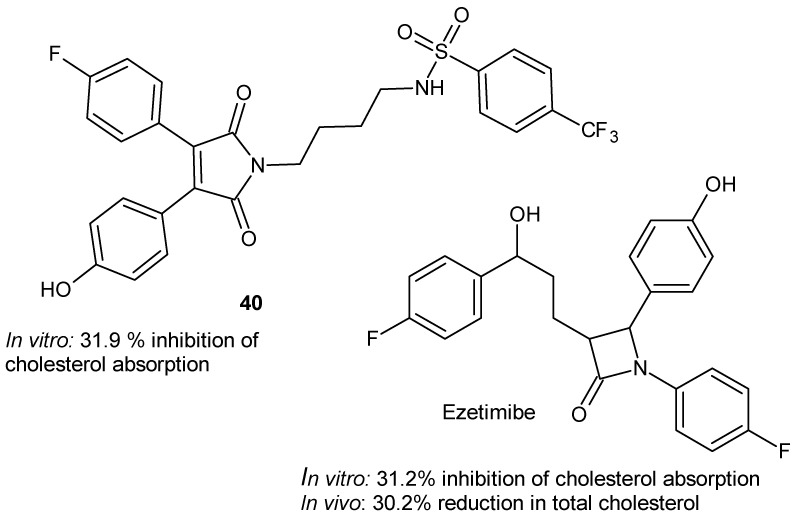
Structure and pharmacological activity of compound **40** and ezetimibe reported by Yuan et al. [85].

Cholesterol absorption inhibitors (CAIs) targeting the Niemann–Pick C1-like 1 (NPC1L1) protein have been proposed as a novel strategy for the treatment of hyperlipidemia, with ezetimibe being the only CAI currently available on the market. To develop new CAIs with improved therapeutic efficacy, Yuan et al. [85] synthesized thirteen 1*H*-pyrrole-2,5-dione derivatives bearing a sulfonamide group in the side chain. The pharmacological activity of the most potent maleimide derivative, **40**, was comparable to that of ezetimibe (Figure 38).

These results suggest that the 1*H*-pyrrole-2,5-dione ring can effectively serve as a replacement for 2-azetidinone, leading to the development of pharmacologically active structures [85].

**Figure 39:**
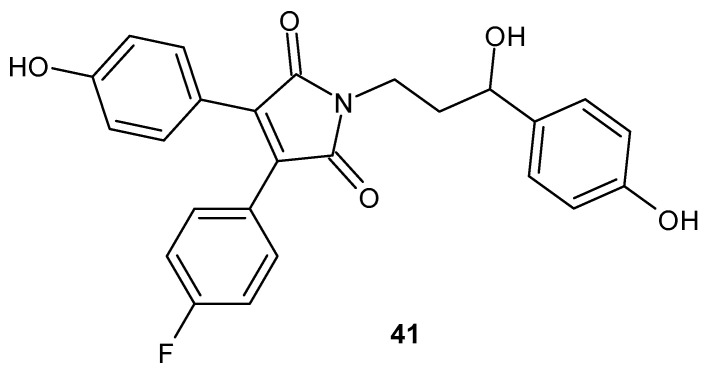
3-(4-Fluorophenyl)-1-[(3S)-3-hydroxy-3-(4-hydroxyphenyl)propyl]-4-(4-hydroxyphenyl)-1*H*-pyrrole-2,5-dione **41** reported by Xia et al. [86].

Continuing the research on maleimide derivatives affecting cholesterol reduction, Xia et al. [86] synthesized seven 2-azetidinone derivatives and eighteen 1*H*-pyrrole-2,5-dione derivatives, most of which significantly inhibited cholesterol uptake in vitro. Moreover, one of the most active inhibitors, 3-(4-fluorophenyl)-1-[(3S)-3-hydroxy-3-(4-hydroxyphenyl)propyl]-4-(4-hydroxyphenyl)-1*H*-pyrrole-2,5-dione **41** (Figure 39), exhibited no cytotoxicity in L02 and HEK293T cell lines.

Further analyses demonstrated that compound **41** markedly reduced levels of TNF-α, ROS, MDA, and LDH in vitro, indicating its potential as a novel cholesterol absorption inhibitor [86].

**Figure 40:**
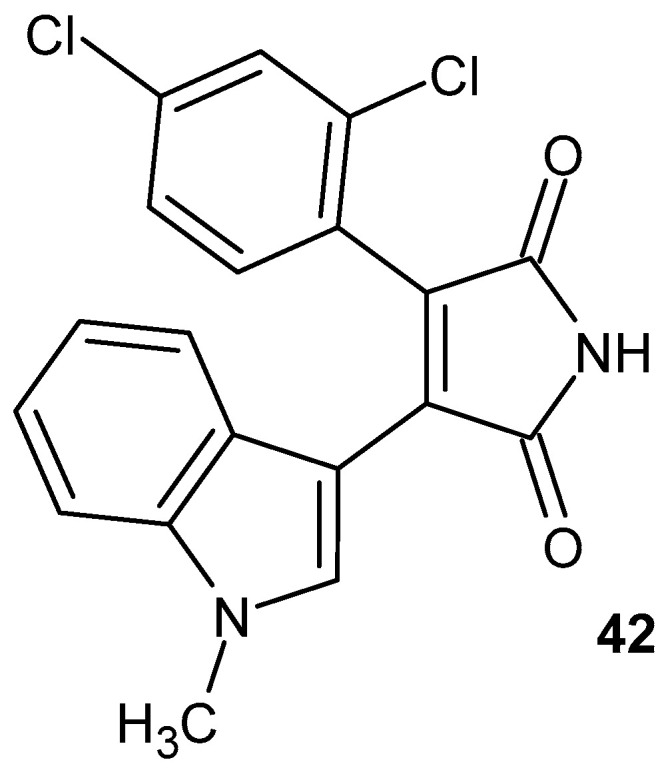
Maleimide derivative SB216763 **42** reported by Saleh et al. [87].

Inflammation, a major contributing factor to a variety of diseases, including neurodegenerative disorders (e.g., Alzheimer’s disease), metabolic dysfunctions, and certain malignancies, is closely associated with excessive GSK-3β activity. Selective inhibitors of this kinase have emerged as promising modulators of the inflammatory response, capable of attenuating the production of pro-inflammatory cytokines and mitigating oxidative stress. Among these, the maleimide derivative SB216763 **42** (Figure 40) is a potent inhibitor of both GSK-3α and GSK-3β, with an IC_50_ of 34.3 nM for GSK-3β. SB216763 acts as an ATP-competitive inhibitor, effectively suppressing the enzymatic activity of GSK-3 isozymes. Beyond its anti-inflammatory potential, SB216763 **42** also exhibits neuroprotective properties, preventing neuronal cell death mediated via the PI3-kinase signaling pathway [87].

**Figure 41:**
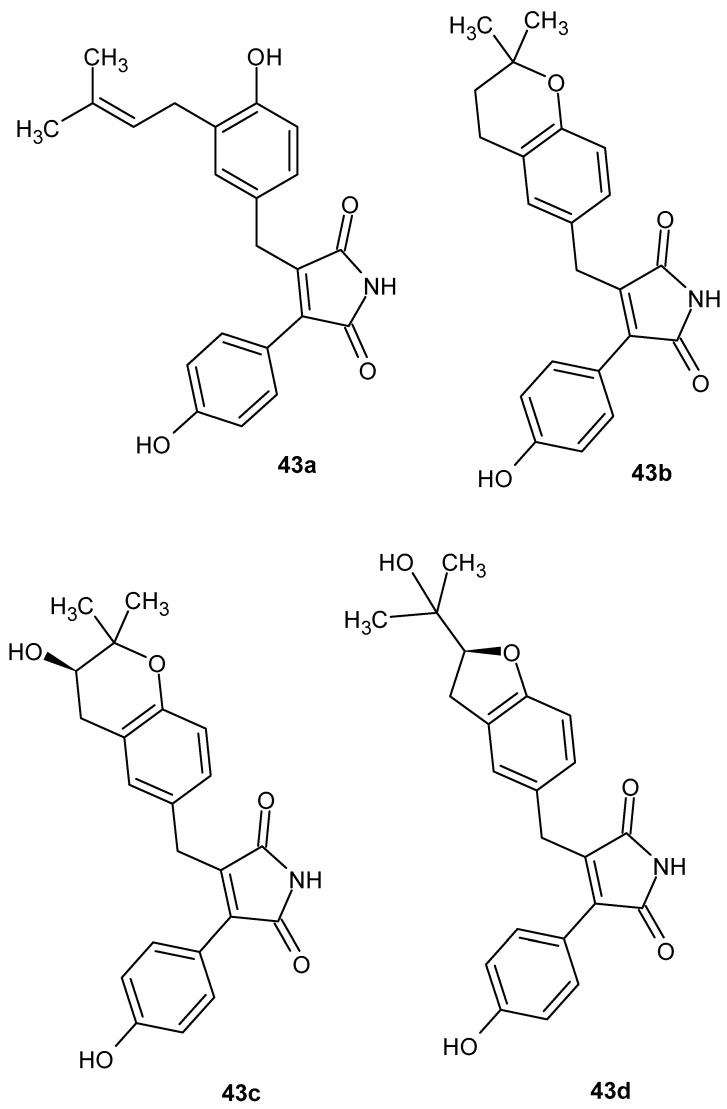
Structures of asperimides A-D **43a**–**d** reported by Liao et al. [88].

The anti-inflammatory activity of maleimide derivatives has also been investigated among natural products, in which the maleimide ring constitutes a key structural feature of newly isolated butenolides from *Aspergillus terreus* SC1550. In this study, four new aromatic butenolides—asperimides A-D **43a**–**d** (Figure 41)—were isolated [88].

Biological activity was evaluated using an LPS-stimulated RAW 264.7 macrophage model by assessing the inhibition of nitric oxide production using the Griess assay. Asperimides **43c** and **43d**, both containing a 1*H*-pyrrole-2,5-dione core, exhibited the most pronounced anti-inflammatory effects, with IC_50_ values of 0.78 ± 0.06 μM and 1.26 ± 0.11 μM, respectively, markedly surpassing the activity of indomethacin (IC_50_ = 37.5 ± 1.6 μM). Asperimides **43a** and **43b** were less active. The MTT assay revealed no significant cytotoxicity of the tested compounds at their effective concentrations. Collectively, these results unequivocally confirm the crucial role of the maleimide ring in the anti-inflammatory activity of the isolated structures [88].

**Figure 42:**
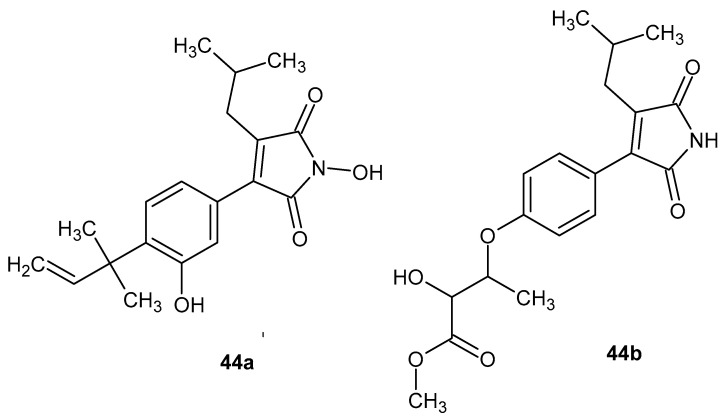
Maleimide derivatives **44a** and **44b** were isolated from the mycelium of the fungus *Antrodia cinnamomea* [90].

Another example of naturally derived compounds is maleimide derivatives isolated from the mycelium of the fungus *Antrodia cinnamomea* [89]. Among the identified metabolites by Wu et al. [90], antrocinnamomin F **44a** and antrocinnamomin H **44b** exhibited the highest anti-inflammatory activity (Figure 42)Their biological activity was evaluated in an in vitro model based on the inhibition of lipopolysaccharide (LPS)-induced nitric oxide (NO) production in RAW 264.7 murine macrophages. Antrocinnamomin F **44a** and antrocinnamomin H **44b** inhibited NO production with IC_50_ values of 30.1 ± 0.3 µM and 29.9 ± 1.5 µM, respectively, demonstrating potent anti-inflammatory activity comparable to or stronger than that of quercetin, used as a positive control [90].

**Figure 43:**
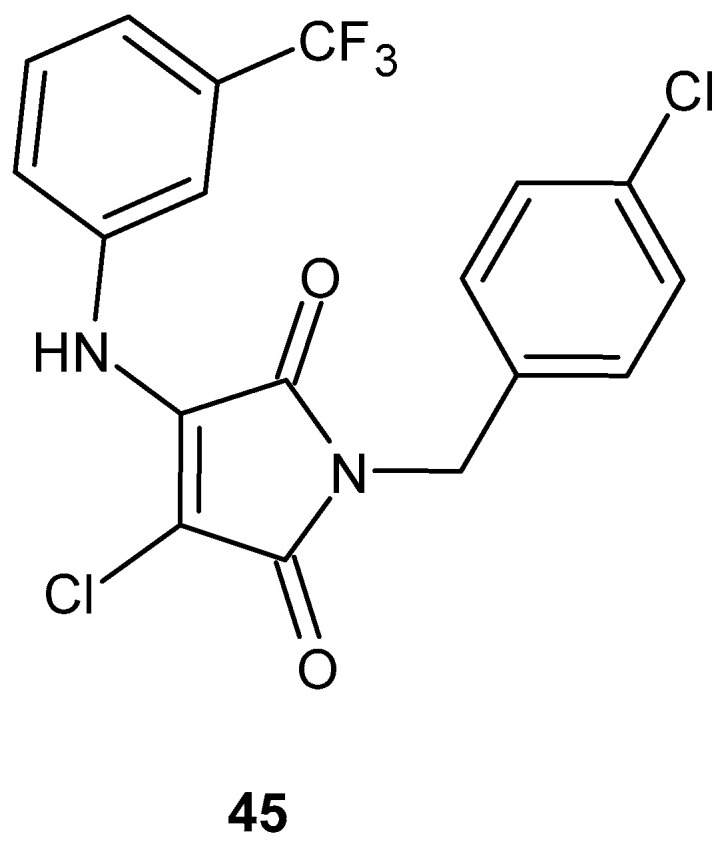
1-(4-chlorobenzyl)-3-chloro-4-(trifluoromethylphenylamino)-1*H*-pyrrol-2,5-dione (MI-1) **45** reported by Kuznietsova et al. [91].

Kuznietsova et al. [91,92] conducted a series of studies investigating the anti-inflammatory effects of synthetic pyrrole derivatives, including 1-(4-chlorobenzyl)-3-chloro-4-(trifluoromethylphenylamino)-1*H*-pyrrol-2,5-dione (MI-1) **45** (Figure 43), in various rat models of inflammation.

In their preliminary study, Kuznietsova et al. [91] investigated the anti-inflammatory potential of the multikinase inhibitor MI-1 **45** in experimental models of α-naphthylisothiocyanate-induced acute (3 days) and chronic (28 days) cholangitis in rats. Histopathological evaluation demonstrated that MI-1 markedly alleviated hepatic injury, fibrosis, and inflammatory infiltration, with reductions ranging from 46% to 86% relative to control groups. These tissue-level improvements were accompanied by normalization of serum biochemical parameters and leukocyte profiles, indicating attenuation of the systemic inflammatory response. Notably, the therapeutic effects persisted after a 28-day recovery period without further administration of the compound. Complementary in vitro assays revealed that MI-1 reduced the viability of HL60, HepG2, and human peripheral blood lymphocytes, with IC_50_ values of 0.6, 9.5, and 8.3 µg/mL, respectively, while normal NIH3T3 fibroblasts remained largely unaffected [91].

In a subsequent study, Kuznietsova et al. [92] investigated (MI-1) **45** in a rat model of chronic ulcerative colitis (UC). Oral administration of (MI-1) **45** for 14 days substantially improved colonic mucosal integrity, lowering the gastrointestinal tissue index (GTI) to nearly control levels. Additionally, MI-1 **45** modulated oxidative stress markers, decreasing malondialdehyde (MDA) levels, normalizing protein carbonyl content, and restoring superoxide dismutase (SOD) activity. Comparative analysis demonstrated that MI-1 **45** was more effective than prednisolone in reducing tissue damage [92].

Kotlyar et al. [93] demonstrated that MI-1 **45** markedly reduced oxidative stress and intestinal damage in a rat model of acetic acid-induced chronic ulcerative colitis, showing effects comparable to prednisolone.

**Figure 44:**
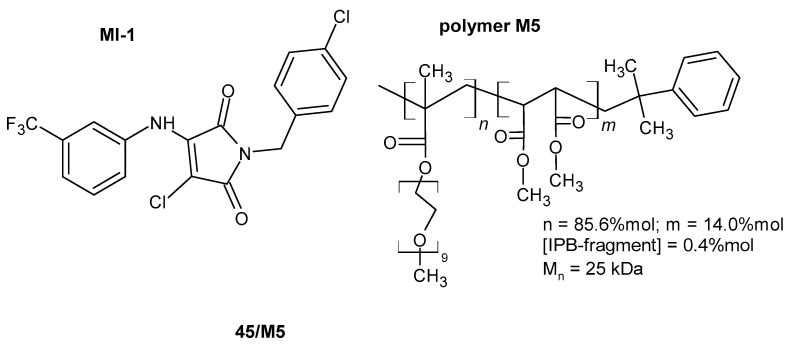
Structure of complex **45**/**M5** reported by Kotlyar et al. [93].

In further studies, the multikinase inhibitor MI-1 **45** was immobilized on a poly(PEGMA-co-DMM) polymeric carrier to improve its water solubility and biological performance. The **45/M5** complex (Figure 44) was evaluated using in vitro cell viability, clonogenic, apoptosis, DNA damage, and cell cycle assays, demonstrating preserved or enhanced activity with high selectivity toward transformed cells.

The observed effects on apoptosis induction and cell cycle regulation support the involvement of MI-1 **45** in pathways relevant to inflammation-associated hyperproliferative processes [94].

**Figure 45:**
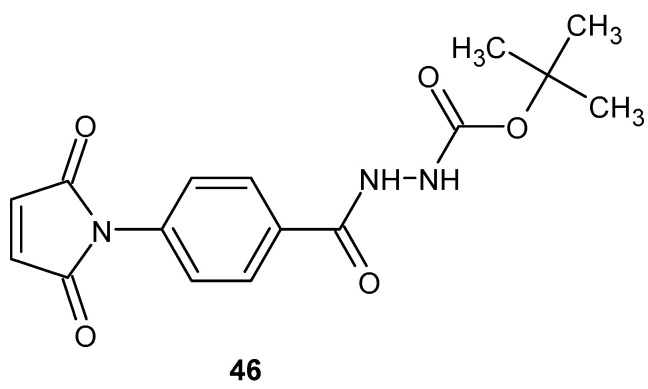
Structure of maleimide derivative **46** (DAB-1) reported by Hamelin-Morrissette et al. [95].

Inflammation is a critical factor driving cancer progression, including bladder cancer, highlighting the need for compounds with both anti-inflammatory and anticancer properties. Hamelin-Morrissette et al. [95] identified a novel compound, designated as compound **46**, a maleimide derivative (Figure 45), which exhibits anti-inflammatory activity and potential anticancer effects.

Compound **46** was subjected to detailed in vitro evaluation in human macrophages (hMϕs) and macrophage-like J774A.1 cells. In vitro studies demonstrated that compound 46 at a concentration of 10 µM significantly inhibited the activation of STAT1 and STAT3 signaling pathways in cells stimulated with IFN-γ and IL-6, reducing their activity by 38% and 64%, respectively. Following a 3 h pre-treatment in IFN-γ-stimulated cells, the expression of pro-inflammatory markers was markedly suppressed, with CD40 decreased by 87% and MHC II by 49%. In macrophage migration assays, compound 46 reduced IL-6-induced cell motility by 92%. Moreover, in J774A.1 cells stimulated with IFN-γ and TNF-α, treatment with 25 µM of compound **46** decreased nitric oxide (NO) production by approximately 91%, as measured by the Griess assay [95].

**Figure 46:**
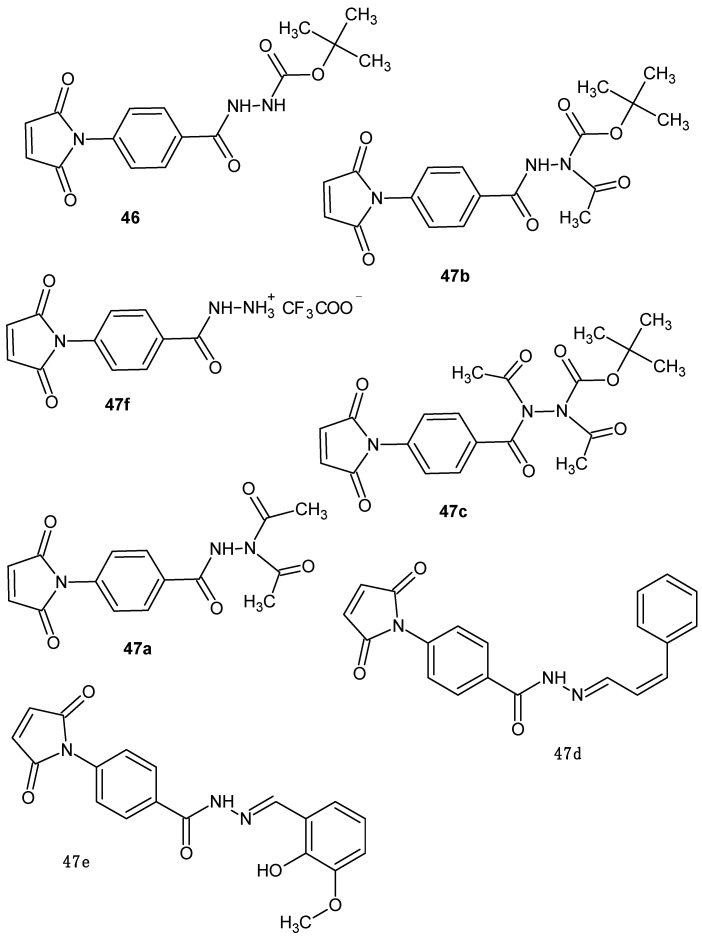
Structure of DAB-1 **46** and its derivatives **47a**–**f** reported by Oufqir et al. [96].

Oufqir et al. [96] synthesized a new generation of maleimide derivatives aimed at developing compounds with enhanced anti-inflammatory and anticancer properties, potentially applicable for the treatment of bladder cancer. The starting point of this research was the earlier synthesis of DAB-1 **46**, which contains maleimide and hydrazide moieties and exhibited promising biological activity; however, its activity profile and cytotoxicity were not optimal [96]. To improve the structure of DAB-1 **46**, the authors designed a series of five DAB-1 analogues, including DAB-2-28 **47a**, DAB-2-31A **47b**, and DAB-2-31B **47c**, and two third-generation compounds, DAB-3-27 **47d** and DAB-3-33 **47e**, primarily modifying the hydrazide portion of the molecule to enhance its pharmacological properties (Figure 46).

All five molecules were evaluated in vitro for their anti-inflammatory and antiproliferative activities. In vitro, ex vivo, and in vivo studies demonstrated that DAB-2-28 **47a** exhibited lower cytotoxicity compared with DAB-1 **46**, while more effectively inhibiting nitric oxide (NO) production induced by pro-inflammatory cytokines (IFN-γ and TNF-α), as well as more efficiently blocking the IL-6/STAT3 and TNF-α/NF-κB signaling pathways, which play crucial roles in inflammatory processes and tumor progression. In peritoneal macrophages stimulated with IFN-γ and LPS, DAB-2-28 **47a** effectively suppressed the induction of the pro-inflammatory enzymes iNOS and COX-2 [93].

The compound DAB-2-28 **47a** was further investigated by Fortin et al. [97] in 2025 in MCF-7 and MDA-MB-231 breast cancer cells. The compound demonstrated strong anti-inflammatory activity, reducing cell migration and invasion, MMP9 expression, and gelatinase activity induced by macrophage-derived factors. Moreover, DAB-2-28 **47a** inhibited the phosphorylation of key pro-EMT transcription factors, including NF-κB, STAT3, SMAD2, CREB, and AKT, suggesting that its anti-inflammatory effect contributes to the suppression of EMT processes in breast cancer cells [97].

**Figure 47:**
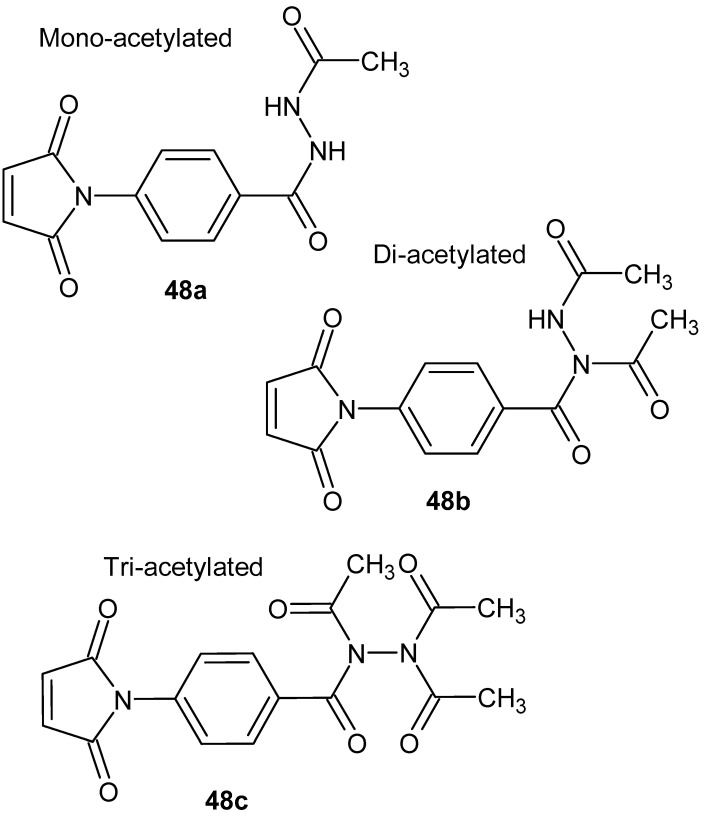
Derivatives maleimide with varied acylation patterns on the hydrazide core **48a**–**c** reported by Cloutier et al. [98].

The high anti-inflammatory activity of DAB-1 **46** served as an inspiration for further structural modifications. Cloutier et al. [98] designed new derivatives with varied acylation patterns on the hydrazide core. They obtained a monoacetylated product, **48a**; a diacetylated regioisomer, **48b**; and a triacetylated compound, **48c** (Figure 47).

Additionally, three higher homologs, both monoacylated and diacylated, were prepared using appropriate anhydrides. All monoacylated hydrazide derivatives were effective in inhibiting nitric oxide (NO) production, as measured by the Griess assay. Using the MTT assay, it was observed that these same compounds exhibited slightly lower toxicity (average cell viability: ~90%) in murine bladder cancer MB49-I cells compared to the reference DAB-1 **46** molecule (85%). The most active monoacetylated derivative, 1, demonstrated approximately 83% inhibition of NO production relative to the number of viable/proliferating cells [98].

**Figure 48:**
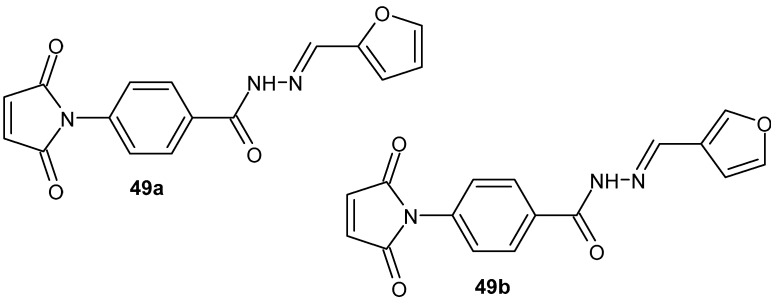
DAB-1 hydrazide derivatives **49a** and **49b** reported by Cloutier et al. [99].

Continuing their investigations, Cloutier et al. [99] synthesized a series of DAB-1 **46** hydrazide derivatives. The anti-inflammatory activity of these compounds was evaluated both in vitro and in vivo. Most of the new compounds were found to be essentially non-toxic to RAW 264.7 cells. Their anti-inflammatory potential was assessed by measuring the effect on nitric oxide production in cells using the Griess assay. Some of the derivatives significantly inhibited nitric oxide production, proving to be substantially more effective than the parent compound, DAB-1 **46**. The most active compound of the series was derivative **47e** (Figure 46), which was four times more potent than DAB-1 **46**. In addition to the compounds 47e, furan-containing derivatives **49a** and **49b** (Figure 48) merit attention, as they exhibited normalized NO inhibition of 71% and 75%, respectively, without detectable cytotoxicity toward RAW 264.7 cells, indicating pronounced anti-inflammatory potential [97].

In vivo studies in models of acute inflammation and invasive bladder cancer tumors demonstrated that derivative **49** reduced inflammation in mice, exhibited comparable anti-inflammatory activity, and displayed higher anti-tumor activity compared to DAB-1 **46**, without apparent signs of toxicity [99].

**Figure 49:**
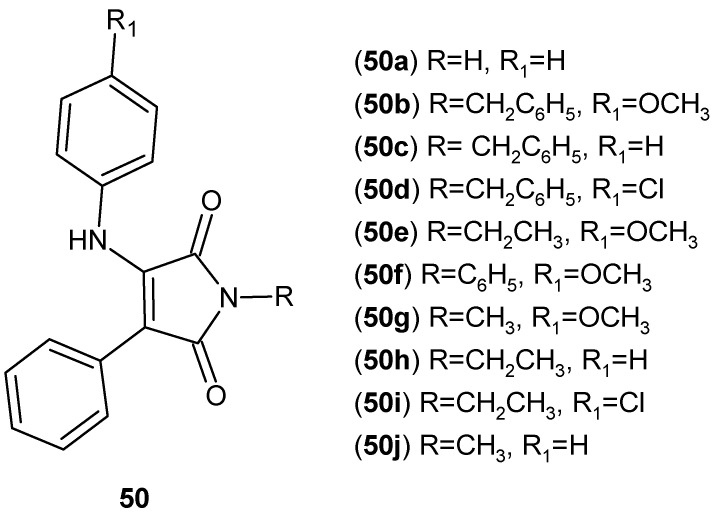
Structure of maleimide derivatives **50a**–**j** reported by Jaye et al. [100].

Jaye et al. [100] reported the synthesis of a series of substituted maleimide derivatives **50a**–**j** (Figure 49) and their evaluation as agonists of liver X receptors (LXRα and LXRβ).

The compounds **50a**–**j** were assessed in vitro using coactivator recruitment and LXR reporter assays, revealing high agonistic potency with EC_50_ values ranging from 40 to 150 nM. In cellular models, LXR activation by selected maleimide derivatives resulted in suppression of pro-inflammatory gene expression, including TNF-α, IL-6, and COX-2, indicating their anti-inflammatory potential mediated via LXR signaling [100].

### 2.2. Bicyclic Derivatives

#### 2.2.1. Bicyclic Imides Fused to a Benzene Ring

Phthalimide is an organic chemical compound belonging to the acid imide group. It is a derivative of phthalic acid, in which two carbonyl groups are bonded to a single nitrogen atom, forming a characteristic imide ring. It is also used in the pharmaceutical and chemical industries, as well as in the synthesis of biologically active compounds.

**Figure 50:**
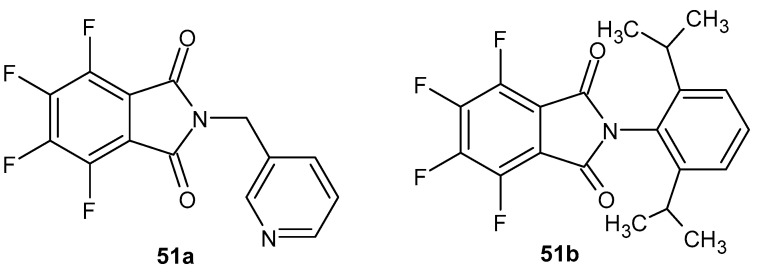
Tetrafluorophthalimide derivatives **51a**–**b** reported by Colina et al. [101].

Tetrafluorophthalimide derivatives **51a**–**b** (Figure 50) were synthesized by Colina et al. [101]. *N*-(pyridin-3-ylmethyl)-4,5,6,7-tetrafluorophthalimide **51a** was the most potent compound in the series tested, demonstrating slightly greater (84%) inhibition of TNF-α production than 2-[2,6-di(propan-2-yl)phenyl]-4,5,6,7-tetrafluoro-1*H*-isoindole-1,3(2*H*)-dione **51b** (73%) and proved highly effective in reducing ear thickness in mice following topical administration at a dose of 2 × 500 µg, achieving 79% inhibition of the inflammatory process. Although its effect was weaker than that of dexamethasone, this compound remained significantly active even at the lower dose of 2 × 100 µg; the toxicity of compound **51a** was LD_50_ = 1.05 ± 0.27 mM kg^−1^. Evaluation of the ID_50_ value in the carrageenan-induced rat paw edema model following oral administration showed that derivative **51a** had an ID_50_ = 0.14 µM·kg^−1^ and a potency similar to that of compound **51b** (ID_50_ = 0.15 µM·kg^−1^).

**Figure 51:**
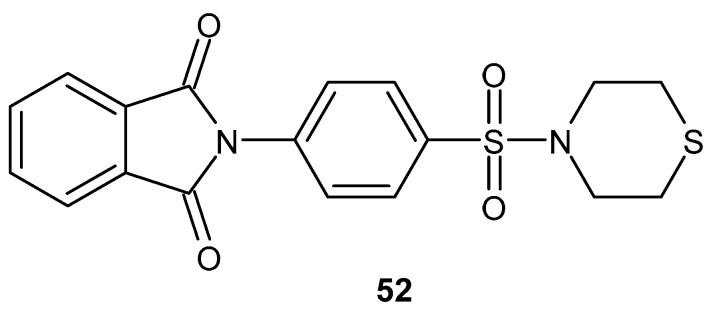
2-[4-(1,4-Thiazinan-4-ylsulfonyl)phenyl]-1,3-isoindoline dione **52** reported by Lima et al. [102].

The synthesis and activity of compounds containing a sulfonylthiomorpholine were described by Lima et al. [102]. 2-[4-(1,4-Thiazinan-4-ylsulfonyl)phenyl]-1,3-isoindoline dione **52** (Figure 51) demonstrated significant anti-inflammatory activity, strongly limiting LPS-induced neutrophil recruitment at ED_50_ = 2.5 mg·kg^−1^. The inhibitory capacity of the tested compound, **52**, was 72 ± 7.2%, while for the reference drug, thalidomide, it was 50 ± 8.6%. The derivative **52** also inhibited TNF-α production.

**Figure 52:**
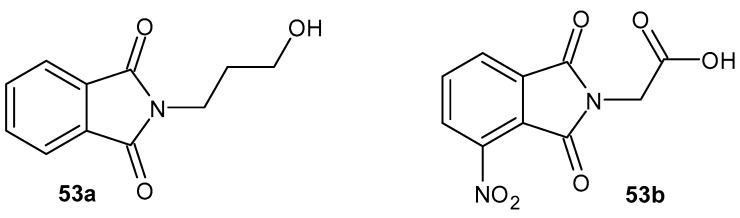
*N*-alkilophthalimide derivatives **53a**–**b** reported by Batista et al. [103].

Batista et al. [103] analyzed the effects of five phthalimide analogues lacking the glutarimide moiety in experimental models of acute and chronic inflammatory neuropathic pain. Among the substances evaluated, two compounds (Figure 52), *N*-3-hydroxypropylphthalimide **53a** (546 mg/kg) and *N*-carboxymethyl-3-nitrophthalimide **53b** (700 mg/kg), showed analgesic activity in various models, including chronic inflammatory and neuropathic pain models.

**Figure 53:**
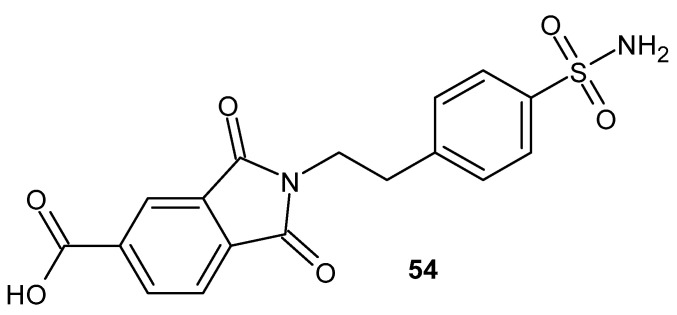
1,3-Dioxo-2-(4-sulfamoylphenethyl)isoindole-5-carboxylic acid **54** reported by Abdel-Azis et al. [104].

Abdel-Azis et al. [104] presented the synthesis of phthalimide derivatives and conducted studies on their anti-inflammatory, ulcerogenic, and cytotoxic effects in vitro. 1,3-Dioxo-2-(4-sulfamoylphenethyl)isoindole-5-carboxylic acid **53** (Figure 53) was found to be the most potent COX-2 inhibitor (IC_50_ = 0.10 µM), characterized by optimal selectivity (COX-1 IC_50_ = 49.0 µM) and comparable to celecoxib (COX-1, IC_50_ > 50 µM; COX-2, IC_50_ = 0.12 µM). The derivative **54** reduced edema by 86.6% in an in vivo anti-inflammatory activity study in a conventional carrageenan-induced paw edema model in rats, while the reference drug, celecoxib, inhibited edema by 85.2%.

**Figure 54:**
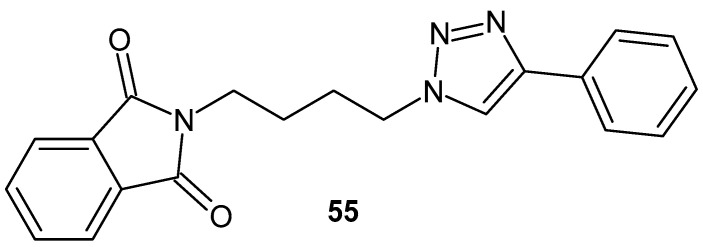
4-Phenyl-1-[4-(phthalimido-4-yl)butyl]-1*H*-1,2,3-triazole **55** reported by Assis et al. [105].

*N*-substituted phthalimide derivatives exhibited interesting anti-inflammatory effects, and studies showed that activity increased with the size of the aliphatic chain [105]. 4-Phenyl-1-[4-(phthalimido-4-yl)butyl]-1*H*-1,2,3-triazole **55** (Figure 54) demonstrated the highest anti-inflammatory activity, reducing carrageenan-induced paw edema by 48.9% after 4 h and by 22.4% after 24 h. It was superior to the standard acetylsalicylic acid (ASA) by 41.5% after 4 h and to ibuprofen by 45.2%. SAR studies show that the phthalimide and triazole fragments play an important role in the design of potent anti-inflammatory compounds.

**Figure 55:**
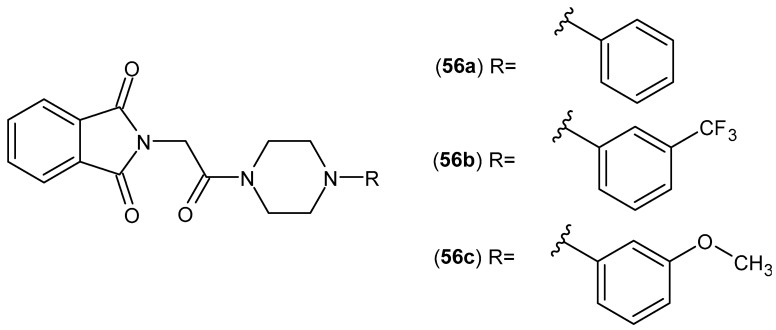
Isoindoline-1,3-dione derivatives **56a**–**c** reported by Szkatuła et al. [106].

Isoindoline-1,3-dione derivatives **56** (Figure 55), synthesized by Szkatuła et al. [106], showed stronger affinity for COX-2 than for COX-1. Compounds **56a**–**c** showed stronger COX-2 inhibition than the reference compound, meloxicam. The selectivity of compound **56a** (COX-1 IC_50_ = 76.7 µM, COX-2 IC_50_ = 53.4 µM, COX-2/COX-1 0.70) is comparable to that of meloxicam (COX-1 IC_50_ = 83.7 µM, COX-2 IC_50_ = 59.2 µM, COX-2/COX-1 0.71), whereas compounds **56b** (COX-1 IC_50_ = 171.0 µM, COX-2 IC_50_ = 47.6 µM, COX-2/COX-1 0.28) and **56c** (COX-1 IC_50_ = 176.4 µM, COX-2 IC_50_ = 43.8 µM, COX-2/COX-1 0.25) showed almost three times higher selectivity than the reference drug, meloxicam.

**Figure 56:**
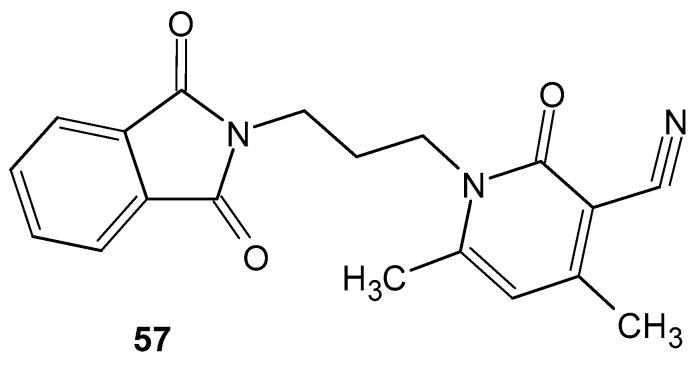
1-(3-(1,3-Dioxoisoindolin-2-yl)propyl)-4,6-dimethyl-2-oxo-1,2-dihydropyridine-3-carbonitrile **57** reported by Singh et al. [107].

Singh et al. [107] synthesized phthalimide derivatives and analyzed the resulting compounds for docking at the active site of the COX-2 enzyme, comparing them with the standard drugs, lumiracoxib and nimesulide. Structure-activity analysis (SAR) showed that almost all compounds exhibited strong binding affinity for COX-2 compared with lumiracoxib. All compounds were subjected to in vivo anti-inflammatory studies using the carrageenan-induced paw edema model, with nimesulide as the reference drug. 1-(3-(1,3-Dioxoisoindolin-2-yl)propyl)-4,6-dimethyl-2-oxo-1,2-dihydropyridine-3-carbonitrile **57** (Figure 56) showed maximum anti-inflammatory activity; after 30 min the inhibition of edema was 11.4%, after 90 min 20.4%, and after 180 min 9.1%, while the standard drug (nimesulide) inhibited it after 30 min to 13.7%, after 90 min to 23.5%, and after 180 min to 13.7%.

**Figure 57:**
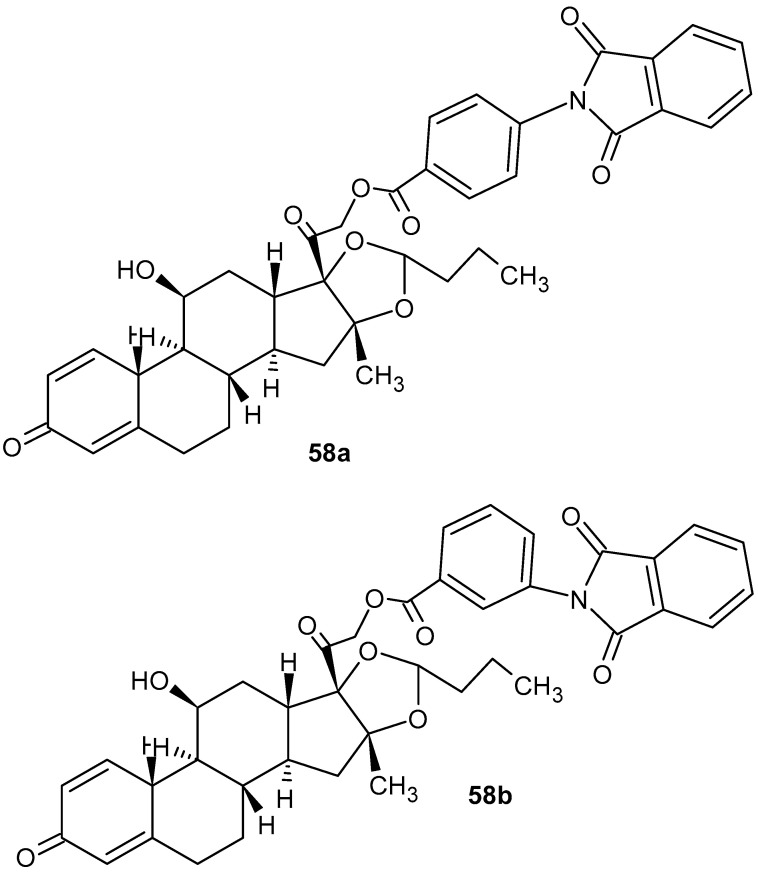
Glucocorticoid derivatives **58a**–**b** reported by Machado et al. [108].

Glucocorticoid derivatives were synthesized by Machado et al. [108], and their anti-inflammatory effects were evaluated in rat models of ulcerative colitis. Two compounds, **58a** and **58b** (Figure 57), demonstrated complete ulcer regression in 83.3% and 75% of treated animals, respectively. These levels are higher than those observed with prednisolone.

**Figure 58:**
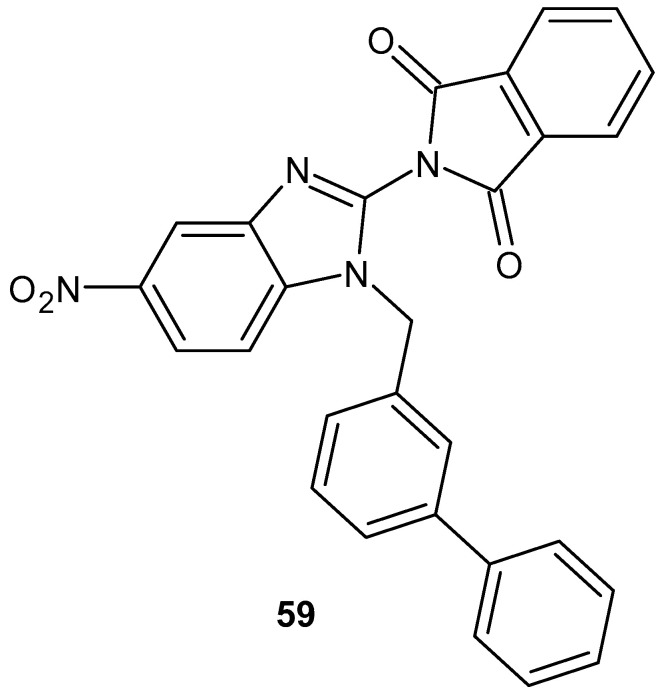
2-(1-([1,1′-Biphenyl]-3-ylmethyl)-5-nitro-1*H*-benzo[*d*]imidazol-2-yl)isoindoline-1,3-dione **59** reported by Kaur et al. [109].

The benzimidazole derivatives synthesized by Kaur et al. [109], benzimidazole derivatives combined with phthalimide, were tested for their analgesic and anti-inflammatory activity. The most active compound, **59** (Figure 58), was docked with COX-2 and 5-LOX, which was confirmed by in vivo studies. The benzimidazole and phthalimide derivative **59** showed interesting analgesic, anti-inflammatory, and broad inhibitory activity on COX-1/2 and 5-/15-LOX enzymes (COX-1 IC_50_ = 9.85 µM; COX-2 IC_50_ = 1.00 µM; SI = 9.85; 5-LOX IC_50_ = 0.32 µM; 15-LOX IC_50_ = 1.02 µM).

**Figure 59:**
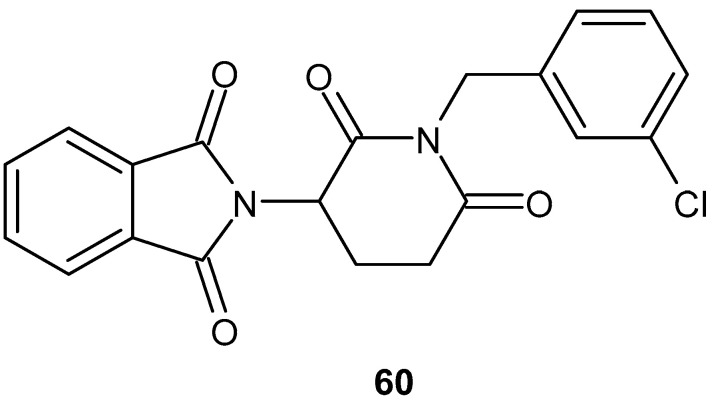
2-[1-(3-Chlorobenzyl)-2,6-dioxopiperidin-3-yl]isoindoline-1,3-dione **60** reported by Tang et al. [110].

Tang et al. [110] synthesized a series of thalidomide derivatives that were evaluated for their anti-inflammatory activity. The most promising compound, which showed no significant cytotoxicity at a concentration of 10 μM, was 2-[1-(3-chlorobenzyl)-2,6-dioxopiperidin-3-yl]isoindoline-1,3-dione **60** (Figure 59). This derivative inhibited the expression of IL-6 (69.44%) and TNF-α (75.01%) in HaCaT cells with greater potency than thalidomide, which inhibited IL-6 (48.70%) and TNF-α (22.97%).

**Figure 60:**
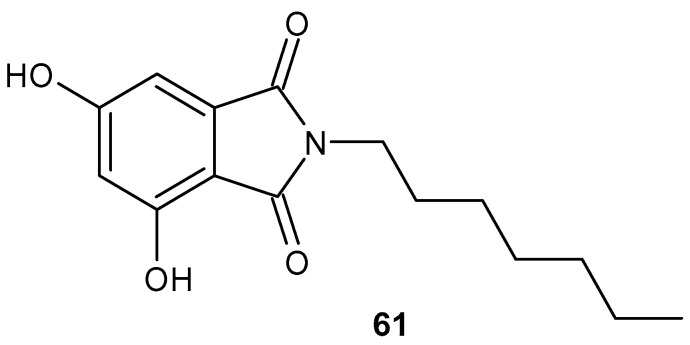
*N*-heptyl-3,5-dihydroxyphthalimide **61** reported by Bach et al. [111].

Based on the hypothesis that overproduction of nitric oxide (NO) plays a significant role in inflammatory processes, in 2017 scientists synthesized a series of phthalimide derivatives and tested their anti-inflammatory properties using lipopolysaccharide (LPS)-stimulated NO production in cultured RAW 264.7 mouse macrophage cells [111]. *N*-heptyl-3,5-dihydroxyphthalimide **61** (Figure 60) demonstrated the greatest inhibitory activity with an IC_50_ of 8.7 µg/mL. Subsequent studies showed that the inhibitory activity of compound **61** correlated with a decrease in mRNA and protein expression of LPS-stimulated inducible nitric oxide synthase (iNOS).

**Figure 61:**
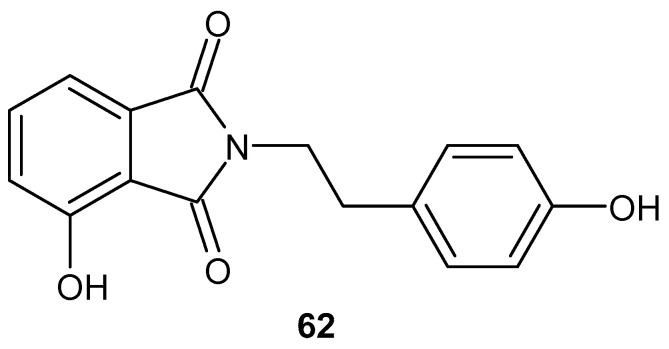
4-Hydroxy-2-(4-hydroxyphenethyl)isoindoline-1,3-dione **62** reported by Xiao et al. [112].

Xiao et al. [112] synthesized 4-hydroxy-2-(4-hydroxyphenethyl)isoindoline-1,3-dione **62** (Figure 61), which showed significant activation of PPAR-γ in rat liver Ac2F cells. The new derivative, **62**, is also characterized by low toxicity and anti-inflammatory effects in vivo [112,113,114]. Based on previous research, Su et al. [115] decided to check the anti-inflammatory properties of compound **62**. Compound **62** was first found to be non-toxic after exposure to concentrations up to 50 μM for 24 h using human embryonic kidney cells (HEK293T), rat liver Ac2F cells, and RAW264.7 mouse macrophages. Subsequent studies showed that **62** derivatives inhibited the induction of pro-inflammatory factors, including inducible nitric oxide synthase (iNOS), nitric oxide (NO), cyclooxygenase 2 (COX-2), tumor necrosis factor (TNF-α), interleukin-1 (IL-1), and interleukin-6 (IL-6), in RAW 264.7 mouse macrophages stimulated with lipopolysaccharide (LPS). The test of compound **62** (Figure 59) at 50 μM significantly inhibited the mRNA levels of iNOS, COX-2, TNF-α, IL-1β, and IL-6 to an extent comparable to 10 μM dexamethasone [115]. In subsequent studies, the phthalimide derivative **62** was evaluated for α-glucosidase inhibition in vitro. Compound **62** inhibited the enzyme (IC_50_ = 0.669 µM) better than acarbose (IC_50_ = 49.133 µM). In an in vivo study of antidiabetic activity, type 2 diabetes was induced by a high-fat diet (20%) and streptozotocin (30 mg/kg) in golden hamsters. The experiment showed that compound **62** exhibits α-glucosidase-inhibiting activity in vitro and antidiabetic activity in vivo [113].

**Figure 62:**
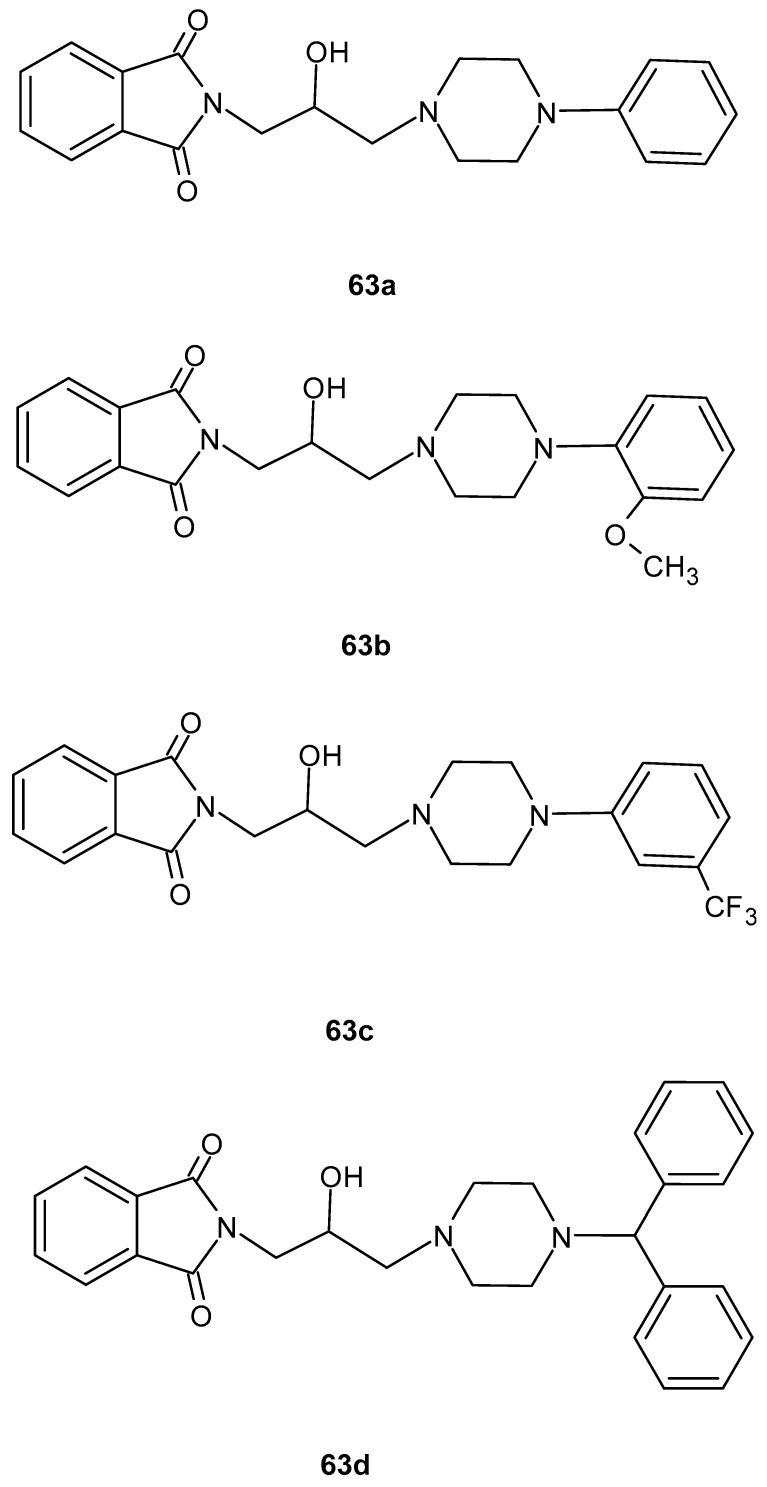
2-Hydroxy-3-(4-aryl-1-piperazinyl)propylphthalimide derivatives **63a–d** reported by Dziubina et al. [116].

The new derivatives of 2-hydroxy-3-(4-aryl-1-piperazinyl)propylphthalimide **63a**–**d** (Figure 62) synthesized by Dziubina et al. [116] were evaluated for their bioavailability and analgesic activity. The obtained results showed that all compounds strongly suppressed peripheral pain, while derivatives **63a**–**c** suppressed central/supraspinal pain to a lesser extent. In in vitro studies, all derivatives **63** showed anti-inflammatory activity, as demonstrated by studies in which the tested compounds reduced COX-2 levels in RAW 264.7 cells activated with lipopolysaccharide.

**Figure 63:**
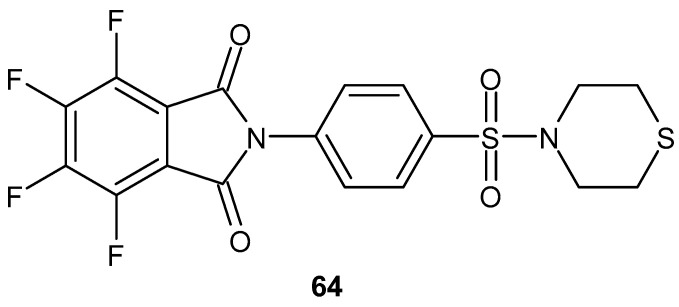
4,5,6,7-Tetrafluoro-2-(4-(thiomorpholinosulfonyl)phenyl) isoindoline-1,3-dione **64** reported by Barbosa et al. [117].

4,5,6,7-Tetrafluoro-2-(4-(thiomorpholinosulfonyl)phenyl) isoindoline-1,3-dione **64** (Figure 63), obtained by Barbosa et al. [117], demonstrated a 50% inhibition of TNF-α production in mice, whereas thalidomide caused a 33% inhibition at a screening concentration of 100 µM.

**Figure 64:**
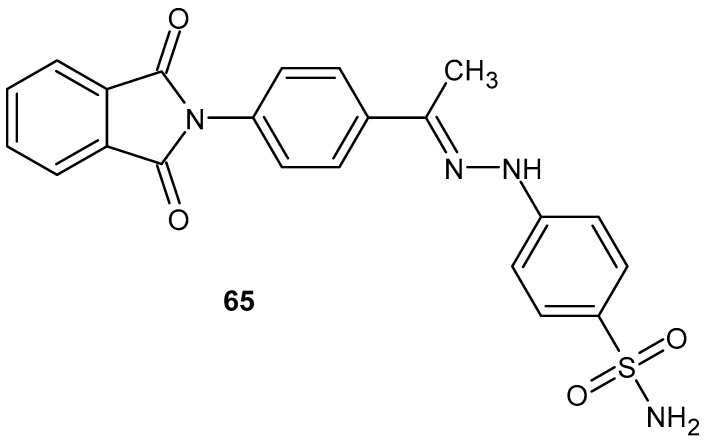
4-(*N*ʹ-{1-[4-(1,3-Dioxo-1,3-dihydro-isoindol-2-yl)-phenyl]-ethylidene}-hydrazino)-benzenesulfonamide **65** reported by Lamie et al. [118].

Lamie et al. [118] synthesized new phthalimide derivatives and tested them in vitro for antimicrobial, antioxidant, and anti-inflammatory activity. The cytotoxicity of these new compounds was also assessed in cancer cell lines and normal human cells. None of the obtained derivatives demonstrated cytotoxic activity. One compound, **65** (Figure 64), demonstrated the highest anti-inflammatory activity in vitro (32% reduction). This study was performed by ELISA on pretreated human umbilical vein endothelial cells (HUVECs), where these derivatives could inhibit NF-κB.

**Figure 65:**
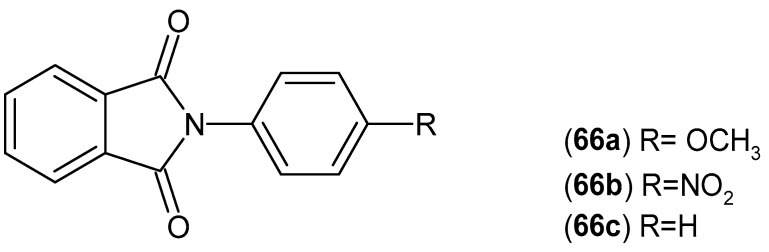
*N*-phenyl-phthalimide derivatives **66a**–**c** reported by Perveen and Orfali [119].

New *N*-phenylphthalimide derivatives **66a**–**c** (Figure 65) were synthesized and tested for their antioxidant, anti-inflammatory, and lipoxygenase enzyme inhibition activities by Perveen and Orfali [119]. Derivatives **66a** and **66b** showed strong antioxidant activity (**66a** IC_50_ = 27.3 μM and **66b** IC_50_ = 25.0 μM) compared to the reference compound BHA (IC_50_ = 44.2 μM). All compounds showed weak hydrogen peroxide scavenging activity. At the same time, compounds **66a** and **66b** showed potent lipoxygenase inhibitory activity (IC_50_ = 21.34 μM and IC_50_ = 20.45 μM, respectively), where standard baicalein showed inhibitory activity with an IC_50_ = 22.60 μM. Only compound **66c** showed interesting anti-inflammatory properties. The maximum inhibition of albumin denaturation for this derivative was 77.73 ± 0.35% at a concentration of 1 mg/mL, while aspirin showed a maximum inhibition of 95.89 ± 0.06% at a concentration of 0.20 mg/mL.

**Figure 66:**
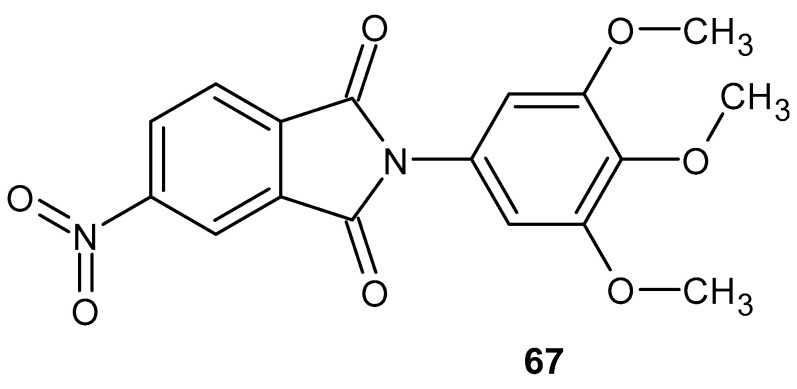
5-Nitro-2-(3,4,5-trimethoxyphenyl)isoindoline-1,3-dione **67** reported by Abdel-Azis et al. [120].

The phthalimide derivatives obtained by Abdel-Azis et al. [120] were tested in vivo for their anti-inflammatory effects in a carrageenan-induced rat paw edema model. Among the tested derivatives, 5-nitro-2-(3,4,5-trimethoxyphenyl)isoindoline-1,3-dione **67** (Figure 66) turned out to be the best, showing inhibition of COX-2 at IC_50_ = 0.1 µM and COX-1 at IC_50_ = 40 µM, and the selectivity was at the level of SI = 400, where celecoxib showed SI > 333.3. Docking of the obtained compounds at the COX-2 binding site was also performed. The most active derivative, **67**, showed a similar COX-2 binding pattern to the known compound SC-558, which is a selective COX-2 inhibitor.

**Figure 67:**
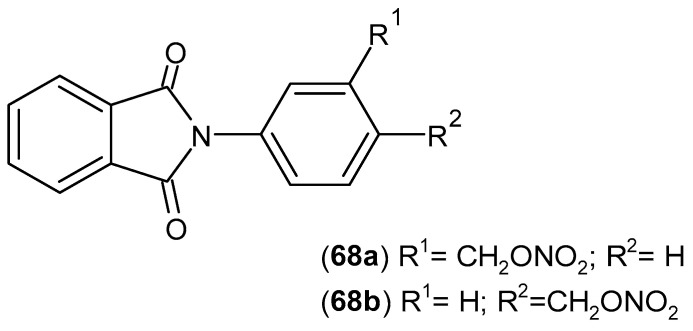
3/4-(1,3-Dioxo-1,3-dihydro-2*H*-isoindol-2-yl)benzyl nitrates **68a**–**b** reported by Santos et al. [121].

Santos et al. [121] designed and synthesized new thalidomide derivatives. Of the six compounds evaluated in vitro and in vivo for the treatment of sickle cell disease symptoms, two derivatives, **68a**–**b** (Figure 67), demonstrated the best analgesic, anti-inflammatory, and NO donor properties. Compound **68b** was the most active analgesic, reducing acetic acid-induced abdominal cramps by 66%. Compounds **68a**–**b** administered at a dose of 300 μmol/kg reduced TNF-α levels, similarly to dexamethasone.

**Figure 68:**
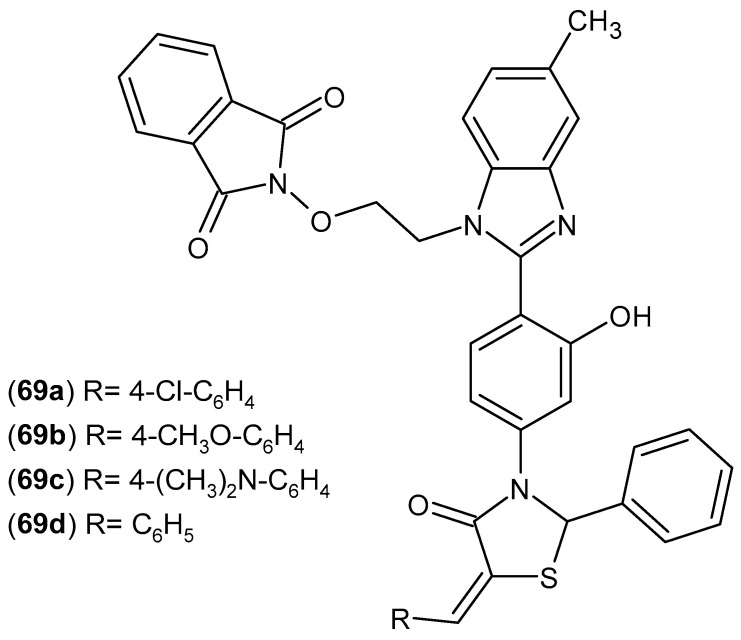
3-[4-(1*H*-4-methylbenzimidazol-2-yl)-2-hydroxyphenyl]-1-*N*-ethoxyphthalimido-5-(arylidene)-2-phenyl-1,3-thiazolidin-4-one derivatives **69a**–**d** reported by Kumar et al. [122].

Kumar et al. [122] synthesized four new derivatives of 3-[4-(1*H*-4-methylbenzimidazol-2-yl)-2-hydroxyphenyl]-1-*N*-ethoxyphthalimido-5-(arylidene)-2-phenyl-1,3-thiazolidin-4-one **69a**–**d** (Figure 68). The resulting compounds were tested for their anti-inflammatory and antibacterial effects. The anti-inflammatory activity of all newly synthesized derivatives **69a**–**d** was determined in a carrageenan-induced rat paw edema model. Initial paw volume was measured, as well as paw volume 3 and 6 h after carrageenan administration. The percentage inhibition of paw edema was calculated and compared with that of indomethacin. Compound **69d** showed similar inhibition (after 3 h: 1.62 ± 0.27%, after 6 h: 70.98%) to the reference drug, Indomethacin (after 3 h: 1.78 ± 0.340%, after 6 h: 66.44%).

**Figure 69:**
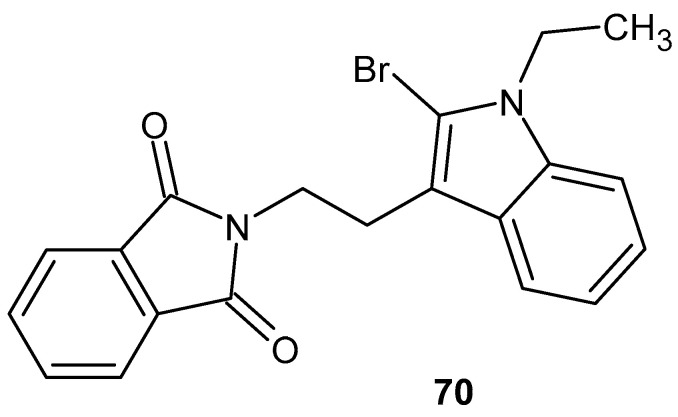
2-[2-(2-Bromo-1-ethyl-1*H*-indol-3-yl) ethyl]-1*H*-isoindole-1,3(2*H*)-dione **70** reported by El-Aaraga et al. [123].

El-Aaraga et al. [123] tested the obtained 2-[2-(2-bromo-1-ethyl-1*H*-indol-3-yl) ethyl]-1*H*-isoindole-1,3(2*H*)-dione **70** (Figure 69) on liver damage induced by carbon tetrachloride in mice, using thalidomide as the reference drug. Histopathological examination showed that, compared to the untreated and thalidomide-treated groups, the derivative **70** reduced levels of malondialdehyde, nitric oxide (NO), vascular endothelial growth factor (VEGF), TNF-α, and NF-κB.

**Figure 70:**
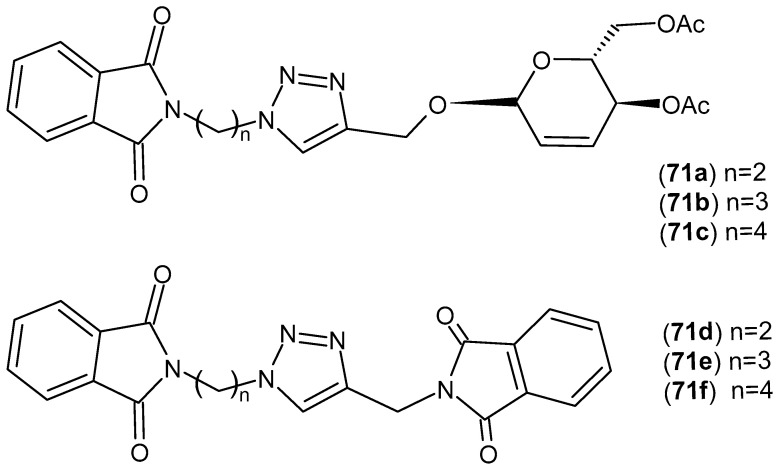
1,2,3-Triazolophthalimide derivatives **71a**–**f** reported by Assis et al. [124].

Six 1,2,3-triazolophthalimide derivatives **71a**–**f** (Figure 70) were synthesized by Assis et al. [124]. Three compounds were 1,2,3-triazoles combined with unsaturated carbohydrates and phthalimides **71a**–**c**, and the other three were 1,2,3-triazoles combined with phthalimides **71d**–**e**. The resulting derivatives were tested for their anti-inflammatory activity. Inflammation was induced by injecting carrageenan into the plantar tissue of the right hind paws of Swiss white mice. All six compounds, **71a**–**f**, demonstrated significant anti-inflammatory activity. Derivative **71b** demonstrated the best activity, reducing edema by 69%, while derivative **71f** achieved 56.2%.

**Figure 71:**
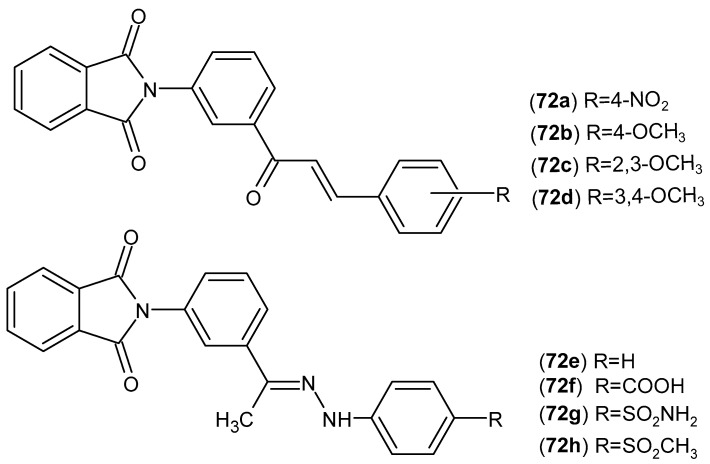
Phthalimide derivatives **72a**–**h** reported by Labib et al. [125].

Labib et al. [125] designed and obtained new phthalimide derivatives, **72a**–**h** (Figure 71), that were tested for their anti-inflammatory and analgesic activity. The in vivo anti-inflammatory activity was assessed using the formalin-induced rat paw edema test. It turned out that the most active derivatives showed very good anti-inflammatory activity in vivo in inhibiting edema (after 1 h: 41.7–50%, after 3 h: 40.7–67.4%, after 6 h: 20–46.7%), while the reference drug, diclofenac, showed worse inhibition of edema (after 1 h: 29.2%, after 3 h: 22.2%, after 6 h: 20.6%). Compound **72d** demonstrated the best anti-inflammatory activity (45.8–59.3%) and an increased thermal pain threshold (50–92.85%) comparable to piroxicam (75%), although it is a moderate selective COX-2 inhibitor (SI = 103).

**Figure 72:**
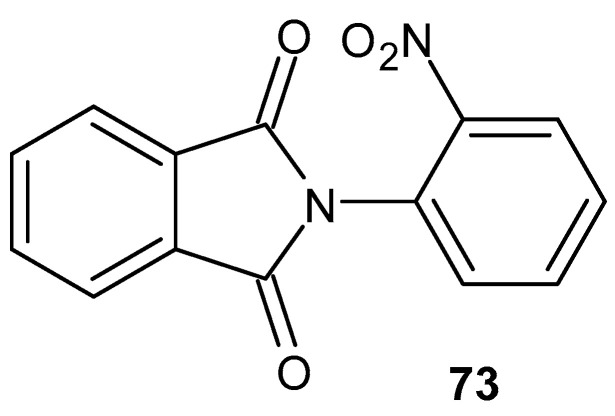
2-Nitrophenylphthalimide **73** reported by Assis et al. [126].

2-Nitrophenylphthalimide **73** (Figure 72) was synthesized and tested for its anti-inflammatory effects [126]. Its lipid-lowering and acute anti-inflammatory effects were compared with phthalimide in 3-month-old male Swiss mice. Compound **73** inhibits edema by 67%, comparable to the reference compound, ibuprofen, which inhibited edema by 66%, while aspirin inhibited edema by 58%.

**Figure 73:**
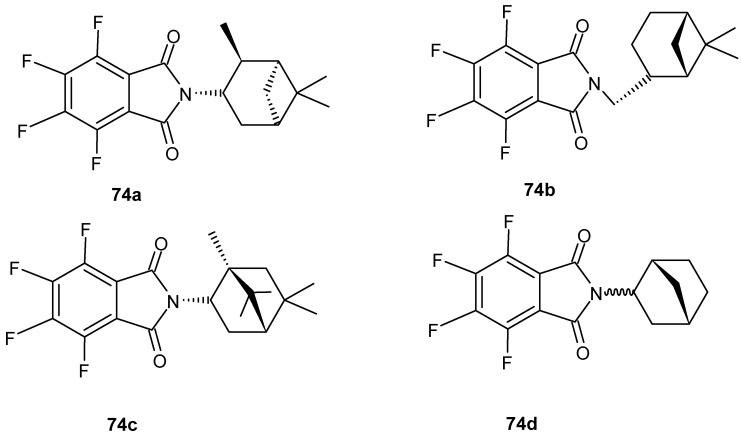
Monoterpenoid fluorophthalimides **74a**–**d** reported by Luo et al. [127].

Monoterpenoid fluorophthalimides were synthesized by Luo et al. [127] in the search for new drugs for the treatment of multiple myeloma (MM); four compounds **74a**–**d** (Figure 73) were obtained. Compounds **74a**–**d** showed excellent anti-inflammatory activity in RAW 264.7 cells infected with LPS (60 ng/mL), significantly reducing nitrite and/or TNF-α levels. The new derivatives, **74a**–**d**, also demonstrated significant anti-angiogenic activity in human umbilical vein endothelial cells and antiproliferative activity against lenalidomide-sensitive (MM.1S) and -resistant (U266 R10R) MM cells.

**Figure 74:**
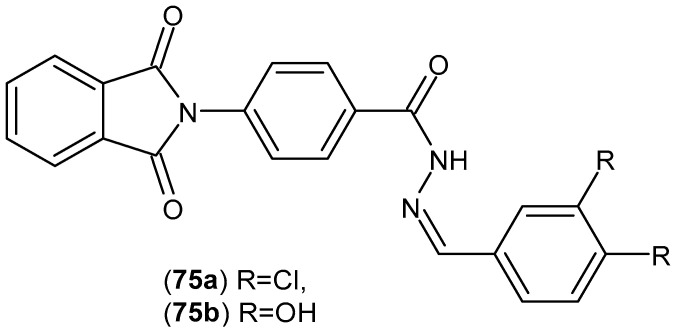
Schiff bases of phenylphthalimides **75a**–**b** reported by Bhat et al. [128].

Schiff bases of phenylphthalimides designed by Bhat et al. [128] were evaluated for anti-inflammatory activity using an in vitro model of inflammation induced by bacterial lipopolysaccharide (LPS) in murine RAW 264.7 cells. The obtained compounds were evaluated for their inhibitory activity on cyclooxygenase-2 (COX-2) and inducible nitric oxide synthase (iNOS) expression. All compounds, at a 10 µM concentration, reduced COX-2 and iNOS expression from 100% to ranges of 61.9 ± 5.1–84.5 ± 4.1% and 41.1 ± 5.4–85.3 ± 1.9%. Compound **75a** (Figure 74) demonstrated the strongest inhibition of iNOS expression (41.1 ± 5.4%) compared to the reference, dexamethasone (23.0 ± 3.3%). This compound was evaluated for its neuroprotective activity. Compound **75b** (Figure 74) demonstrated greater than 70% relative neuroprotection against 6-hydroxydopamine (6-OHDA)-induced cell death while maintaining the highest rate of cell viability (83.3 ± 4.5%).

**Figure 75:**
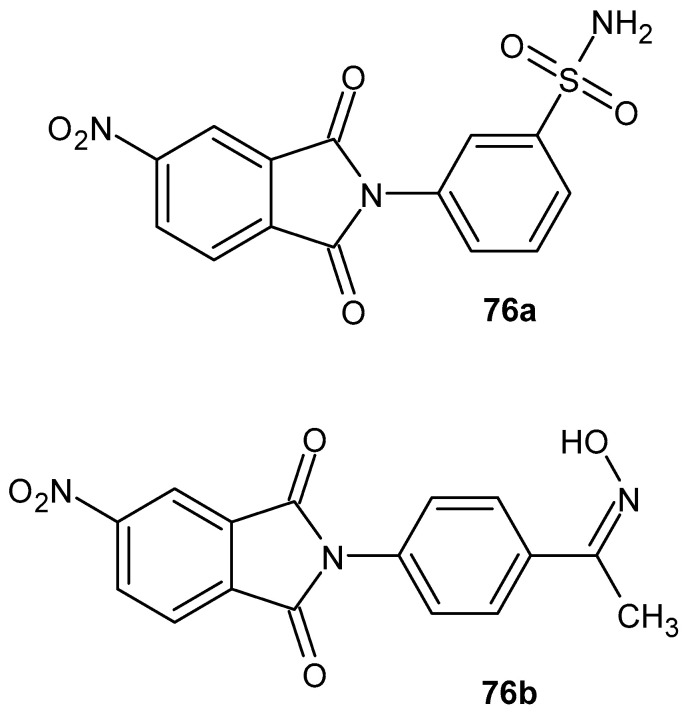
5-Nitroisoindoline-1,3-dione derivatives **76a**–**b** reported by Abdel-Aziz et al. [54].

Abdel-Aziz et al. [54] synthesized a series of phenylphthalimide derivatives and assessed their anti-inflammatory and ulcerogenic activity in vivo and their cytotoxic activity. As a result of testing the anti-inflammatory activity of all compounds on carrageenan-induced paw edema in rats, two derivatives proved to be the most active. 3-(5-Nitro-1,3-dioxo-1,3-dihydro-2*H*-isoindol-2-yl)benzene-1-sulfonamide **76a** and 2-(4-(1-(hydroxyimino)ethyl)phenyl)-5-nitroisoindoline-1,3-dione **76b** (Figure 75) demonstrated edema inhibition of 80.9% and 82.95%, respectively, while the reference drugs, celecoxib and diclofenac, inhibited edema by 85.6% and 83.4%, respectively. The derivatives **76a**–**b** were also subjected to an in vitro cyclooxygenase (COX-1/COX-2) enzyme inhibition test. Both compounds **75a** and **76b** demonstrated high selectivity for COX-1 (IC_50_ > 50 µM), COX-2 (IC_50_ = 0.15 µM), and SI > 333.3 compared to celecoxib (COX-1 IC_50_ > 50 µM, COX-2 IC_50_ = 0.129 µM, SI > 387.6). Compounds **76a**–**b** were also subjected to molecular docking studies, confirming that they possess the highest recognition at the COX-2 binding site.

**Figure 76:**
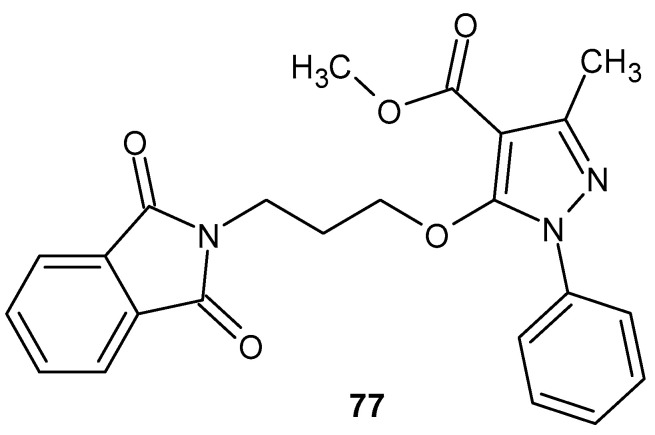
5-[3-(1,3-dioxo-1,3-dihydro-isoindol-2-yl)-propoxy]-3-methyl-1-phenyl-1*H*-pyrazole 4-carboxylic acid methyl ester **77** reported by Shrivastava et al. [129].

A group of pyrazole derivatives was synthesized and tested for their anti-inflammatory activity by Shrivastava [129]. 5-[3-(1,3-dioxo-1,3-dihydroisoindol-2-yl)-propoxy]-3-methyl-1-phenyl-1*H*-pyrazole-4-carboxylic acid methyl ester **77** (Figure 76) was found to have a potent inhibitory effect on the cyclooxygenase-2 (COX-2) enzyme. It was observed that the derivative **77** exhibited better activity than the reference drug, nimulide, but the ethyl ester of compound **77**, 5-[3-(1,3-dioxo-1,3-dihydro-isoindol-2-yl)-propoxy]-3-methyl-1-phenyl-1*H*-pyrazole-4-carboxylic acid ethyl ester, did not exhibit anti-inflammatory activity.

**Figure 77:**
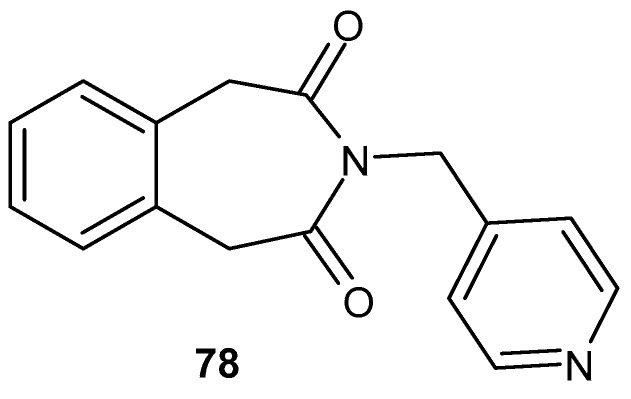
3-[(Pyridin-4-yl)methyl]-1*H*-3-benzazepine-2,4(3*H*,5*H*)-dione **78** reported by Sondhi et al. [130].

Sondhi et al. [130] synthesized 3-[(pyridin-4-yl)methyl]-1*H*-3-benzazepine-2,4(3*H*,5*H*)-dione **78** (Figure 77), which showed a better anti-inflammatory effect than the standard drug, phenyl butazone (19% at a dose 25 mg/kg p.o.), using the carrageenan-induced paw edema model.

#### 2.2.2. Bicyclic Imides Fused to a Heterocyclic Ring

The search for compounds with potential anti-inflammatory activity has also been conducted among bicyclic derivatives containing an imide ring fused with a second heterocyclic ring, such as a pyrrole, pyridine, or pyrazine. Structural rigidification resulting from ring fusion may influence both target selectivity and pharmacodynamic properties, making such systems attractive scaffolds in medicinal chemistry.

**Figure 78:**
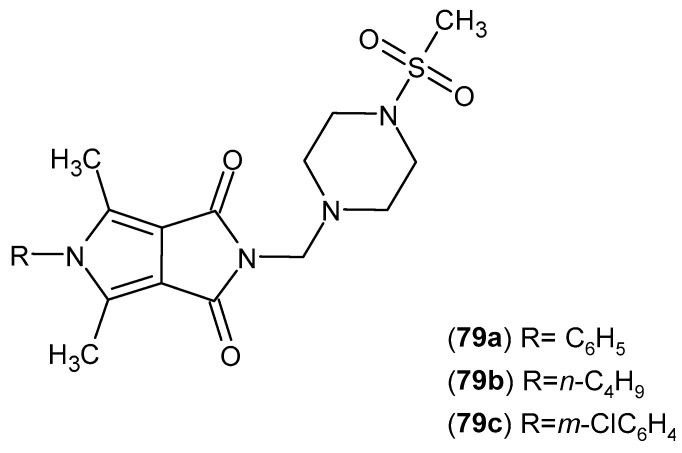
Pyrrolopyrrole-2,5-dione derivatives **79a**–**c** reported by Redzicka et al. [131].

In studies on pyrrolopyrrole-2,5-dione derivatives with potential anti-inflammatory activity, a series of Mannich bases of this scaffold were designed and synthesized, followed by evaluation of their biological activity as inhibitors of cyclooxygenase isoenzymes (COX-1 and COX-2). IC_50_ values were determined for both isoenzymes, and selectivity indices expressed as IC_50_(COX-2)/IC_50_(COX-1) were calculated. Meloxicam was used as the reference compound. The highest selectivity toward COX-2 was observed for compounds **79a**–**c** (Figure 78), with selectivity indices of 0.24, 0.26, and 0.28, respectively, indicating approximately two-fold higher selectivity compared with meloxicam (0.55) [131].

**Figure 79:**
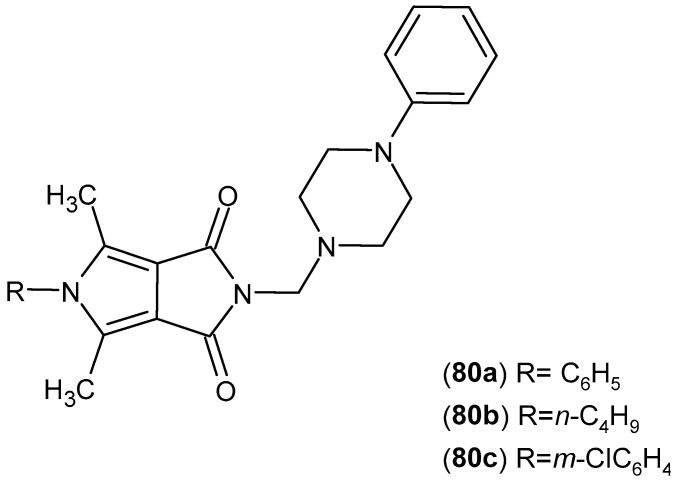
Pyrrolopyrrole-2,5-dione derivatives **80a**–**c** reported by Szczukowski et al. [132].

For structures **79** and **80** (Figure 78 and Figure 79), the authors expanded the scope of the pharmacological investigations [132].

The biological activity of structures **79** and **80** was evaluated against 15-lipoxygenase (15-LOX). All six compounds exhibited enzyme inhibition comparable to that of zileuton. The IC_50_ values for 15-LOX were as follows: **79a**—1.25 μM, **79b**—1.30 μM, **79c**—1.28 μM, **80a**—1.45 μM, **80b**—1.50 μM, and **80c**—1.55 μM.

Antioxidant studies were also performed in NHDF cells. After 24 h of incubation, derivatives **79a**–**79c** and **80a**–**80c** reduced the levels of reactive oxygen species (ROS) by an average of 45–60% compared with the control, and they reduced reactive nitrogen species (RNS) levels by 35–50%. The fast halo assay demonstrated a 40–55% reduction in DNA strand breaks, while derivatives **79a** and **79b** additionally protected lipids against nitric oxide-induced peroxidation by approximately 30–35% [132].

**Figure 80:**
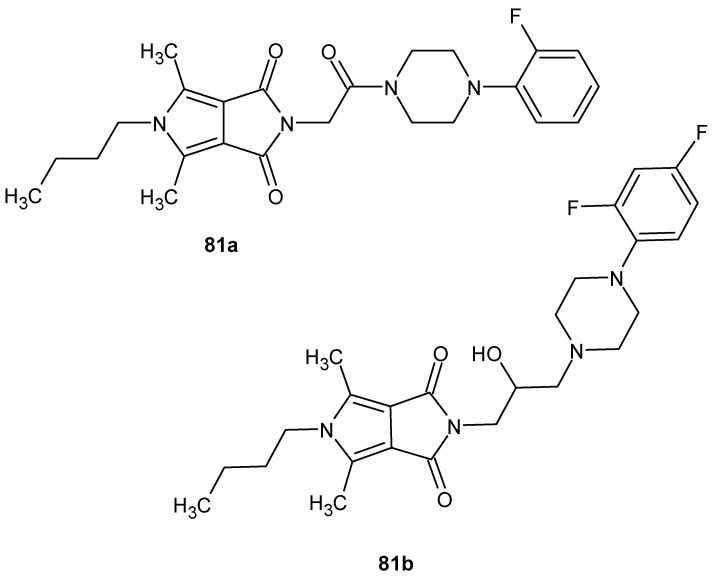
Butyl derivatives of pyrrolo[3,4*-c*]pyrrole-1,3(2*H*,5*H*)-diones **81a** and **81b** reported by Redzicka et al. [133].

Figure 80 presents the structures of two anti-inflammatory active butyl-substituted derivatives of pyrrolo[3,4*-c*]pyrrole-1,3(2*H*,5*H*)-diones, **81a** and **81b** [133,134]. The anti-inflammatory activity of compound **81a** was evaluated in vitro using a colorimetric cyclooxygenase inhibition assay [133].

Compound **81a** exhibited selective inhibition of the COX-2 isoform, with an IC_50_ value for COX-2 lower than that of the reference drug, meloxicam, whereas inhibition of COX-1 occurred only at significantly higher concentrations. In addition, in a cellular model of rheumatoid arthritis, compound **81a** demonstrated a cytoprotective effect, reducing the levels of reactive oxygen and nitrogen species and limiting DNA strand damage, particularly at a concentration of 10 µM. Cell viability studies indicated no significant cytotoxicity of compound **81a** within the concentration range associated with its anti-inflammatory activity, suggesting a favorable biological profile [133].

In contrast, the butyl derivative **81b** was identified as the most active compound in the series of 2-[2-hydroxy-3-(4-substituted-1-piperazinyl)propyl]pyrrolo[3,4-c]pyrrole-1,3(*2H*,5*H*)-diones, exhibiting higher selectivity toward COX-2 compared with meloxicam [133]. Moreover, compound **81b** displayed inhibitory activity against the lipoxygenase (LOX) enzyme. Dual inhibition of COX-2 and LOX, particularly the 5-LOX and 15-LOX isoforms, has emerged in recent years as a major focus of anti-inflammatory drug research, as compounds possessing such properties have demonstrated enhanced efficacy, a broader spectrum of action, and a reduced risk of adverse effects compared with classical nonsteroidal anti-inflammatory drugs (NSAIDs) [134].

**Figure 81:**
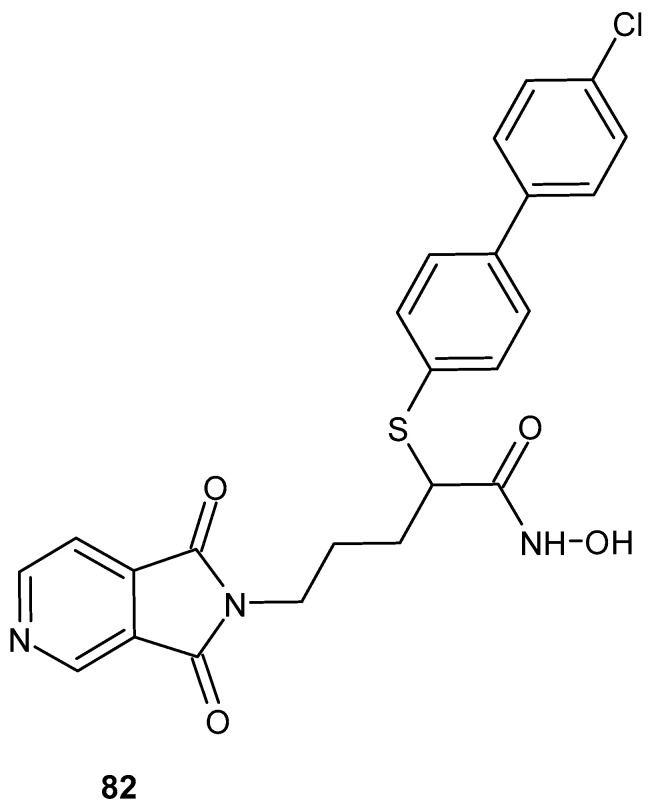
5-(1,3-Diokso-1,3-dihydro-2*H*-pirolo[3,4-*c*]pirydyn-2-ylo)-2-{[(4′-chloro[1,1′-bifenyl]-4-ylo)sulfanylo]metylo}*-N*-hydroksypentanamid **82** reported by Chollet et al. [135].

Research on bicyclic maleimide derivatives with potential anti-inflammatory activity, in which the imide ring is fused with a pyridine ring, was initiated by Chollet et al. [135]. This group synthesized 5-(1,3-dioxo-1,3-dihydro-2*H*-pyrrolo[3,4-*c*]pyridin-2-yl)-2-{[(4′-chloro[1,1′-biphenyl]-4-yl)sulfanyl]methyl}-*N*-hydroxypentanamide **82** (Figure 81) as an inhibitor of matrix metalloproteinases (MMPs), exhibiting IC_50_ values of 3nM for MMP-2, 12nM for MMP-9, and 84nM for MMP-13.

Matrix metalloproteinases (MMPs) are zinc-dependent proteolytic enzymes involved in the degradation and remodeling of the extracellular matrix. Elevated levels of MMPs can lead to various pathologies, including metastatic cancer and arthritis; therefore, inhibition of these enzymes is considered an important therapeutic strategy [135].

**Figure 82:**
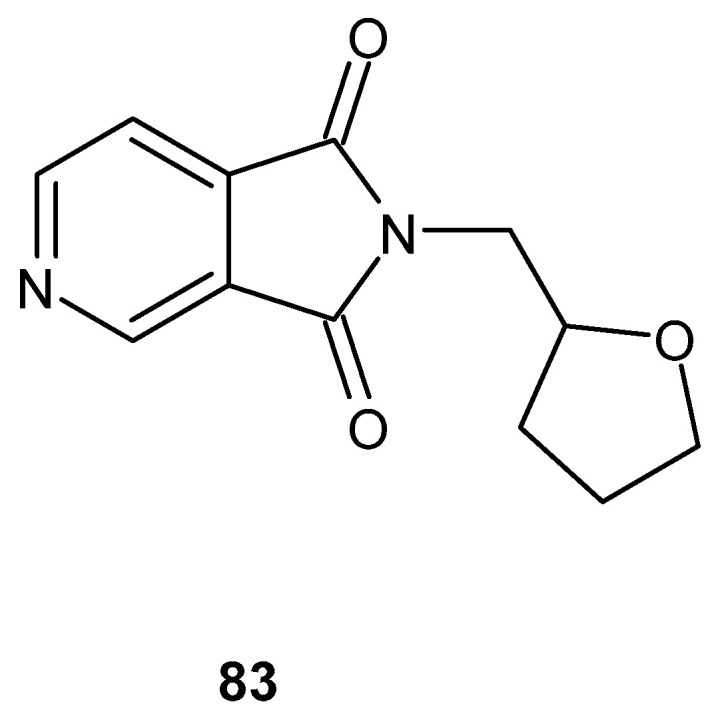
2-(tetrahydrofuran-2-ylmethyl)-2*H*-pyrrolo[3,4-*c*]pyridine-1,3-dione **83** reported by Sondhi et al. [136].

Sondhi et al. [136] synthesized 2-(tetrahydrofuran-2-yl)methyl)-2*H*-pyrrolo[3,4-*c*]pyridine-1,3-dione **83** (Figure 82) and evaluated its anti-inflammatory activity. Compound **83** exhibited 26% activity at an oral dose of 50 mg/kg.

**Figure 83:**
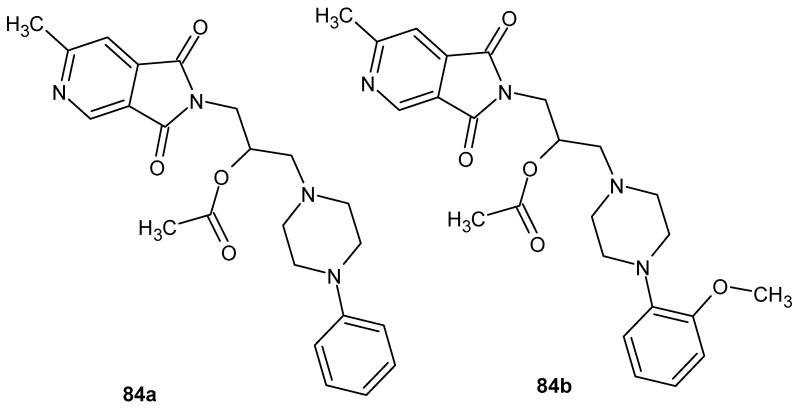
Derivatives of 1*H*-pyrrolo[3,4-*c*]pyridine-1,3(2*H*)-diones **84a** and **84b** reported by Dziubina et al. [137].

Dziubina et al. [137] published a study aimed at investigating the potential analgesic, anti-edematous (anti-inflammatory), and anti-allodynic effects of two 1*H*-pyrrolo[3,4-*c*] pyridine-1,3(2*H*)-dione derivatives **84a** and **84b** (Figure 83) in various experimental pain models.

The authors conducted a series of pharmacological tests, including the hot plate test, the capsaicin test, the oxaliplatin-induced allodynia test, and the formalin test. The formalin test is used to identify tonic inflammatory pain and involves two phases of stimulus perception. The first phase results from the transmission of nociceptive stimuli along C fibers, whereas the second phase is a consequence of the developing inflammatory response and central sensitization to peripheral stimulation. In this assay, compounds **84a** and **84b** (5–20 mg/kg) exhibited analgesic effects in both phases, with a more pronounced effect observed in the second phase of the test. The potential analgesic effects of compounds **84a** and **84b** on neurogenic pain were investigated using a mouse model of capsaicin-induced pain. Both compounds **84a** and **84b** reduced capsaicin-induced nociceptive behaviors in a dose-dependent manner. The authors also performed in vitro studies using the RAW 264.7 cell line.

Compounds **84a** and 84**b** significantly reduced COX-2 levels in lipopolysaccharide (LPS)-stimulated cells. These results suggest that the tested compounds possess at least partial anti-inflammatory properties [137].

**Figure 84:**
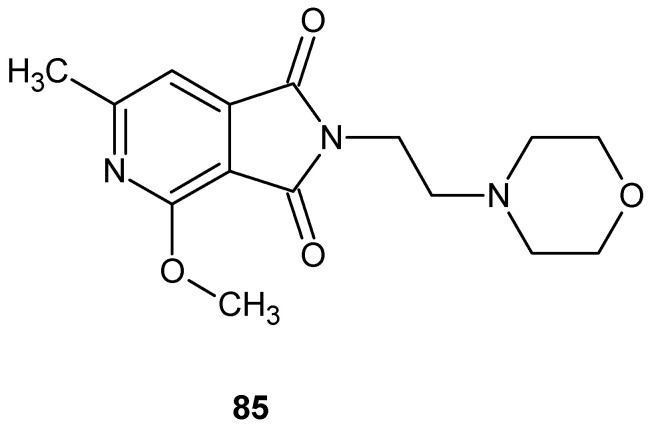
44-Methoxy–*N*-[2-(N-morpholine)-ethyl]-6-methyl-1H-[pyrrolo[3.4-c]pyridine-1,3(2H)-dione **85** reported by Krzyżak et al. [138].

Krzyżak et al. [138] synthesized new *N*-substituted derivatives of 1*H*-pyrrolo[3,4-*c*]pyridine-1,3(2*H*)-diones. These compounds were evaluated for their anti-inflammatory activity by assessing their ability to inhibit COX-1/COX-2 interactions with bovine serum albumin (BSA). In vitro COX-1 and COX-2 inhibition assays were performed, and the pharmacological results demonstrated that all synthesized compounds exhibited the potential to inhibit both enzymes. The highest selectivity toward COX-2 was observed for compound **85** (Figure 84), for which the COX selectivity ratio (IC_50_(COX-2)/IC_50_(COX-1)) was 0.55, compared with 0.71 for meloxicam [138].

**Figure 85:**
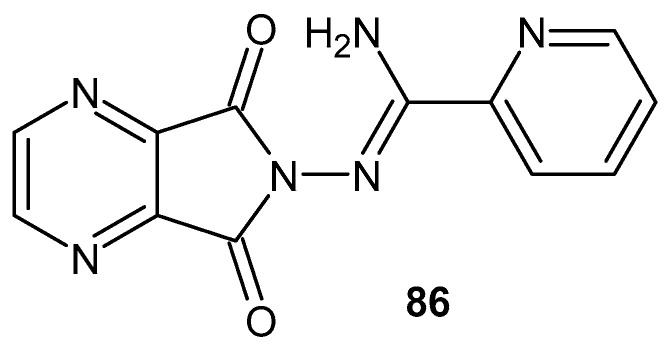
*N*’-(5,7-dioxo-5,7-dihydro-6*H*-pyrrolo[3,4-*b*]pyrazin-6-yl)pyridine-2-carboximidamide **86** reported by Kumar et al. [139].

Kumar et al. [139] designed and synthesized bicyclic derivatives in which the imide ring is fused with a pyrazine ring. The compounds obtained were evaluated for their anti-inflammatory and anticancer activities. The most active compound, *N*′-(5,7-dioxo-5,7-dihydro-6*H*-pyrrolo[3,4-*b*]pyrazin-6-yl)pyridine-2-carboximidamide **86** (Figure 85), exhibited 35% anti-inflammatory activity at an oral dose of 50 mg/kg. In contrast, the reference drug, ibuprofen, showed 39% activity at the same dose (50 mg/kg, p.o.).

Compound **86** also demonstrated anticancer activity against tumor cell lines in vitro, including breast T47D (IC_50_ = 13.22 ± 1.74 µM), lung NCI-H-522 (IC_50_ = 15.16 ± 1.91 µM), colon HCT-15 (IC_50_ = 67.63 ± 3.75 µM), ovary PA-1 (IC_50_ = 16.88 ± 4.13 µM), and liver HepG-2 (IC_50_ = 38.39 ± 2.78 µM) [139].

#### 2.2.3. Bicyclic Imides Fused to an Unsaturated Ring

**Figure 86:**
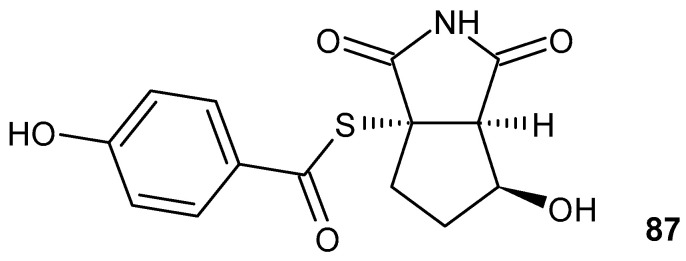
NitrosporeusineA**87** isolated by Yang et al. [140].

Nitrosporeusines A **87** (Figure 86) was isolated from an arctic actinomycete, *Streptomyces nitrosporeus*, a compound that has a cyclopenta[*c*]pyrrolidinodione skeleton [140] and which shows potential in treating a wide range of diseases. The review [141] indicates a strong link between inflammation and the development of acute nephritis in patients with sepsis. Studies [142] have shown that Nitrosporeusine A **87** attenuates sepsis-related acute kidney injury by reducing the expression of renal PGC-1a, which is dependent on activation of the IL-6/sIL-6R axis.

**Figure 87:**
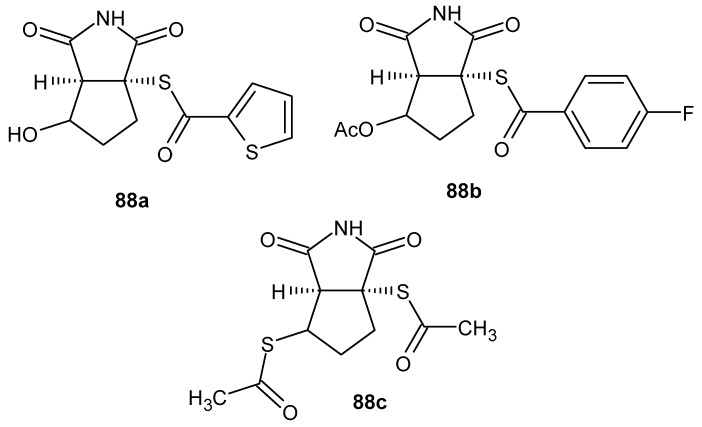
Cyclopenta[*c*]pyrrolidinodione derivative compounds **88a**–**c** reported by Philkhana et al. [143].

Cyclopenta[*c*]pyrrolidinodione derivatives (Nitrosporeusines A and B analogues) were evaluated for their anti-inflammatory activity [143]. The most potent compounds **88a**–**c** (Figure 87) significantly reduce the level of nitric oxide, reactive oxygen species, and pro-inflammatory cytokines, and they also inhibit the action of pro-inflammatory mediators (inducible nitric oxide synthase, cyclooxygenase-2, and nuclear factor-κB).

### 2.3. Polycyclic Imides

#### 2.3.1. Naphthalimide Derivatives

Naphthalimides are a significant class of aromatic heterocycles composed of a cyclic diimide and a naphthalene skeleton. The benzisoquinoline-1,3-(2*H*)-dione moiety is found in compounds with anti-inflammatory properties.

**Figure 88:**
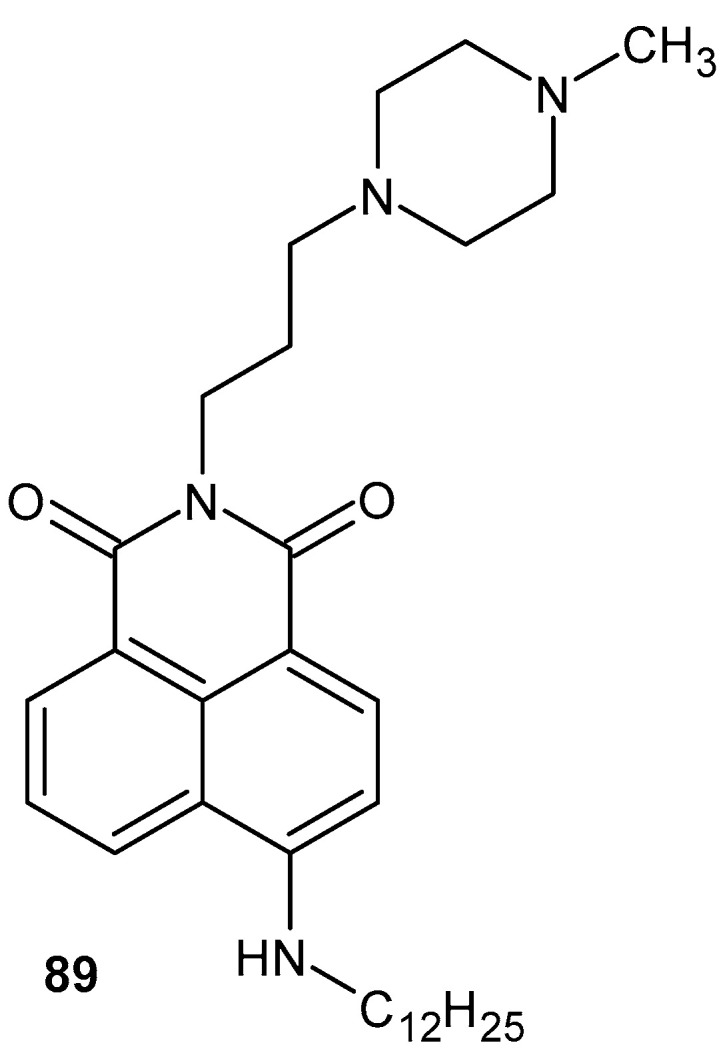
6-(Dodecylamino)-2-(3(4-methylpiperazin-1-yl)propyl)-1*H*-benzo-[*de*]isoquinoline-1,3(2*H*)-dione **89** reported by Shao et al. [144].

6-(Dodecylamino)-2-(3(4-methylpiperazin-1-yl)propyl)-1*H*-benzo[*de*]isoquinoline-1,3(2*H*)-dione **89** (Figure 88) inhibits inflammatory responses induced by phorbol 12-myristate 13-acetate/phytohemagglutinin (PMA/PHA) by the inhibition of NF-κB DNA-binding and expression of pro-inflammatory cytokines such as IL-2, IL-1β, IL-6, TNF-α, and IFN-γ in T lymphoma cells [144]. Compound **89** also inhibits LPS-induced production of inflammatory mediators (COX-2 and iNOS).

**Figure 89:**
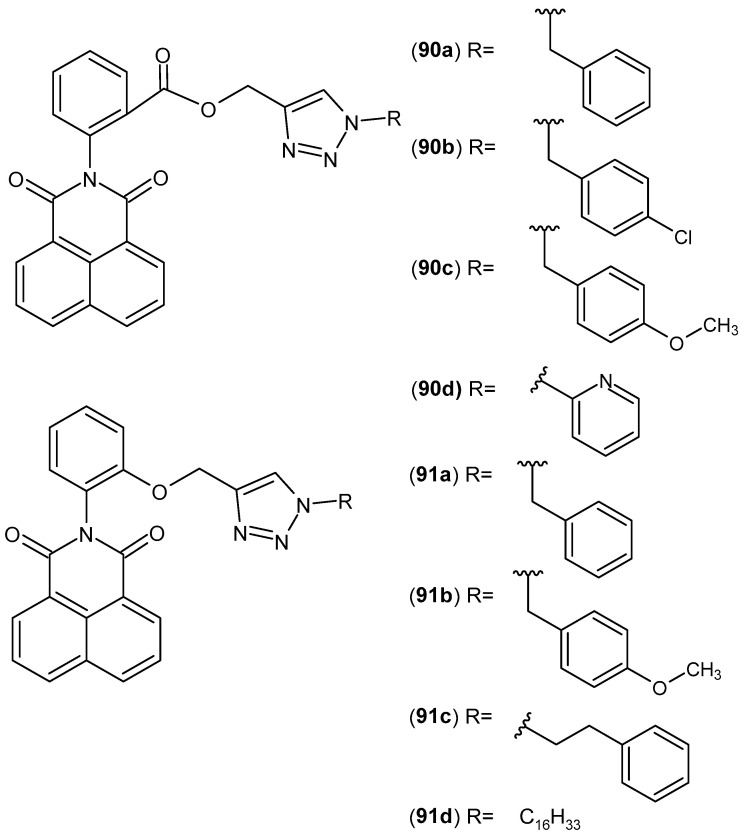
Naphthalimide derivatives **90**–**91** reported by Begam et al. [145].

Naphthalimide derivatives with a triazole moiety have been synthesized and evaluated for their anti-inflammatory activity. Esters **90a**–**d** were more potent than ethers **91a**–**d** (Figure 89) [145]. Molecular docking studies against COX-1 and COX-2 show that (1-benzyl-1*H*-1,2,3-triazol-4-yl)methyl-2-(1,3-dioxo-1*H*-benzo[*de*]isoquinolin-2(3*H*)-yl)benzoate **90a** exhibits the best inhibitory effect. The anti-inflammatory activity of compounds **90a**–**d** and **91a**–**d** was also evaluated in vitro. Derivative **90a** inhibits the denaturation of bovine serum albumin and egg albumin at 200 µM, comparable to the standard drug, diclofenac sodium [145].

**Figure 90:**
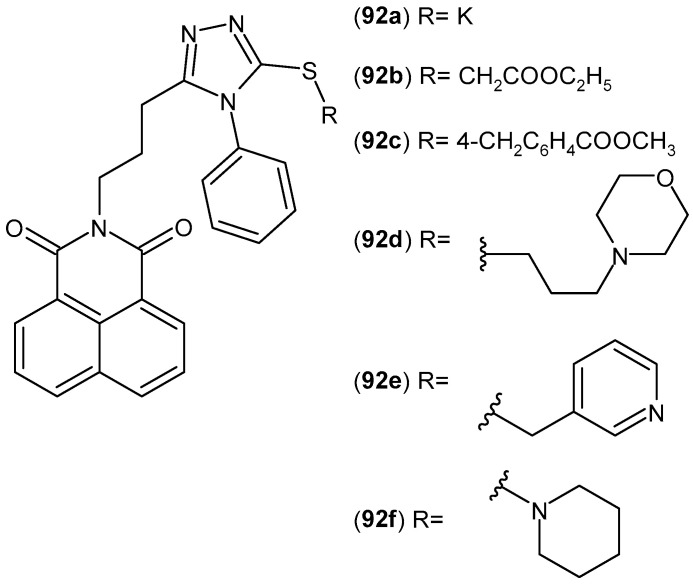
1,2,4-Triazole–1,8-naphthalimide derivatives **92a**–**f** reported by Korol et al. [146].

The anti-inflammatory potential of 1,2,4-triazole–1,8-naphthalimide derivatives **92a**–**f** (Figure 90) was assessed by IL-6 inhibition [146]. A significant reduction in IL-6 levels was observed. The most active compounds, **92a** and **92d**, reduced IL-6 levels to 7 pg/mL.

Using the fluorescent properties of naphthalimide, fluorophore conjugates with various anti-inflammatory drugs have been developed to monitor targeted therapy, thereby optimizing treatment efficacy and minimizing side effects.

**Figure 91:**
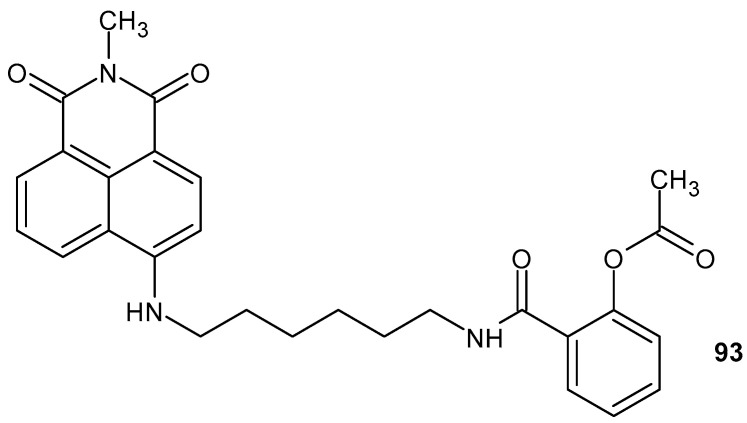
Naphthalimide and aspirin derivative **93** reported by Xia et al. [147].

Compound **93** (Figure 91) containing a naphthalimide scaffold linked to the COX-2 inhibitor aspirin has been developed [147]. Due to its fluorescent properties and low biotoxicity, compound **93** can specifically target cancer cells, inhibiting COX-2 expression with greater efficacy than aspirin.

**Figure 92:**
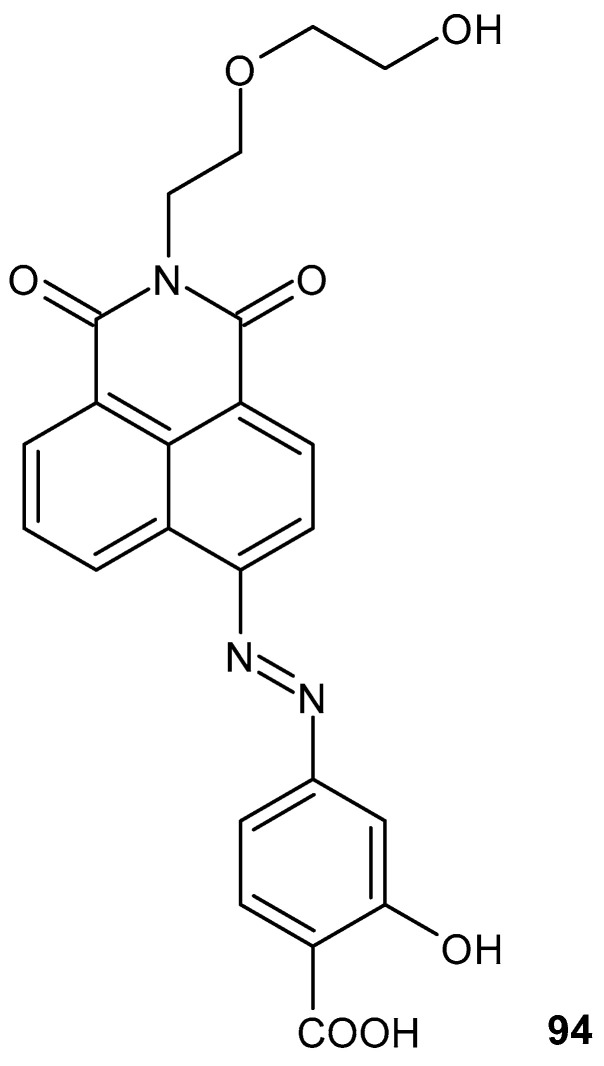
Prodrug **94** reported by Wang et al. [148].

Another fluorescent prodrug, **94** (Figure 92), was synthesized by conjugating 5-aminosalicylic acid with the naphthalimide fluorophore [148]. Compound **94** allows for real-time monitoring of drug activation while maintaining therapeutic efficacy.

#### 2.3.2. Other Polycyclic Imides

**Figure 93:**
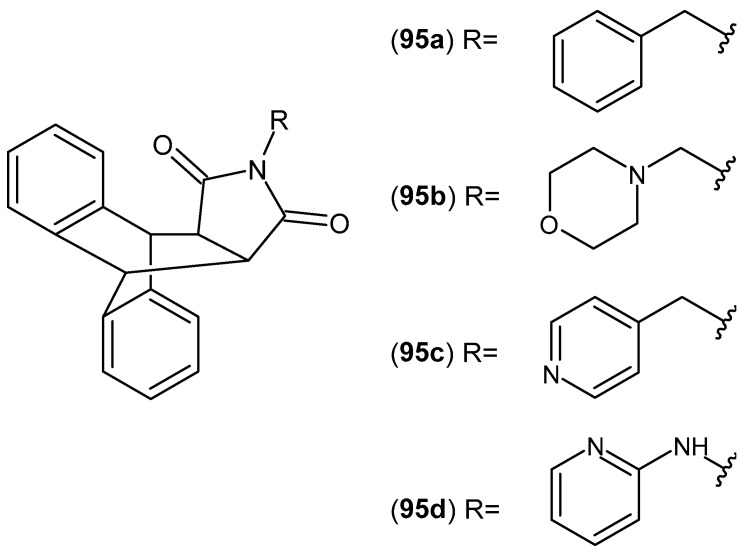
9,10-α,β-succinimide derivatives **95a**–**d** reported by Arya et al. [149].

Some of the obtained 9,10-dihydroanthracene-9,10-α,β-succinimide derivatives **95a**–**d** (Figure 93) exhibited anti-inflammatory activity similar to the standard drug, ibuprofen, at 50 mg/kg p.o. [149].

**Figure 94:**
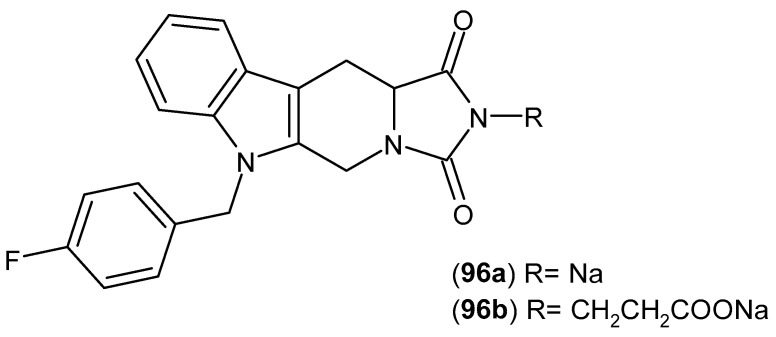
PAT compounds **96a**–**b** as ATX inhibitors reported by Stein et al. [150].

Among a series of PAT compounds developed by the Cayman Chemical Company and PharmAkea as autotaxin (ATX) inhibitors, (R)-6-(4-fluorobenzyl)-5,6,11,11a-tetrahydro-1*H*-imidazo[1′,5′:1,6]pyrido[3,4-*b*]indole-1,3(2*H*)-dione (PAT-494) **96a** (IC_50_ = 20 nM, LPC) and (S)-3-(6-(4-fluorobenzyl)-1,3-dioxo-5,6,11,11a-tetrahydro-1*H*-imidazo[1′,5′:1,6] pyrido[3,4-*b*]indol-2(3*H*)-yl)propanoic acid (PAT-352) **96b** (IC_50_ = 26 nM, LPC) were the most active (Figure 94) [150].

**Figure 95:**
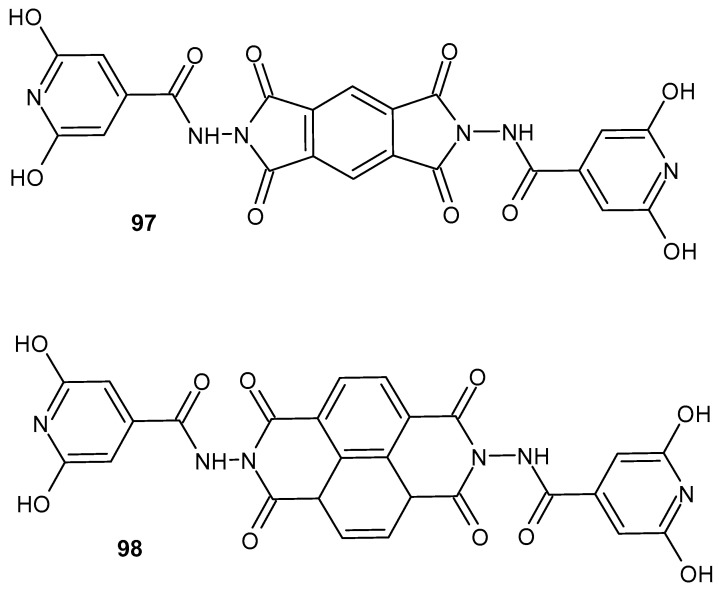
Policyclicbisimides **97** and **98** reported by Al-Omar et al. [151].

Policyclic bisimides **97** and **98** (Figure 95) were synthesized and evaluated for their anti-inflammatory activity in the carrageenan-induced rat paw edema protection test [151]. Both compounds **97** and **98** were found to be more potent than diclofenac potassium.

**Figure 96:**
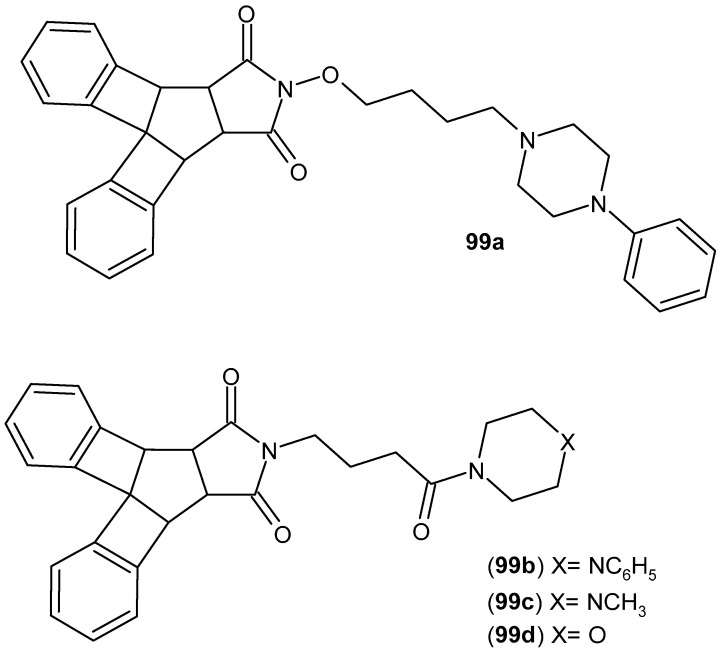
Benzeno-1*H*-benzo[*f*]isoindole-1,3(2*H*)-dione derivatives **99a**–**d** reported by Al-Omar et al. [151].

The anti-inflammatory activity of the synthesized benzeno-1*H*-benzo[*f*]isoindole-1,3(2*H*)-dione derivatives was evaluated by carrageenan-induced paw edema test in rats [151]. Compounds **99a**–**d** (Figure 96) were similarly potent to diclofenac sodium.

**Figure 97:**
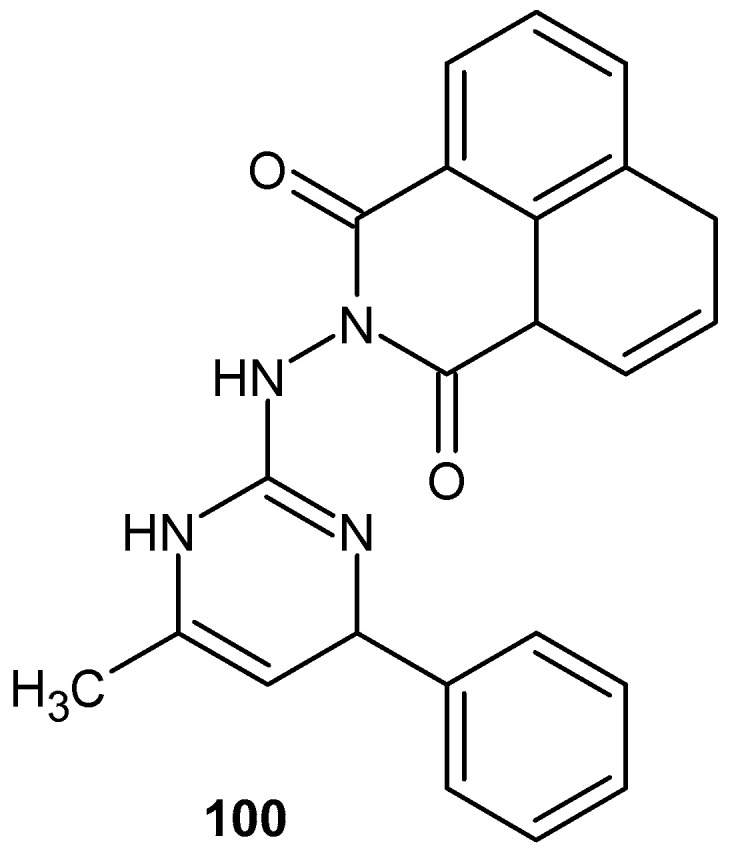
2-(6-Methyl-4-phenyl-1,4-dihydro-pyrimidin-2-ylamino)-benzo[*de*]isoquinoline-1,3-dione **100** reported by Said et al. [152].

2-(6-Methyl-4-phenyl-1,4-dihydro-pyrimidin-2-ylamino)-benzo[*de*]isoquinoline-1,3-dione **100** (Figure 97) was found to be more potent than prednisolone as an anti-inflammatory agent in the carrageenan-induced rat paw edema protection test (87.80% protection at a dose of 25 mg/kg) [152].

**Figure 98:**
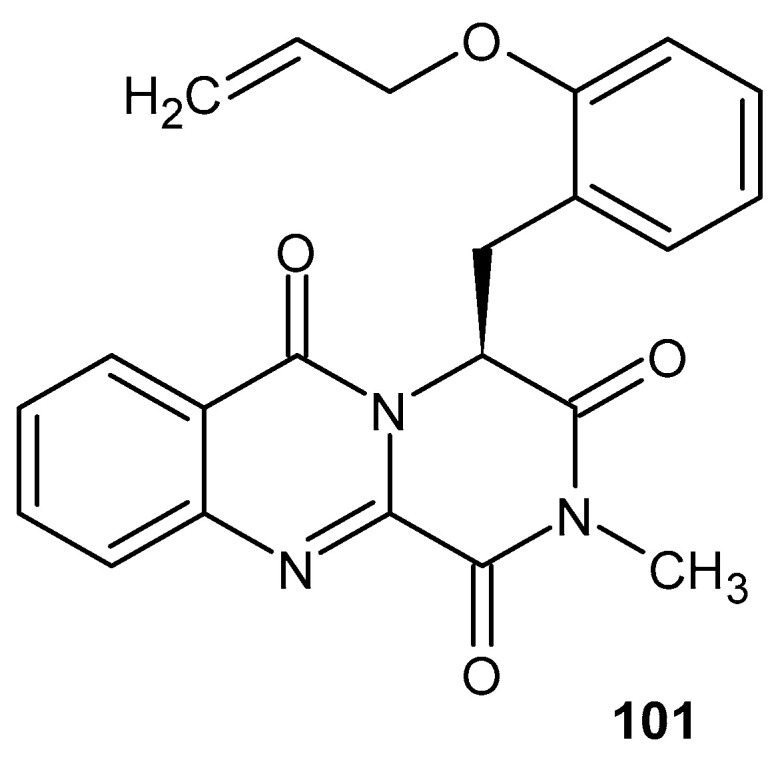
(4S)-17-Hydroxybrevianamide N derivative **101** reported by Zhou et al. [153].

17-Hydroxybrevianamide N derivative **101** was obtained by Zhou et al. [153] as a potent anti-inflammatory agent.(4S)-Enantiomer of **101** (Figure 98) is three times more effective than its (4R)-enantiomer in inhibiting NO concentrations, with an IC_50_ value of 0.5 μM. Compound (4S)- **101** demonstrated simultaneous inhibition of the levels of inflammatory factors (TNF-α, IL-1β, and IL-6) and reduced inflammation-induced increases in MAPK and NF-κB signaling proteins.

To improve the organization and provide a clearer overview of the reported compounds, the cyclic imide derivatives discussed in this review have been systematically classified according to their principal molecular mechanisms of action. A comprehensive summary of the corresponding molecular targets and representative compounds is provided in Table 1.

## 3. Conclusions

Cyclic imides constitute an important class of heterocyclic compounds with established clinical relevance and diverse pharmacological activity, largely determined by the nature and position of substituents at the imide nitrogen atom and within the imide scaffold. Representative examples include ethosuximide and methsuximide used in the treatment of epilepsy; antipsychotic agents such as lurasidone, perospirone, tandospirone, gepirone, and zalospirone; the antiviral drug tecovirimat; the anticancer agent tivantinib; immunomodulatory drugs (e.g., lenalidomide and pomalidomide); as well as compounds with pronounced anti-inflammatory activity, including thalidomide and apremilast.

This review summarizes literature reports on synthetic and natural compounds containing a cyclic imide ring that exhibit anti-inflammatory activity. The majority of studies focus on derivatives of the pyrrole-2,5-dione scaffold, frequently incorporated into bicyclic or polycyclic systems. However, other structurally diverse imide-containing classes, including succinimides, glutarimides, hydantoins, and naphthalimides, also demonstrate significant anti-inflammatory potential. The biological activity of these derivatives is primarily associated with suppression of pro-inflammatory mediators, including cytokines (e.g., TNF-α, IL-1β, and IL-6), inhibition of COX and LOX isoenzymes, reduction of LPS-induced nitric oxide production, modulation of chemokine secretion, and regulation of key signaling pathways such as NF-κB. A structured classification of the discussed compounds according to their principal molecular mechanisms of action is presented in Table 1.

Given the ongoing need for novel, effective, and well-tolerated anti-inflammatory agents, cyclic imides represent privileged scaffolds for further structural optimization and comprehensive pharmacological investigation. Continued research in this field is expected to facilitate the development of new therapeutically relevant compounds within this versatile chemical family.

## Figures and Tables

**Table 1:** Classification of cyclic imide derivatives according to their principal molecular mechanisms of anti-inflammatory action.

Principal Molecular Target/Pathway	Representative Compounds	Biological Effect	Experimental Model
Kinase-mediated signaling (Syk, GSK-3, MAPK**)**	**1**, **42**, **101**	Inhibition of pro-inflammatory kinase signaling; IL-6, MMP-3, MAPK activation	In vitro; cellular models
Chemokine receptor modulation (CXCR3)/ (CXCL)	**2a**–**d**, **18**	CXCR3/CXCL antagonism	Receptor assay
IDO pathway (hIDO-1)	**3**	Immunomodulatory activity via IDO inhibition	Enzymatic
Cyclooxygenase pathway (COX-1/COX-2)	**4a**–**b**, **6a**–**c**, **7**, **16a**–**e**, **27a**–**e**, **53**, **56a**–**c**, **57**, **59**, **63a**–**c**, **67**, **72d**, **75a**–**b**, **76a**–**b**, **77**, **79a**–**c**, **81a**–**b**, **84a**–**b**, **85**, **88a**–**c**, **89**, **90a**–**d**, **93**	Selective or dual COX inhibition; reduction of PGE_2_ level	Enzymatic; cellular; in vivo edema models
Lipoxygenase pathway (5-LOX/15-LOX)	**21a**–**b**, **59**, **79a**–**c**, **80a**–**c**, **81b**	LOX inhibition; attenuation of eicosanoid signaling	Enzymatic; cellular
NF-κB signaling axis	**8a**–**b**, **18**, **35**, **36a**, **38b**, **47a**, **65**, **70**, **88a**–**c**, **101**	Suppression of NF-κB activation; inflammatory gene transcription	LPS-stimulated cellular models
NO/iNOS pathway	**9a**–**b**, **12**, **18**, **35**, **36a**, **37a**–**d**, **38a**–**b**, **44a**–**b**, **49a**–**b**, **61**, **62**, **68a**–**b**, **70**, **101**	Inhibition of NO production; reduction of iNOS expression	Macrophage models; in vivo
Cytokine modulation (TNF-α, IL-6, IL-1β)	**6a**–**d**, **7**, **8**, **11a**–**b**, **15b**, **15d**, **20**, **33a**–**b**, **34**, **39**, **41**, **47a**, **50a**–**j**, **52**, **60**, **62**, **64**, **70**, **74a-d**, **92a**, **92d**, **101**	Suppression of pro-inflammatory cytokines	Cellular; in vivo inflammation models
Matrix metalloproteinases (MMPs)	**22**, **47a**, **82**	MMP inhibition; reduced tissue degradation	Enzymatic assays
TACE inhibition	**23a**–**b**, **24a**–**e**	Modulation of TNF-α maturation	Enzymatic assays
Autotaxin (ATX)	**96a**–**b**	ATX inhibition	Enzymatic assay
Nuclear receptor modulation (LXRα/β, PPAR-γ)	**50a**–**j**, **62**	Transcriptional anti-inflammatory regulation	Cellular; in vivo
Multikinase/STAT signaling	**46**, **47a**	Downregulation of cytokine signaling and oxidative stress	Cellular; in vivo
In vivo anti-inflammatory activity (target not specified)	**17**, **51a**–**b**, **55**, **69a**–**d**, **71a**–**f**, **78**, **100**	Reduction of edema and/or inflammatory activity	Carrageenan-induced paw edema; pain models

## Data Availability

No new data were created or analyzed in this study. Data sharing is not applicable to this article.

## Abbreviations

The following abbreviations are used in this manuscript:

AIA	Adjuvant-induced arthritis
ALI	Acute lung injury
AMES II	This assay is a 384-well microplate format modification of the classic bacterial reverse mutation test
AMPA	α-Amino-3-hydroxy-5-methyl-4-isoxazolepropionic
AP-1	Activator protein 1
BHA	Butylhydroxyanisole
Caco-2	Cell line of human colorectal adenocarcinoma cells
CAIs	Cholesterol absorption inhibitors
cAMP	Cyclic adenosine monophosphate
CCL2, CXCL1	Chemokines
CIA	Collagen-induced arthritis
COX-1, COX-2	Cyclooxygenases
CRP	C-reactive protein
CXCR3	Chemokine receptor
fMLP	N-formylmethionyl-leucyl-phenylalanine
GPCRs	G protein-coupled receptors
GPs	Glutarimide-containing polyketides
GTI	Gastrointestinal tissue index
hERG	Human ether-à-go-go-Related Gene
IDO	Indoleamine-pyrrole 2,3-dioxygenase
IL-6	Interleukin 6
IMiDs	Immunomodulatory drugs
IFN-γ	Cytokine interferon gamma
iNOS	Inducible nitric oxide synthase
LDH	Lactate dehydrogenase
LOX	Lipoxygenase
LPS	Lipopolysaccharide
MAPK	Mitogen-activated protein kinase
MDA	Malondialdehyde
Me-NFM	4-Methyl-N-phenylmaleimide
MM	Multiple myeloma
MMP	Matrix metalloproteinase
MNT	Mammalian cell micronucleus test
MPO	Myeloperoxidase
NF-κB	Nuclear factor kappa B
NS-398	Non-steroidal anti-inflammatory agent with analgesic and antipyretic effects
PAF	Platelet-activating factor
PBMCs	Stimulated peripheral blood mononuclear cells
PDE4	Inhibitor-phosphodiesterase 4
PGE_2_	Prostaglandin E2
PLC	Phospholipase C
PLSN	Partial ligation of the sciatic nerve
PMNLs	Polymorphonuclear leukocytes
ROS	Reactive oxygen species
SOD	Superoxide dismutase
STAT1, STAT2	Signal transducer and activator of transcription (1 or 2)
Syk	Spleen tyrosine kinase
TACE	TNFα-converting enzyme
TLR4	Toll-like receptor 4
TNF	Tumor necrosis factor
VEGF	Vascular endothelial growth factor
